# Silanol-Assisted High-Yield Nanofabrication of SnO_2_ Single Crystals with Highly Tunable and Ordered Mesoporosity

**DOI:** 10.1021/acscentsci.3c01374

**Published:** 2024-01-29

**Authors:** Shoukang Xiao, Li Wang, Ze Qin, Xiao Chen, Liyu Chen, Yingwei Li, Kui Shen

**Affiliations:** † Guangdong Provincial Key Laboratory of Fuel Cell Technology, School of Chemistry and Chemical Engineering, South China University of Technology, Guangzhou, Guangdong 510640, China; ‡ Beijing Key Laboratory of Green Chemical Reaction Engineering and Technology, Department of Chemical Engineering, Tsinghua University, Beijing 100084, China

## Abstract

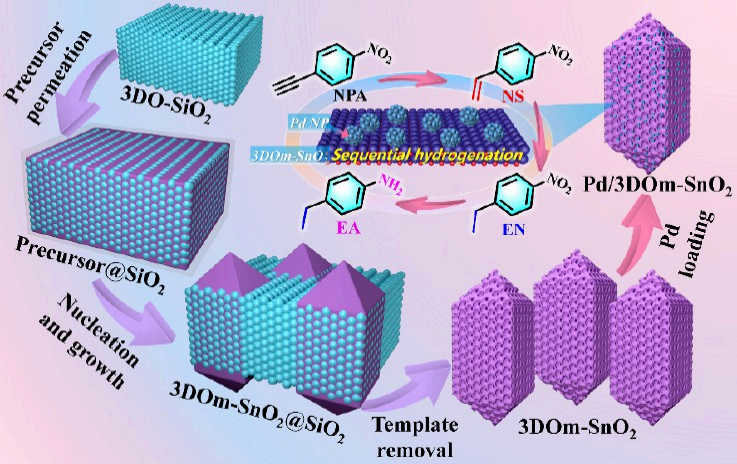

Highly ordered mesoporous materials with a single-crystalline structure have attracted broad interest due to their wide applications from catalysis to energy conversion/storage, but constructing them with good controllability and high yields remains a highly daunting task. Herein, we construct a new class of three-dimensionally ordered mesoporous SnO_2_ single crystals (3DOm-SnO_2_) with well-defined facets and excellent mesopore tunability. Mechanism studies demonstrate that the silanol groups on ordered silica nanospheres (3DO-SiO_2_) can induce the efficient heterogeneous crystallization of uniform SnO_2_ single crystals in its periodic voids by following the hard and soft acid and base theory, affording a much higher yield of ∼96% for 3DOm-SnO_2_ than that of its solid counterpart prepared in the absence of 3DO-SiO_2_ (∼1.5%). Benefiting from its permanent ordered mesopores and favorable electronic structure, Pd-supported 3DOm-SnO_2_ can efficiently catalyze the unprecedented sequential hydrogenation of 4-nitrophenylacetylene to produce 4-nitrostyrene, then 4-nitroethylbenzene, and finally 4-aminoethylbenzene. DFT calculations further reveal the favorable synergistic effect between Pd and 3DOm-SnO_2_ via moderate electron transfer for realizing this sequential hydrogenation reaction. Our work underlines the crucial role of silanol groups in inducing the high-yield heterogeneous crystallization of 3DOm-SnO_2_, shedding light on the rational design and construction of various 3DO single crystals that are of great practical significance.

## Abstract 2

A new class of highly ordered mesoporous SnO_2_ single crystals with high pore tunability is successfully constructed by using a novel silanol-assisted nanocasting strategy.

## Introduction

The development of three-dimensionally ordered mesoporous (3DOm) materials is of increasing importance for researchers in chemistry and materials science due to their attractive properties and consequent wide applications.^1−5^ Specially, as an important family of crystalline materials, the introduction of highly ordered mesopores into single-crystalline metal oxides would be expected to create a new family of porous materials, which could combine the advantages of 3DOm materials with the fascinating properties inherent to single-crystalline frameworks of metal oxides to endow them with unexpected performances in many important applications from catalysis to energy conversion/storage.^6^ In the past decades, various approaches have been developed to construct various 3DOm materials, in which the soft template approach has been proved to be an efficient strategy for producing various 3DOm architectures.^7,8^ However, this method easily yields 3DOm metal oxides with amorphous or polycrystalline walls, and only a few examples have be successful in synthesizing their single-crystalline frameworks, since most of the organic surfactants as structure-directing agents cannot tolerate the high temperatures required for the crystallization of metal oxides.^9−12^ Alternatively, a nanocasting strategy has been proven to be a powerful tool to fabricate 3DOm metal oxides with highly crystalline walls by using various 3DOm silica as a rigid template.^13^ Therefore, since the first report of crystalline Cr_2_O_3_, more than 20 types of metal oxides have been successfully nanocast into their 3D mesostructured forms, which afford excellent performances in numerous potential applications.^14−17^ Unfortunately, almost all of these syntheses rely on a crucial pyrolysis process to evoke material crystallization, which generally produces polycrystalline rather than single-crystalline metal oxides, as the high-temperature pyrolysis always causes particle sintering and aggregation, resulting in the loss of single-crystalline characteristics.^18,19^ So far, the construction of 3DOm single crystals of metal oxides with good controllability remains a highly desired but daunting task in materials science.

A hydrothermal method has been demonstrated to be a powerful technique for the synthesis of single-crystalline materials with good uniformity, high purity, controlled stoichiometry, and excellent reproducibility.^20^ In particular, limited reports have demonstrated that the integration of the hydrothermal technique with the nanocasting method provides an efficient strategy to prepare novel materials containing complex 3D mesostructures.^21,22^ However, a well-known challenge for this integrated strategy is how to overwhelm the homogeneous nucleation of precursors in bulk solution, thus guiding the heterogeneous nucleation and subsequent growth of crystals in the confined voids of templates, which is also the key to determine the yield of the resultant crystalline materials.^23^ In a notable recent contribution, a seed-assisted approach was developed to prepare a new type of anatase TiO_2_ single crystals with distinct facets and disordered mesopores.^24^ While this work is a beautiful realization of the heterogeneous crystallization of TiO_2_ single crystals in a silica bead template, it is not readily conducive to the synthesis of other materials, especially those with ordered mesopores.^25^ Additionally, several groups have also reported that the functionalization of mesoporous silica by organic groups such as −NH_2_ and −CH=CH_2_ could lead to a strong interaction between organic groups and metal ions, which can capture these metal ions from bulk solution and then crystallize them in/on templates.^14,26−29^ However, previous studies have focused on the assembly of various types of nanocrystals into their hollow structures that are naturally polycrystalline and irregular in porosity and/or morphology.^27−29^

Herein, we report the construction of 3DOm single crystals of metal oxides with an adjustable pore size and well-defined facets by proposing a facile silanol-assisted nanocasting strategy. As a proof of concept, SnO_2_, an extensively studied transition metal oxide that is widely utilized in a variety of important applications,^30^ was chosen in this work. We demonstrate that the silanol groups on 3D-ordered colloidal silica nanospheres (denoted as 3DO-SiO_2_) can induce the heterogeneous nucleation and growth of 3DOm SnO_2_ single crystals (denoted as 3DOm-SnO_2_) in their periodic voids by following the well-known hard and soft acid and base theory. To the best of our knowledge, this is the first successful synthesis of highly ordered mesoporous single crystals of metal oxides with well-defined facets and excellent mesopore tunability. Remarkably, this strategy can afford uniform 3DOm-SnO_2_ with a much higher yield of ∼96% than that of its solid counterpart (∼1.5%) prepared whithout using 3DO-SiO_2_, revealing a great potential for large-scale synthesis. We also demonstrate the potential application of 3DOm-SnO_2_ as an excellent support to anchor Pd nanoparticles (NPs) (denoted as Pd/3DOm-SnO_2_) to catalyze the sequential hydrogenation of 4-nitrophenylacetylene to produce 4-nitrostyrene, then 4-nitroethylbenzene, and finally 4-aminoethylbenzene. Density functional theory (DFT) calculations are further employed to elucidate the unique catalytic properties of Pd/3DOm-SnO_2_ for this reaction.

## Results and Discussion

### Synthesis and Characterization of 3DOm-SnO_2_

**Figure 1:**
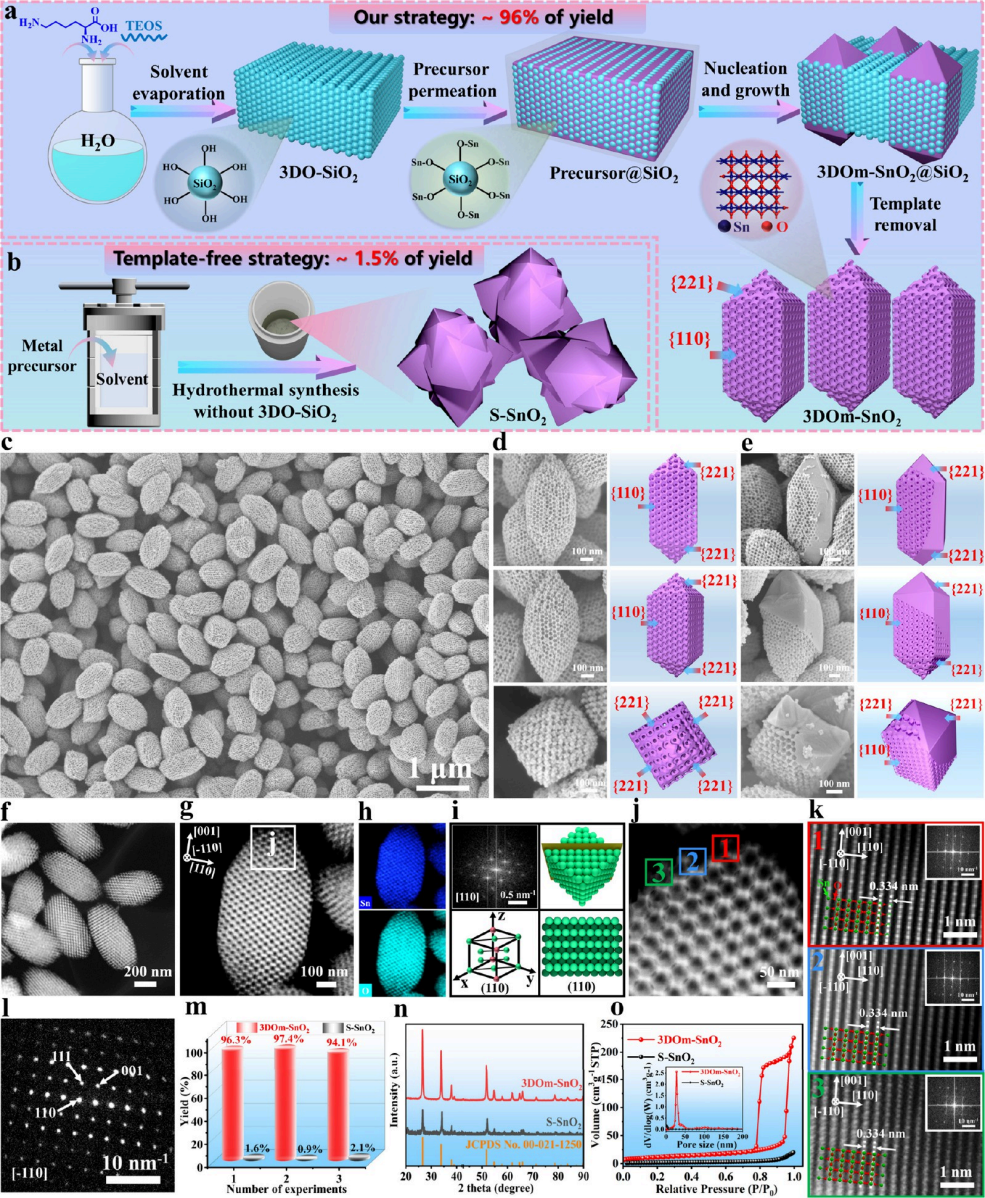
Synthesis and characterization of 3DOm-SnO_2_. Schematic illustration of the preparation procedures of (a) 3DOm-SnO_2_ and (b) S-SnO_2_. (c) Low-resolution SEM image of 3DOm-SnO_2_. (d) SEM images of individual 3DOm-SnO_2_ crystals from different directions and their corresponding schematic models. (e) SEM images of partly templated 3DOm-SnO_2_ crystals and their corresponding schematic models. (f, g) STEM images, (h) EDS mapping images, and (i) FFT pattern with the corresponding schematics of the mesopore arrangement of 3DOm-SnO_2_. (j) STEM image of the area indicated by the white square in (g). (k) Atomic-resolution STEM images of the three areas indicated by the red, blue, and green squares in (j). (l) SAED pattern of a whole 3DOm-SnO_2_ particle. (m) Yields of 3DOm-SnO_2_ and S-SnO_2_ in their three repeated synthetic experiments. (n) XRD patterns and (o) N_2_ adsorption/desorption isotherms of 3DOm-SnO_2_ and S-SnO_2_. The insets in (k) and (o) show the corresponding FFT patterns and pore distribution curve, respectively.

The preparation procedure of 3DOm-SnO_2_ is schematically illustrated in Figure 1a. First, 3D-ordered silica nanospheres (termed 3DO-SiO_2_) were successfully synthesized by hydrolyzing tetraethyl silicate (TEOS) in an aqueous solution of basic amino acid (l-lysine).^31^ Scanning electron microscopy (SEM) images (Figure S1) reveal the successful synthesis of highly ordered silica nanospheres with sizes of ∼27 nm, which were employed as a hard template for fabricating 3DOm-SnO_2_. In a subsequent hydrothermal process, the hydrophilic silanol groups (Si–OH) on 3DO-SiO_2_ could facilitate the pore filling and surface adsorption of Sn ions, leading to the spontaneous nucleation and in situ growth of 3DOm-SnO_2_ in the periodic voids of the 3DO-SiO_2_ mold (the resultant composite is denoted as 3DOm-SnO_2_@SiO_2_). The powder X-ray diffraction (PXRD) patterns in Figure S2 confirm the successful formation of crystalline SnO_2_ species in 3DO-SiO_2_ after the hydrothermal process. In addition, the SEM images of 3DOm-SnO_2_@SiO_2_ (Figure S3) reveal that almost all of the 3DOm-SnO_2_ particles had formed in the interior of 3DO-SiO_2_ rather than on its surface. Finally, the silica nanospheres could be readily removed from 3DOm-SnO_2_@SiO_2_ by a simple etching process, leaving behind highly uniform 3DOm-SnO_2_ in a single-crystalline form. For comparison, we also prepared a solid SnO_2_ counterpart (denoted as S-SnO_2_) by using the same synthetic procedure as 3DOm-SnO_2_, except 3DO-SiO_2_ was not added as a template (Figure 1b).

Low-resolution SEM images show that 3DOm-SnO_2_ has a uniform oval-shaped morphology with a uniform size of ∼480 nm in width and ∼840 nm in length (Figures 1c and S4). Impressively, the dodecahedral morphology with a 3D-ordered mesoporous nanoarchitecture could be identified by the representative SEM images of individual 3DOm-SnO_2_ particles taken from three different directions and their corresponding schematic models (Figure 1d). Fortunately, we also sought out three partly templated 3DOm-SnO_2_ particles, of which the untemplated parts show an unambiguous tetrakaidecahedron morphology with four {110} facets and eight {221} facets (Figures 1e and S5), directly suggesting the perfect single-crystalline nature of 3DOm-SnO_2_. The highly ordered mesopores with diameters of ∼27 nm can be clearly observed from the surface to internal center in 3DOm-SnO_2_ in the obtained transimission electron microsocpy (TEM) and dark-field scanning transmission electron microscopy (STEM) images (Figures 1f and S6). The good accessibility of 3DOm-SnO_2_ can be elucidated by the Cs-corrected STEM images and corresponding elemental mapping images of a selected particle, whose ordered mesopores correspond to the (110) planes in a face-centered cubic (fcc) arrangement with high interconnectivity (Figures 1g–i and S7). Impressively, the Cs-corrected STEM images (Figures 1k and S8) of three different positions taken from the same 3DOm-SnO_2_ particle (Figure 1g,j) show the same clear lattice fringe spacing of 0.334 nm, which is consistent with the (110) planes of tetragonal SnO_2_^32^ (Figure S9) and in line with the corresponding fast Fourier transform (FFT) patterns (the insets in Figure 1k) and above SEM observations (Figure 1c–e). Furthermore, the selected-area electron diffraction (SAED) pattern (Figure 1l) taken from a whole 3DOm-SnO_2_ particle displays ordered diffraction spots, from which we can infer that the crystal axis is along the [−110] direction. All of these observations reliably confirm the 3D-ordered mesopores and perfect single-crystalline feature of 3DOm-SnO_2_. In contrast, the S-SnO_2_ particles prepared in the absence of 3DO-SiO_2_ aggregated severely (Figure S10), showing a polycrystalline feature without identifiable facets (Figure S11). These results demonstrate that, compared to the template-free homogeneous crystallization of S-SnO_2_ in bulk solution, the confined heterogeneous crystallization of 3DOm-SnO_2_ in 3DO-SiO_2_ provided more precise control over its morphology, crystalline structure, surface regularity, porosity, and particle uniformity.^33,34^ More importantly, as shown in Figure 1m and Table S1, the average yield of 3DOm-SnO_2_ (on a feeding Sn basis) in three repeated synthetic experiments reached up to ∼96%, which is ∼64 times higher than that of S-SnO_2_ (∼1.5%), confirming that the crystallization efficiency of SnO_2_ is greatly improved by using our nanocasting strategy. We deduce that the silanol groups on 3DO-SiO_2_ can remarkably reduce the energy barrier of crystallization of 3DOm-SnO_2_ relative to the conventional homogeneous crystallization of S-SnO_2_ in bulk solution. The detailed role of silicon hydroxyls is explored in the mechanism study section. To our knowledge, this study represents the first demonstration that 3D-ordered silica nanospheres can be directly employed as a mold to synthesize 3DOm crystalline materials.

Subsequently, the crystalline structures and purity of various samples were examined by PXRD. As shown in Figure 1n, the XRD patterns of both 3DOm-SnO_2_ and S-SnO_2_ could be indexed to the rutile phase of tetragonal SnO_2_ (JCPDS No. 00-021-1250),^35^ indicating that the introduction of 3D-ordered mesopores does not change the crystalline structure of the resultant material, 3DOm-SnO_2_. However, 3DOm-SnO_2_ has a much higher degree of crystallinity than S-SnO_2_, as confirmed by its much higher diffraction peaks without any impurity. N_2_ adsorption/desorption experiments were further performed to compare the pore structures of 3DOm-SnO_2_ and S-SnO_2_. As shown in Figure 1o, 3DOm-SnO_2_ displays a type IV sorption isotherm with a high nitrogen uptake and an obvious hysteresis loop in the relative pressure (*P*/*P*_0_) range of 0.78–0.96, which reveal the presence of uniform mesopores in this sample.^36^ The corresponding pore size distribution curve suggests that the mesopore size of 3DOm-SnO_2_ is ∼27 nm at maximum distribution (Figure 1o, inset), which is in good agreement with the STEM observations (Figure S6). Expectedly, S-SnO_2_ showed a very low nitrogen uptake in the whole *P*/*P*_0_ range, signifying the absence of mesopores in this sample. Consequently, the total pore volume of 3DOm-SnO_2_ is up to 0.42 cm^3^/g, which is 56 times larger than that of S-SnO_2_ (0.0074 cm^3^/g) (Table S2). Furthermore, the high-resolution O 1s X-ray photoelectron spectroscopy (XPS) spectra reveal that 3DOm-SnO_2_ has a much higher O_ads_/(O_ads_ + O_latt_) ratio (64.57%) than S-SnO_2_ (47.51%), suggesting the formation of more oxygen vacancies in its 3DOm structure, which not only facilitate the dispersion and stabilization of active metal species by forming strong metal–support interactions but also provide abundant adsorption sites for reactants/intermediates (Figure S12 and Table S3).^37,38^ Additionally, the high-resolution Sn 3d XPS spectra (Figure S13) show two peaks at 495.8 and 487.2 eV, corresponding to Sn 3d_3/2_ and Sn 3d_5/2_ of the Sn^4+^ oxidation state, respectively, which are characteristic of SnO_2_.^39^

### Exploration of the Role of Silanol Groups in the Synthesis of 3DOm-SnO_2_

**Figure 2:**
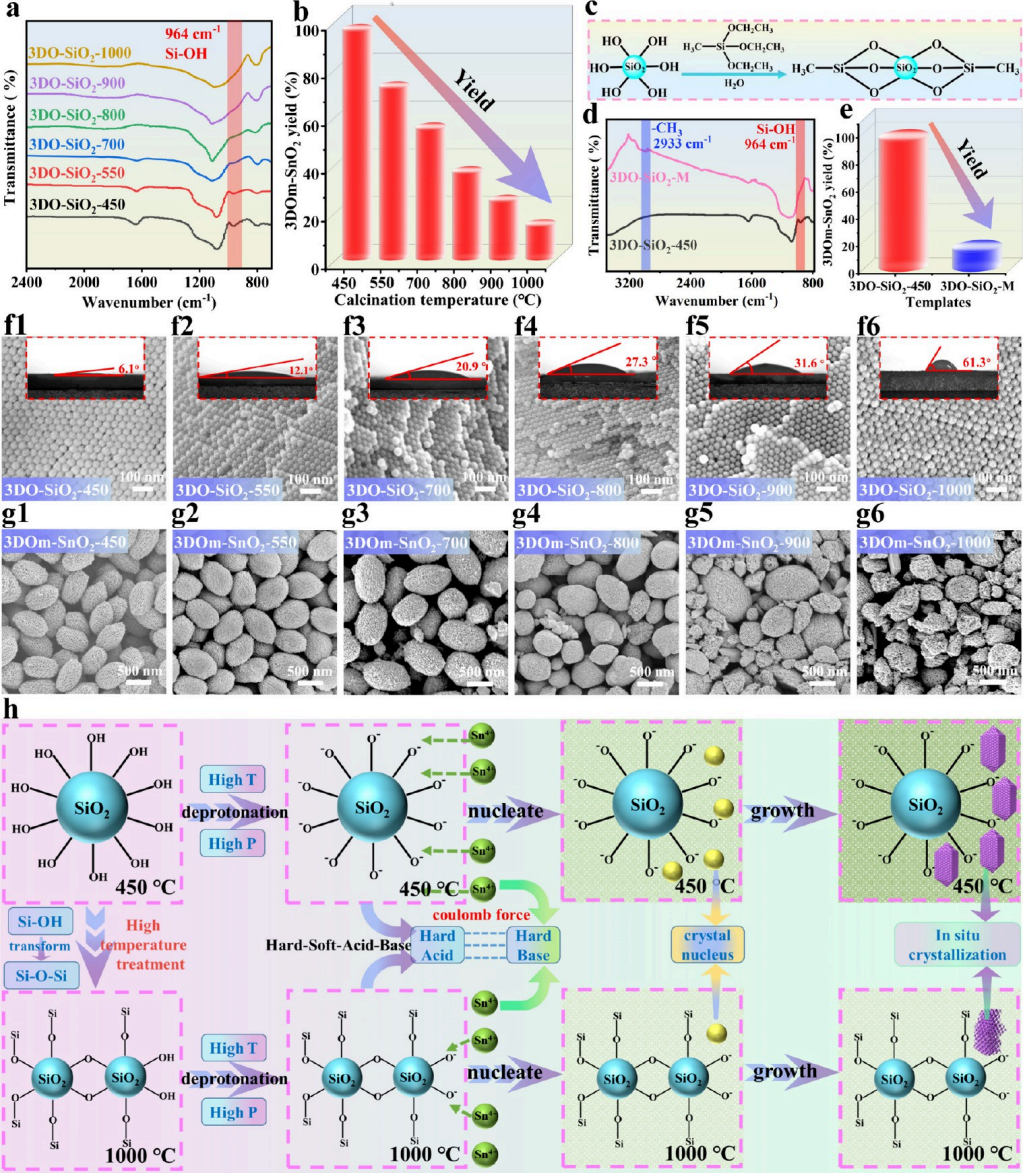
Formation mechanism studies of 3DOm-SnO_2_. (a) FTIR spectra of various templates. (b) Yield of 3DO-SnO_2_ as a function of the calcination temperature of 3DO-SiO_2_-*T*. (c) Surface modification mechanism and (d) FTIR spectra of 3DO-SiO_2_-M and 3DO-SiO_2_-450. (e) Yield of 3DOm-SnO_2_ over 3DO-SiO_2_-M and 3DO-SiO_2_-450 templates. (f1–f6) SEM images of 3DO-SiO_2_-*T* and their corresponding water contact angles. (g1–g6) SEM images of 3DOm-SnO_2_-*T*. (h) Schematic illustrations of the possible assistance mechanism of silanol groups in the synthesis of 3DOm-SnO_2_.

Considering that it was the employment of 3DO-SiO_2_ as a template that enabled the high-yield synthesis of 3DOm-SnO_2_, we further investigated the detailed role of 3DO-SiO_2_ in inducing the heterogeneous crystallization of 3DOm-SnO_2_ in its periodic voids. After the hydrothermal process, we found the separated solution from 3DOm-SnO_2_@SiO_2_ was still clear without any solid product, which revealed that SnO_2_ could not nucleate in the bulk solution; this is in good accordance with the growth of S-SnO_2_ being just on the wall of the employed hydrothermal reactor with a very low yield of ∼1.5% (Figure S14). Therefore, it was deduced that the silanol groups on 3DO-SiO_2_ may play a crucial role in the high-yield synthesis of 3DOm-SnO_2_. To confirm this, we precisely regulated the density of silanol groups on 3DO-SiO_2_ by changing the calcination temperature (denoted as 3DO-SiO_2_-*T*, where *T* represents the calcination temperature), as a high calcination temperature can accelerate the polycondensation of Si–OH groups to form Si–O–Si groups.^40^ As depicted in Figure 2a, the Fourier transform infrared (FTIR) spectrum of 3DO-SiO_2_-450 shows an obvious absorption peak at 964 cm^–1^, which can be assigned to the bending vibration of the Si–OH bond.^41^ However, with an increase in the calcination temperature, the Si–OH peak gradually decreases in intensity, indicating that the density of silanol groups on 3DO-SiO_2_-*T* decreases with the calcination temperature. Water contact angle tests further confirmed that 3DO-SiO_2_-450 exhibited the most hydrophilic property with the lowest contact angle of 6.1° due to having the richest surface silanol groups (Figure 2f, insets), which could benefit the pore filling of the precursor solution and promote the adsorption of Sn^4+^ on 3DO-SiO_2_-450 to induce the heterogeneous nucleation of 3DOm-SnO_2_. Subsequently, we employed these 3DO-SiO_2_-*T* materials as templates to explore the effect of the density of silanol groups on the synthesis of 3DOm-SnO_2_-*T*. Interestingly, as shown in Figure 2b, the yield of 3DOm-SnO_2_-*T* sharply decreased with the calcination temperature, and 3DOm-SnO_2_-450 (i.e., 3DOm-SnO_2_) showed the highest yield of ∼96%, which is 6.9 times higher than that of 3DO-SiO_2_-1000 (∼14%). Given that these 3DO-SiO_2_-*T* templates had the same morphology but different surface properties, the highest yield of 3DOm-SnO_2_-450 can be reasonably attributed to the richest Si–OH groups of its corresponding 3DO-SiO_2_-450 template, which can serve as active sites to capture Sn^4+^ and thus induce in situ nucleation and crystallization of 3DOm-SnO_2_-450. In addition, as shown in Figures 2f,g and S15–S20 and Table S4, the orderliness of 3DO-SiO_2_-*T* was hardly affected by the calcination temperature, but the uniformity and crystallinity of the resultant 3DOm-SnO_2_-*T* materials became increasingly poor with an increase in the calcination temperature, which is in good accordance with the variation trend of their yields. To further confirm the crucial role of silanol groups, we successfully removed the surface silanol groups of 3DO-SiO_2_ by the modification of triethoxy(ethyl)silane (Figure 2c), as proven by the disappearance of the Si–OH peak (964 cm^–1^) in the FTIR spectrum (Figure 2d). Then, the resultant material, 3DO-SiO_2_-M, was employed as a template to synthesize the corresponding 3DOm-SnO_2_-M. As expected, 3DOm-SnO_2_-M showed a very low yield of <15% with an indiscernible morphology and poor uniformity (Figures 2e and S21). All of these results reveal that the richest silanol groups on 3DO-SiO_2_-450 can induce the heterogeneous crystallization of SnO_2_ single crystals in its periodic voids, leading to the high-yield synthesis of 3DOm-SnO_2_-450.

Based on the above observations, we propose a plausible synthesis mechanism for 3DOm-SnO_2_. In a hydrothermal condition, the silanol groups on 3DO-SiO_2_ experience a deprotonation process to produce Si–O^–^ groups by following Le Châtelier’s principle^42^ (Figure S22). Then, the uncoordinated Si–O^–^ groups can capture Sn^4+^ ions by forming Si–O–Sn groups due to the Coulomb interaction between the Si–O^–^ groups (serving as a hard base) and Sn^4+^ ions (serving as a hard acid), based on the classical hard and soft acid and base theory.^43^ Subsequently, with the enrichment of Sn in 3DO-SiO_2_, SnO_2_ can spontaneously nucleate on the interior surface of 3DO-SiO_2_, thus initiating the heterogeneous crystallization of 3DOm-SnO_2_ in its periodic voids. It is, therefore, easy to understand why the yield of 3DOm-SnO_2_-450 could reach up to ∼96% but that of 3DOm-SnO_2_-1000 was only ∼14%, as clearly depicted in Figure 2h. To certify the possible interaction between Sn^4+^ and Si–OH, we hydrothermally treated 3DO-SiO_2_-450 and 3DO-SiO_2_-1000 in an aqueous solution of SnCl_4_·5H_2_O at 150 °C to avoid any crystallization of SnO_2_. Energy dispersive X-ray spectroscopy (EDS) mapping images and elemental analysis revealed that there were very few Sn ions on the treated 3DO-SiO_2_-1000 due to it having the fewest silanol groups (Figure S23). In sharp contrast, there was a large amount of Sn^4+^ ions on the treated 3DO-SiO_2_-450 (Figure S24). Expectedly, the FTIR spectrum of the treated 3DO-SiO_2_-450 confirms this, as the peak of Si–OH (964 cm^–1^) disappeared and a new peak appeared at 708 cm^–1^, which can be attributed to the stretching vibration of Sn–O bonds, revealing the strong interaction between Si–O^–^ and Sn^4+^ (Figures S25 and S26). These results support that there is an enrichment of Sn^4+^ on 3DO-SiO_2_ by the formation of Sn–O bonds in the hydrothermal process, which facilitates the heterogeneous crystallization of 3DOm-SnO_2_ with a high yield and good reproducibility.

### The Excellent Tunability of 3DOm-SnO_2_ in the Pore Size

**Figure 3:**
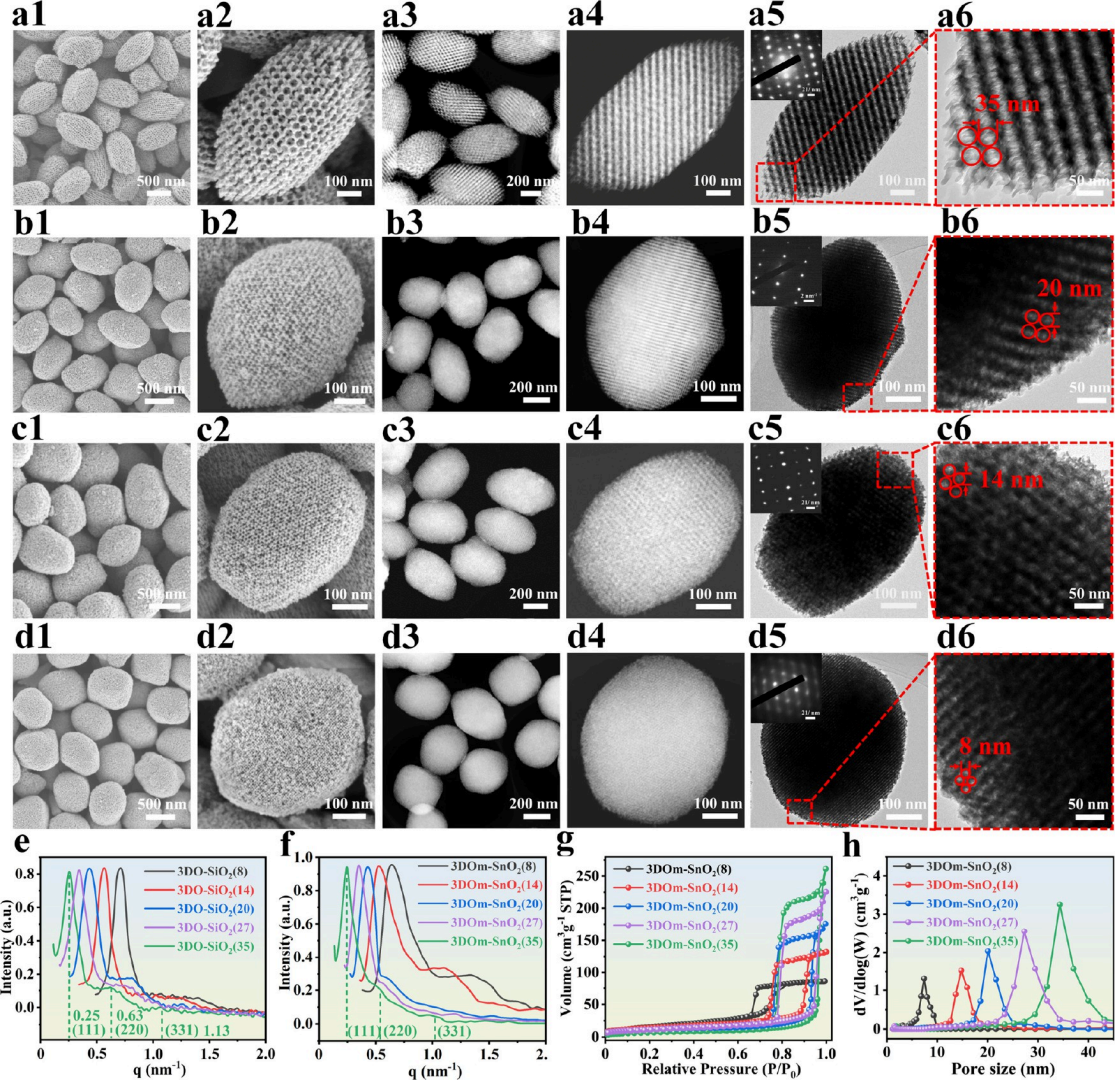
Excellent tunability of 3DOm-SnO_2_ in the pore size. (a1–d1, a2–d2) SEM, (a3–d3, a4–d4) STEM, and (a5–d5, a6–d6) TEM images of 3DOm-SnO_2_(35) (a1–a6), 3DOm-SnO_2_(20) (b1–b6), 3DOm-SnO_2_(14) (c1–c6), and 3DOm-SnO_2_(8) (d1–d6). (e) SAXS patterns of various 3DO-SiO_2_(*S*) templates. (f) SAXS patterns, (g) N_2_ adsorption/desorption isotherms, and (h) pore size distributions of various 3DOm-SnO_2_(*S*) samples. The insets in (a5–d5) show the corresponding SAED patterns.

To manifest the good applicability of this developed strategy, we successfully synthesized a series of 3DOm-SnO_2_(*S*) with mesopore sizes ranging from 8 to 35 nm by precisely controlling the diameter of the corresponding 3DO-SiO_2_(*S*) templates (*S* represents the average diameter of 3DO-SiO_2_). As shown in Figures 3a–d and S27–S42, all of the 3DOm-SnO_2_(*S*) samples displayed an oval-shaped morphology with uniform mesopores in a periodic fcc arrangement, which are in good accordance with the replication of ∼8, 14, 20, and 35 nm nanospheres of the 3DO-SiO_2_(*S*) templates with high precision. The SAED patterns, high-resolution TEM (HRTEM) images, FFT patterns, and XRD patterns (the insets of Figure 3a5–d5 and Figures S43–S44) firmly confirm the single-crystalline nature of 3DOm-SnO_2_(*S*) with the same rutile structure. The periodic mesopores of 3DOm-SnO_2_(*S*) imprinted from the 3DO-SiO_2_(*S*) templates could be further revealed by a small-angle X-ray scattering (SAXS) measurement. As shown in Figure 3e,f, each 3DOm-SnO_2_(*S*) sample displays a SAXS pattern that is very similar to that of its corresponding 3DO-SiO_2_(*S*) template, highlighting the perfect replication of the periodicity of 3DO-SiO_2_(*S*) by 3DOm-SnO_2_(*S*) with precise control. Furthermore, as the mesopore size of 3DOm-SnO_2_(*S*) decreases, the lowest angle peak of the (111) Bragg reflection shifts toward higher *q* values, which suggests a gradual decrease in the center-to-center distances between the periodic mesoporous elements.^44^ Similarly, adsorption/desorption isotherms and the corresponding pore size distributions also indicate that the average mesopore sizes of 3DOm-SnO_2_(8), 3DOm-SnO_2_(14), 3DOm-SnO_2_(20), and 3DOm-SnO_2_(35) were about 8, 14, 20, and 35 nm, respectively (Figure 3g,h), which are consistent with the HRTEM observations (Figure 3a6–d6). All of these results demonstrate the good versatility of our strategy for the construction of 3DOm single-crystalline architectures with previously unrealized tunability.

### Catalytic tests for the Sequential Hydrogenation of 4-Nitrophenylacetylene (NPA)

**Figure 4:**
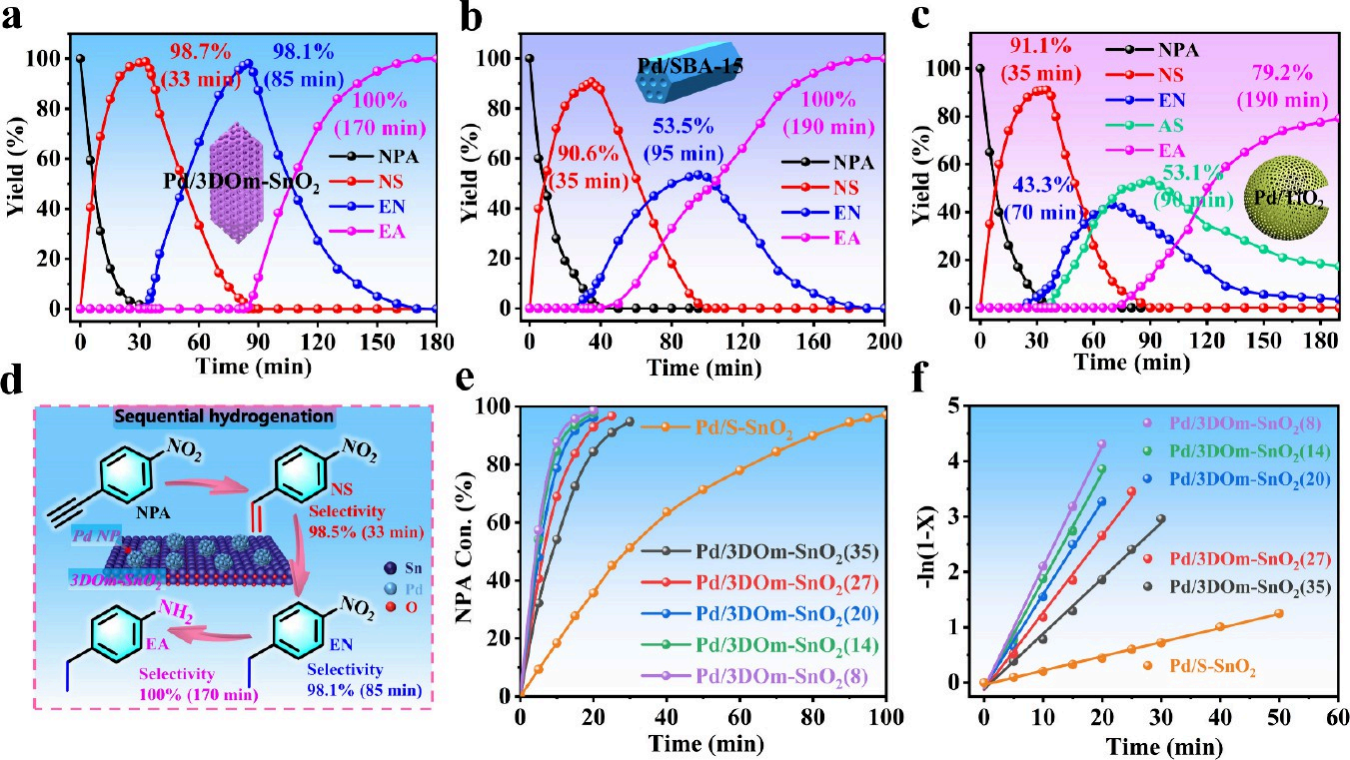
Catalytic tests for the sequential hydrogenation of NPA. Yields of various products as a function of reaction time on (a) Pd/3DOm-SnO_2_(27), (b) Pd/SBA-15, and (c) Pd/TiO_2_. (d) Sequential hydrogenation of NPA to form various products on Pd/3DOm-SnO_2_(27). (e) Conversion of NPA as a function of reaction time on various catalysts. (f) Relationship between ln(1 – *X*) and reaction time for various catalysts.

Benefiting from its fast mass transfer, large accessible surface, and favorable single-crystalline property, 3DOm-SnO_2_ is expected to be an excellent support for dispersing metal NPs. Thus, we anchored Pd NPs on 3DOm-SnO_2_(27) to prepare Pd/3DOm-SnO_2_(27) (Figures S45–S49 and Table S5). As shown in Figure S50, the Sn 3d XPS peaks of Pd/3DOm-SnO_2_(27) exhibit an obvious positive shift of ∼0.6 eV compared to those of 3DOm-SnO_2_(27), which indicates that electrons were transferred from SnO_2_ to the Pd NPs, resulting in the accumulation of electrons on the Pd NPs to bring about some particular catalytic properties.^45^ Thus, we further investigated the catalytic performance of Pd/3DOm-SnO_2_(27) for the hydrogenation of 4-nitrophenylacetylene (NPA) to various high-value-added products (Figure S51). Recently, a series of heterogeneous catalysts were designed for the selective hydrogenation of NPA.^46,47^ However, so far, achieving the sequential hydrogenation of NPA remains a great challenge for chemists since the hydrogenation behaviors of the −C=C, −C≡C, and −NO_2_ groups in NPA are very similar.^48,49^ Delightedly, as shown in Figure 4a and Table S6, Pd/3DOm-SnO_2_(27) was able to realize the sequential hydrogenation of NPA to selectively produce 4-nitrostyrene (NS), then 4-nitroethylbenzene (EN), and finally 4-aminoethylbenzene (EA). Particularly, the maximum selectivities of NS and EN could reach ∼98.7% and ∼98.1% at the reaction times of 33 and 85 min, respectively, which reveal that any significant hydrogenation of NS or EN only occurs once its previous hydrogenation reaction has been almost completed. To the best of our knowledge, Pd/3DOm-SnO_2_ may represent the first catalyst to achieve the three-step sequential hydrogenation of NPA (Table S8), which suggests that there exists a substrate inhibition effect for the second and third hydrogenation steps, as revealed by our poisoning experiments and theoretical calculations. As shown in Figures S52 and S53, the hydrogenation of NS and EN is almost fully poisoned in the presence of 1 mmol of NPA and 1 mmol of NS, respectively, which suggests the binding interaction of these substrates on Pd NPs is weakened in the order of NPA > NS > EN. Namely, NPA can be preferentially adsorbed on the Pd NPs of Pd/3DOm-SnO_2_(27) to impede the hydrogenation of NS, and only when the hydrogenation of NPA is basically complete, NS tends to adsorb on the Pd NPs for further hydrogenation. Similarly, when the hydrogenation of NS is basically complete to expose the Pd NPs, EN begins to adsorb on them for the subsequent reaction. Combining these findings with the aforementioned XPS results, we tentatively attribute the sequential hydrogenation of NPA over Pd/3DOm-SnO_2_ to the favorable electron transfer from the neutral SnO_2_ support to the Pd NPs, which positively modulates the adsorption free energies of NPA, NS, and EN on the Pd NPs to realize their sequential hydrogenation.

To confirm this, we also used mesoporous SBA-15 and TiO_2_ as supports to load Pd NPs (denoted as Pd/SBA-15 and Pd/TiO_2_, respectively; see Figures S54 and S55 and Table S5). The Pd 3d XPS spectra in Figure S56 indicate that the binding energies of Pd/SBA-15 and Pd/TiO_2_ show a positive shift of ∼0.3 eV and a negative shift of ∼0.2 eV compared to those of Pd/3DOm-SnO_2_(27), respectively. These results demonstrate that excessive electrons accumulate on the Pd NPs of Pd/TiO_2_, while insufficient electrons accumulate on the Pd NPs of Pd/SBA-15. As a result, the selectivity of NS for Pd/SBA-15 can reach a maximal value of only 90.6% at 35 min (Figures 4b and S57 and Table S6). Subsequently, as the reaction progresses, EN reaches a maximal selectivity of only 53.5% after 95 min. Similarly, Pd/TiO_2_ only gives a maximal selectivity of 91.1% for NS, and NS is converted to EN and 4-aminostyrene (AS) with maximal selectivities of only 43.3% and 53.1% at 70 and 90 min, respectively. Then, both EN and AS are further hydrogenated into EA with a maximal selectivity of 79.2% at 190 min (Figures 4c and S58 and Table S6). These results indicate that the hydrogenation of NPA over both catalysts is difficult to control, resulting in their poor sequential hydrogenation performances. Considering the different electronic properties of the above catalysts that support the same Pd species, we ascribe the good sequential hydrogenation performance of Pd/3DOm-SnO_2_(27) (Figure 4d) to the positive synergistic effect between the Pd NPs and 3DOm-SnO_2_ for this reaction.

Subsequently, we further investigated the influence of the mesopore size of Pd/3DOm-SnO_2_(*S*) on the catalytic activity. Note that NS is an industrially important intermediate for many fine chemicals and petrochemicals.^50^ Thus, the hydrogenation of NPA to NS was employed as a model reaction to obtain the detailed size–performance relationship of Pd/3DOm-SnO_2_(*S*) (Figures S59–S62). As shown in Figure 4e, the time to complete the conversion of NPA gradually increased as the mesopore size of Pd/3DOm-SnO_2_(*S*) increased. Impressively, all of the Pd/3DOm-SnO_2_(*S*) materials showed a good linear relationship between ln(1 – *X*) (*X* represents the conversion of NPA) and the reaction time (Figure 4f and Table S7), and Pd/3DOm-SnO_2_(8) with the smallest mesopores showed the fastest first-order reaction kinetics, which can be attributed to it have the highest surface area with the richest active sites. In addition, Pd-supported S-SnO_2_ (denoted as Pd/S-SnO_2_) was also prepared and employed as a catalyst for this reaction (Figure S63 and Table S5). Expectedly, the time to complete the conversion of NPA increased sharply to 100 min for Pd/S-SnO_2_ (Figure 4e) due to the absence of favorably ordered mesopores in its structure. Additionally, benefiting from its highly robust single-crystalline framework, Pd/3DOm-SnO_2_(27) exhibited excellent catalytic stability, as revealed by its nearly unchanged activity after the 10th run (Figure S64). A hot filtration experiment demonstrated that there were no more increments in the conversion of NPA after the catalyst was removed from the reaction solution, suggesting the heterogeneous nature of our catalytic system (Figure S65). XPS results further showed that the Pd 3d binding energies of the reused Pd/3DOm-SnO_2_(27) were lower by ∼0.2 eV than those of the fresh one (Figure S66), which may be caused by the slight aggregation of Pd NPs after 10 runs, as revealed by TEM images (Figure S67). Subsequently, detailed characterizations confirmed that the 3DOm structure and the crystallinity of the used catalyst were basically the same as those of the fresh one (Figure S67 and S68). Its desirable sequential hydrogenation performance together with its good recyclability and superior activity make Pd/3DOm-SnO_2_ a good potential material for practical applications in catalysis.

### Density Functional Theory (DFT) Calculations

**Figure 5:**
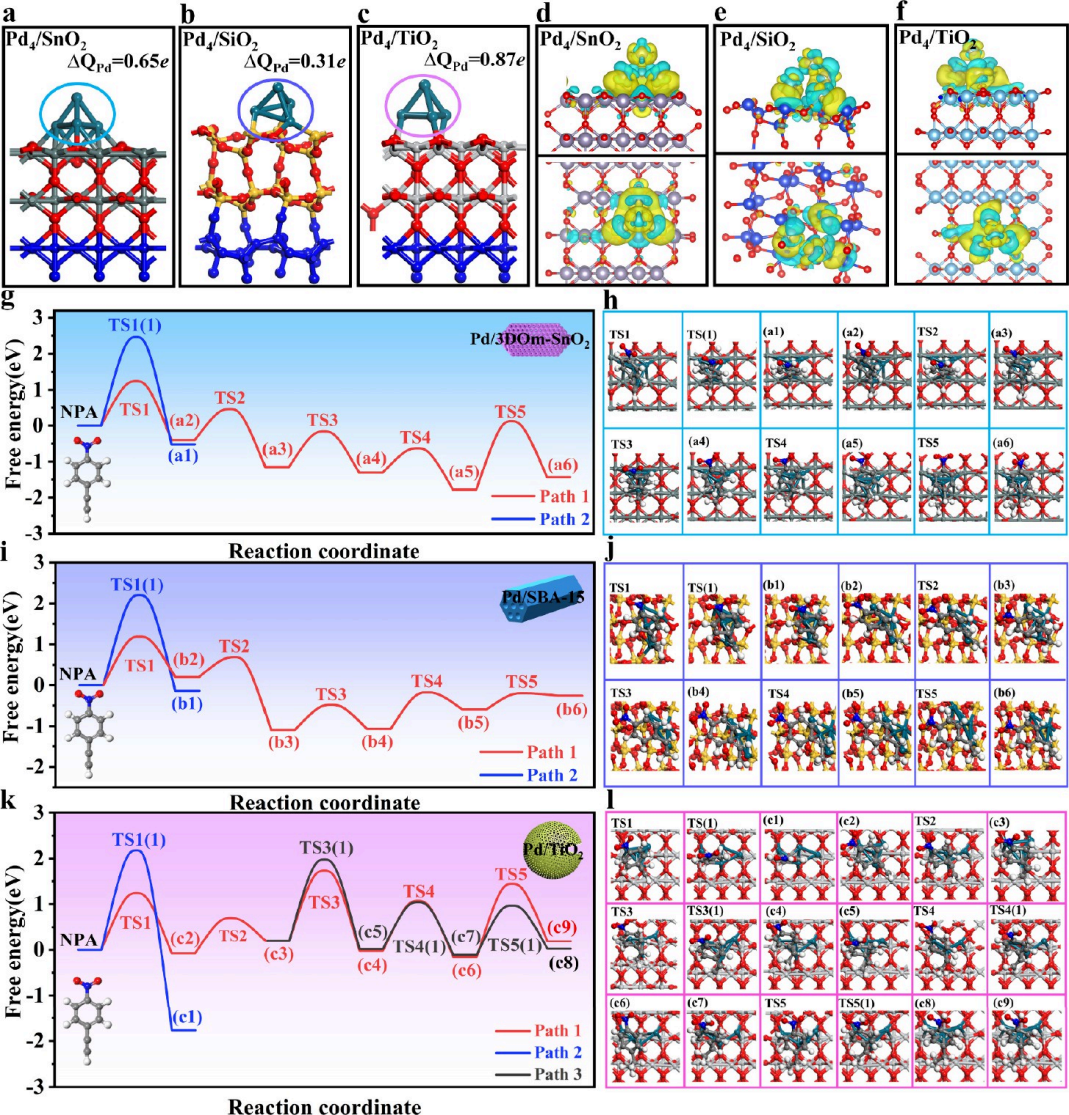
DFT calculations. Configurations (side view) and Bader charge of (a) Pd_4_/SnO_2_, (b) Pd_4_/SiO_2_, and (c) Pd_4_/TiO_2_. Charge difference plots (side view (top) and top view (down)) of (d) Pd_4_/SnO_2_, (e) Pd_4_/SiO_2_, and (f) Pd_4_/TiO_2_. Free energy diagrams for the hydrogenation of NPA on (a) Pd_4_/SnO_2_, (i) Pd_4_/SiO_2_, and (k) Pd_4_/TiO_2_. Simplified surface structures of various reaction species along the reaction pathway on (h) Pd_4_/SnO_2_, (j) Pd_4_/SiO_2_, and (l) Pd_4_/TiO_2_. Pd (cyan), Sn (gray), Si (yellow), Ti (light gray), O (red), N (blue), C (gray), and H (white). “TS” denotes a transition state.

Bearing the above experimental results in mind, DFT calculations were further performed on Pd_4_/SnO_2_(110), Pd_4_/TiO_2_(110), and Pd_4_/SiO_2_(101) surface structures. As shown in Figures 5a–c and S69–S71, the Pd_4_ cluster in Pd_4_/SiO_2_(101) and Pd_4_/TiO_2_(110) is located on the ring skeleton and the Ti atom with a coordination number of six, respectively, while that in Pd_4_/SnO_2_(110) is located on the O atom of SnO_2_. Since Pd_4_ clusters can serve as active sites for the selective hydrogenation of NPA, we consider that the geometry structures of Pd_4_/SnO_2_(110), Pd_4_/TiO_2_(110), and Pd_4_/SiO_2_(101) are a very important factor that affect their catalytic performances. In addition, the Bader charge and differential charge densities of Pd_4_/SnO_2_(110), Pd_4_/SiO_2_(101), and Pd_4_/TiO_2_(110) were calculated and are shown in Figure 5a–f. The results show that electrons are transferred from SnO_2_ to the Pd_4_ cluster on Pd_4_/SnO_2_(110), which is also in accordance with the XPS results (Figure S49 and S50). Similar electron transfer also occurs on both Pd_4_/SiO_2_ and Pd_4_/TiO_2_. However, the numbers of electrons obtained by the Pd_4_ clusters of Pd_4_/SnO_2_(110), Pd_4_/SiO_2_(101), and Pd_4_/TiO_2_(110) were calculated to be 0.65, 0.31 and 0.87, respectively, suggesting that Pd_4_/SnO_2_(110) has a moderate amount of electron transfer between the Pd_4_ cluster and SnO_2_. Expectedly, the adsorption free energies (eV) of NPA, NS, and EN on Pd_4_/SnO_2_(110) follow the order of NPA(−1.03 eV) > NS(−0.91 eV) > EN(−0.80 eV), which is in good accordance with the hydrogenation sequence of these three substrates on Pd/3DOm-SnO_2_, while those on Pd_4_/SiO_2_(101) and Pd_4_/TiO_2_(110) do not follow this sequence (Figures S72–S74). These results reveal that the favorable synergistic effect between Pd NPs and SnO_2_ via moderate electron transfer can optimize the adsorption free energies of NPA, NS, and EN on Pd NPs, thus achieving their sequential hydrogenation on Pd/3DOm-SnO_2_.

Subsequently, we further calculated the energy barrier diagrams of different reaction paths for the hydrogenation of NPA on various catalysts, and the results are shown in Figure 5g–l and Tables S9–S11. The energy barriers of paths 1 and 2 on Pd_4_/SnO_2_(110), Pd_4_/SiO_2_(101), and Pd_4_/TiO_2_(110) are 2.46 eV versus 1.24 eV, 2.21 eV versus 1.19 eV and 2.18 versus 1.24 eV, respectively, which reveal that the hydrogenation of alkynyl groups is kinetically favorable by following path 1 rather than path 2 on the three catalysts. However, different following steps occur on the three catalysts to form different target products. For Pd_4_/SnO_2_(110), NPA is adsorbed and hydrogenated on its Pd_4_ cluster prior to NS since the adsorption free energy of NPA (−1.03 eV) is higher than that of NS (−0.91 eV), leading to the high selectivity of NS on Pd/3DOm-SnO_2_. Subsequently, the generated NS undergoes two exothermic reactions to easily form EN by overcoming the low energy barriers of 0.99 and 0.68 eV. Then, EN is slowly hydrogenated into *Ph(CH_3_CH_2_)NO_2_H (a6) via an endothermic process through TS-5 (this is the first step from EN to EA in the calculation), as its energy barrier is as high as 1.90 eV relative to those of Pd_4_/SiO_2_(110) (0.40 eV) and Pd_4_/TiO_2_(110) (1.6 eV), which is beneficial for realizing the high selectivity of EN on Pd/3DOm-SnO_2_. However, for Pd_4_/SiO_2_(110), the adsorption free energies of NPA (1.60 eV) and NS (1.64 eV) are comparable, and thus, the two substrates can be competitively adsorbed and hydrogenated on its Pd_4_ cluster, resulting in the low selectivity of NS. Afterward, the EN generated by the hydrogenation of NS can be efficiently converted into EA by overcoming an extremely low energy barrier of 0.40 eV, which reduces the selectivity of EN. Interestingly, for Pd_4_/TiO_2_(110), the generated NS from NPA can be hydrogenated to both EN and AS via paths 1 and 3 due to their similar energy barriers of 1.78 and 1.58 eV, respectively (Figure 4c). Finally, both EN and AS are slowly hydrogenated to produce EA by overcoming the high energy barriers of 1.60 and 1.07 eV, respectively. All these calculations confirm the unique sequential hydrogenation of NPA on Pd_4_/SnO_2_(110), which is in perfect agreement with our experimental results.

## Conclusions

In summary, we have developed a novel silanol-assisted nanocasting strategy to construct 3DOm-SnO_2_ with tunable pore size and well-defined facets. Our detailed mechanism studies showed that the silanol groups on 3DO-SiO_2_ can induce the heterogeneous crystallization of uniform 3DOm-SnO_2_ single crystals in its mesoscopic periodic avoidance by following the hard and soft acid and base theory. Impressively, the yield of the obtained 3DOm-SnO_2_ is as high as ∼96%, which is much superior to that of its solid counterpart prepared in the absence of 3DO-SiO_2_ (∼1.5%). By exploiting the favorable synergistic effect between Pd NPs and SnO_2_, we demonstrated that Pd/3DOm-SnO_2_ can realize the sequential catalytic hydrogenation of NPA to produce NS, then EN, and finally EA. DFT calculations and controlled experiments further demonstrated that the moderate electron transfer from Pd NPs to SnO_2_ can optimize the adsorption free energies of the above substrates to realize their sequential hydrogenation. This study not only paves the way for the nanofabrication of 3DOm single-crystalline materials with previously unrealized mesopore tunability by integrating the hydrothermal technique with the nanocasting strategy but also provides a new perspective for preparing sequential hydrogenation catalysts.

## Supporting Information Available

Supporting materials and methods, additional characterizations (SEM, TEM, HRTEM, AC-STEM, and EDS mapping images, XRD patterns, contact angles of water, FTIR spectra, N_2_ adsorption–desorption isotherms, pore size distributions, and XPS spectra), control experiments, catalytic results of obtained catalysts, and reaction mechanism (PDF)
