# Supplementary material for: Silanol-Assisted High-Yield Nanofabrication of SnO2 Single Crystals with Highly Tunable and Ordered Mesoporosity
Source: ACS Cent Sci. 2024 Jan 29;10(2):374–84. doi: 10.1021/acscentsci.3c01374 (PMC10906242; doi:10.1021/acscentsci.3c01374)
Supplement: Supplementary file 1 — oc3c01374_si_001.pdf [file oc3c01374_si_001.pdf]

Supporting Information

Silanol-assisted high-yield nanofabrication of SnO_2 single crystals with highly tunable and ordered mesoporosity

Shoukang Xiao,[+]† Li Wang,[+]† Ze Qin,† Xiao Chen,‡ Liyu Chen,† Yingwei Li,† and Kui Shen*†

†Guangdong Provincial Key Laboratory of Fuel Cell Technology, School of Chemistry and Chemical Engineering, South China University of Technology, Guangzhou, Guangdong 510640, China.

‡Beijing Key Laboratory of Green Chemical Reaction Engineering and Technology, Department of Chemical Engineering, Tsinghua University, Beijing 100084, China.

*Corresponding author.

E-mail: cekshen@scut.edu.cn

[+]These authors contributed equally to this work.

Table of Contents

Supporting Materials and Methods S6

Chemicals..S6

Preparation of 3D ordered silica nanospheres (namely 3DO-SiO_2(S), where S represents the average particle size of silica nanospheres)..S6

Preparation of 3D ordered mesoporous SnO_2 single crystals (namely 3DOm-SnO_2(S), where S represents the average pore size of 3DOm-SnO_2)..S7

Preparation of Pd-supported 3DOm-SnO_2(S) with the Pd loading of 1 wt% (namely Pd/3DOm-SnO_2(S))..S8

Preparation of Pd-supported TiO_2, SBA-15 and S-SnO_2 with the same Pd loading of 1 wt% (namely Pd/TiO_2, Pd/SBA-15 and Pd/S-SnO_2, respectively)..S8

Preparation of modified 3DO-SiO_2 by triethoxy(ethyl)silane (namely 3DO-SiO_2-M)..S9

The control experiments to explore of the role of silanol groups in the synthesis of 3DOm-SnO_2..S9

Characterizations..S10

DFT calculations..S11

Catalytic testing..S12

Supporting Figures S13

Figure S1...S13

Figure S2...S14

Figure S3...S15

Figure S4...S16

Figure S5...S17

Figure S6...S18

Figure S7...S19

Figure S8...S20

Figure S9...S21

Figure S10...S22

Figure S11. ....S23
Figure S12. ....S24
Figure S13. ....S25
Figure S14. ....S26
Figure S15. ....S27
Figure S16. ....S28
Figure S17. ....S29
Figure S18. ....S30
Figure S19. ....S31
Figure S20. ....S32
Figure S21. ....S33
Figure S22. ....S34
Figure S23. ....S35
Figure S24. ....S36
Figure S25. ....S37
Figure S26. ....S38
Figure S27. ....S39
Figure S28. ....S40
Figure S29. ....S41
Figure S30. ....S42
Figure S31. ....S43
Figure S32. ....S44
Figure S33. ....S45
Figure S34. ....S46
Figure S35. ....S47
Figure S36. ....S48
Figure S37. ....S49
Figure S38. ....S50
Figure S39. ....S51
Figure S40. ....S52

Figure S41. .... S53
Figure S42. .... S54
Figure S43. .... S55
Figure S44. .... S56
Figure S45. .... S57
Figure S46. .... S58
Figure S47. .... S59
Figure S48. .... S60
Figure S49. .... S61
Figure S50. .... S62
Figure S51. .... S63
Figure S52. .... S64
Figure S53. .... S65
Figure S54. .... S66
Figure S55. .... S67
Figure S56. .... S68
Figure S57. .... S69
Figure S58. .... S70
Figure S59. .... S71
Figure S60. .... S72
Figure S61. .... S73
Figure S62. .... S74
Figure S63. .... S75
Figure S64. .... S76
Figure S65. .... S77
Figure S66. .... S78
Figure S67. .... S79
Figure S68. .... S80
Figure S69. .... S81
Figure S70. .... S82

Figure S71. .... S83

Figure S72. .... S84

Figure S73. .... S85

Figure S74. .... S86

Supporting Tables S87

Table S1. .... S87

Table S2. .... S88

Table S3. .... S89

Table S4. .... S90

Table S5. .... S91

Table S6. .... S92

Table S7. .... S93

Table S8. .... S94

Table S9. .... S95

Table S10. .... S96

Table S11. .... S97

Supporting References S98

Supporting Materials and Methods

Chemicals

All chemicals with analytical grade are purchased from commercial suppliers and used without any further treatment. Tin chloride pentahydrate ($SnCl_4·5H_2O$, 99%, Aladdin Industrial Corporation), Palladium nitrate dihydrate ($Pd(NO_3)_2·2H_2O$, ≥99%, Aladdin Industrial Corporation), Tetrabutyl titanate (TBOT, 99%, Aladdin Industrial Corporation), Pluronic F127 (PEO_{106}-PPO_{70}-PEO_{106}, M_w = 12600 $g·mol^{-1}$, Aladdin Industrial Corporation), Acetic acid (AcOH, ≥99%, Aladdin Industrial Corporation) Tetraethyl orthosilicate (TEOS, 98%, Aladdin Industrial Corporation), L-lysine ($C_6H_{14}N_2O_2$, 98%, Aladdin Industrial Corporation), Triethoxy(ethyl)silane ($C_8H_{20}O_3Si$, 95%, Aladdin Industrial Corporation), Hydrochloric (HCl, 37%, Guangzhou Chem. Reagent Co., Ltd), Tetrahydrofuran (THF, 99%, Aladdin Industrial Corporation), Ethanol (C_2H_6O, 99%, Guangdong Guanghua Sci-Tech Co., Ltd), Pluronic P123 (PEO_{20}-PPO_{70}-PEO_{20}, 99%, Aladdin Industrial Corporation), N-butanol ($C_4H_{10}O$, 99%, Aladdin Industrial Corporation), 1-Ethynyl-4-nitrobenzene ($C_8H_5NO_2$, 97%, Aladdin Industrial Corporation), 1,4-dioxane ($C_4H_8O_2$, ≥99%, Aladdin Industrial Corporation).

Preparation of 3D ordered silica nanospheres (namely 3DO-SiO_2(S), where S represents the average particle size of silica nanospheres)

3D ordered silica nanospheres were prepared by hydrolyzing tetraethyl silicate (TEOS) in an aqueous solution of basic amino acid (L-lysine) based on a previously reported method with some modifications[1]. In general, 0.24 g L-lysine was dissolved

in 210 g deionized water, which was stirred for 10 min in a flat bottom flask. Next, 15.1 g TEOS was added to the above flask, and the resultant mixture was heated at 60 °C under vigorous stirring for 48 h. Subsequently, the 3DO-SiO_2(8) template with an average particle size of 8 nm was formed by slowly evaporating water out of the silica colloidal solution in an oven held at 70 °C for 24 h. The 3DO-SiO_2(14) template with an average particle size of 14 nm was prepared by the same synthetic procedure as 3DO-SiO_2(8) except that the 60 °C was replaced with 90 °C.

The 3DO-SiO_2 templates with average particle sizes of 20 nm, 27 nm and 35 nm were synthesized using a seeded growth method by using 14 nm silica nanospheres as seeds. Firstly, the sol containing 14 nm silica nanospheres was hydrothermal aged at 100 °C for 24 h to form a silica colloidal solution. Subsequently, the sol containing 20 nm silica nanospheres was prepared by supplying 15.1 g of TEOS into the above silica colloidal solution over 24 h. Similarly, the sol containing 27 or 35 nm silica nanospheres was prepared by supplying 15.1 g of TEOS into the above silica colloidal solution (14 nm) two times over 48 h or three times over 72 h, respectively. Then, the 3DO-SiO_2(20), 3DO-SiO_2(27) and 3DO-SiO_2(35) templates were formed by slowly evaporating water out of their corresponding silica colloidal solutions in an oven held at 70 °C for 24 h. Finally, the resulting 3DO-SiO_2(S) templates were calcined at 450 °C under air for 48 h to remove the L-lysine adsorbed on the silica nanospheres.

Preparation of 3D ordered mesoporous SnO_2 single crystals (namely 3DOm-SnO_2(S), where S represents the average pore size of 3DOm-SnO_2)

The 3DOm-SnO_2-S samples were prepared by hydrothermally treating Sn

precursor in the presence of the corresponding 3DO-SiO_2(S) templates. In a typical synthesis for 3DOm-SnO_2(27), 10 mL deionized water and 10 mL ethanol were added into a 100 mL autoclave, and then 0.673 g $SnCl_4{\cdot}5H_2O$ was added into the above solution. After the mixture was ultrasonicated for 10 min, 1.8 mL 37% HCl was added to the above mixture, which was dispersed by ultrasonic treatment for 10 min. Then 2 g of 3DO-SiO_2(27) was added and the sealed vessel was heated at 200 ℃ for 12 h, the heating rate is 2-3 °C per minute. Subsequently, the 3DO-SiO_2(27) templates were etched with 2 M NaOH for 24 h, and the resultant sample was harvested by centrifugation and washed successively with deionized water and ethanol. Finally, the product was dried overnight at 100 ℃ under vacuum for further use. In addition, other 3DOm-SnO_2(S) samples were also prepared by using the same synthetic procedure as 3DOm-SnO_2(27) except using the corresponding 3DO-SiO_2(S) templates.

Preparation of Pd-supported 3DOm-SnO_2(S) with the Pd loading of 1 wt% (namely Pd/3DOm-SnO_2(S))

At first, 100 mg 3DOm-SnO_2(S) was impregnated by dropwise addition of 100 µl aqueous solution containing 2.5 mg $Pd(NO_3)_2{\cdot}2H_2O$. After being subjected to ultrasonication for 1 h, the slurry was then dried by freeze-drying. Finally, the Pd^{2+} in as-synthesized samples was reduced to Pd^0 in a stream of H_2 at 300 °C for 2 h to obtain the corresponding Pd/3DOm-SnO_2(S).

Preparation of Pd-supported TiO_2, SBA-15 and S-SnO_2 with the same Pd loading of 1 wt% (namely Pd/TiO_2, Pd/SBA-15 and Pd/S-SnO_2, respectively)

TiO_2 and SBA-15 were first prepared according to the previous reports[2, 3]. For

comparison, the S-SnO_2 was also prepared by the same synthetic procedures as 3DOm-SnO_2 except without adding 3DO-SiO_2 as a template. The final Pd/TiO_2, Pd/SBA-15 and Pd/S-SnO_2 were prepared via the same procedure as the preparation of Pd/3DOm-SnO_2(S) except using different supports.

Preparation of modified 3DO-SiO_2 by triethoxy(ethyl)silane (namely 3DO-SiO_2-M)

The modification of 3DO-SiO_2 by using triethoxy(ethyl)silane to prepare 3DO-SiO_2-M was performed by following a previously reported procedure with some modifications[4, 5]. Typically, 0.5 g of triethoxy(ethyl)silane was dissolved in 20 mL mixture solution of ethanol and water (v : v = 1:1) and the resultant mixed solution was further stirred for 0.5 h to make it fully hydrolyzed. Subsequently, 1 g of 3DO-SiO_2 was immersed into the above solution for 1 h, and then further treated with vacuum degassing for 10 min to make all interstitial spaces between 3DO-SiO_2 filled with above solution. After reacting at 70 ℃ in N_2 atmosphere for 8 h, the obtained sample was washed with deionized water and ethanol, and dried under vacuum at 60 ℃ for 24 h to obtain the final 3DO-SiO_2-M. The modification principle of 3DO-SiO_2 is clearly shown in Figure 3c.

The control experiments to explore of the role of silanol groups in the synthesis of 3DOm-SnO_2

A series of controlled experiments were conducted to exploring the role of silanol groups in the synthesis of 3DOm-SnO_2. First, we have precisely regulated the density of silanol groups on 3DO-SiO_2 by changing its calcination temperature, since

higher calcination temperature always results in lower density of silanol groups on SiO_2 surface. The obtained templates are denoted as 3DO-SiO_2-T, where T represents the calcination temperature. Six templates can be obtained, which are 3DO-SiO_2-450, 3DO-SiO_2-550, 3DO-SiO_2-700, 3DO-SiO_2-800, 3DO-SiO_2-900 and 3DO-SiO_2-1000. Subsequently, we employed these 3DO-SiO_2-T templates to prepare the corresponding 3DOm-SnO_2-T by using the same hydrothermal conditions as the preparation of 3DOm-SnO_2 except that the template was replaced by 3DO-SiO_2-T. The results reveal that the yield of 3DOm-SnO_2-T sharply decreases with the calcination temperature. Similarly, we also employed 3DO-SiO_2-M was as a template to synthesize the corresponding 3DOm-SnO_2-M. As expected, 3DOm-SnO_2-M shows a very low yield of below 15%. These results reveal that the rich silanol groups on 3DO-SiO_2-450 can induce the heterogeneous crystallization of SnO_2 single crystals in its periodic voids, leading to the high-yield synthesis of 3DOm-SnO_2-450.

Meanwhile, to certify the possible interaction between Sn^{4+} and Si-OH, we hydrothermally treated 3DO-SiO_2-450 and 3DO-SiO_2-1000 in an aqueous solution of $SnCl_4 \cdot 5H_2O$ under 150 ℃ to avoid any crystallization of SnO_2 with other steps identical to those for the fabrication of 3DOm-SnO_2(S). Subsequently, we used FTIR and SEM-EDS to explore the existence form and concentration of Sn element on the template surface, respectively.

Characterizations

Powder X-ray diffraction patterns were collected by D8-Advance Bruker with Cu Kα radiation (40 kV, 40 mA, λ = 1.5406 Å). The BET surface areas and pore size

measurements were gained from N_2 adsorption/desorption isotherms at 77 K using a Micromeritics ASAP 2020 M instrument, and the corresponding pore size distribution curves of various samples were obtained on the basis of the DFT model by using the desorption branch of the isotherms. Before the analysis, the samples were degassed at 150 °C for 12 h. The morphology and elemental distributions of samples were determined using a transmission electron microscopy (TEM, JEOL, JEM-2100F) with an EDX analysis system (Bruker Xflash 5030T) at 200 kV. The surface morphology of samples was characterized by a high-resolution field-emission scanning electron microscopy (FESEM, HITACHI SU8220). The XPS spectra were obtained on a AXIS SUPRA+ instrument with an Al Kα source as radiation. The hydrophilicity of the samples was determined by contact angle meter (JC2000D3P, Shanghai, China). Thermo Fisher iS10 was utilized for the Fourier transform infrared spectroscopy (FTIR) experiments. The periodic mesoporous structures and size-scaling laws of solid samples were measured by small-angle X-ray scattering (SAXSess, Anton-Paar, Graz, Austria) with Cu Kα radiation (0.1542 nm wavelength) at 40 kV, 50 mA.

DFT calculations

DFT calculations were implemented using the Vienna Ab-initio Simulation Package (VASP)[6, 7]. The generalized gradient approximation with Perdew-Burke-Ernzerhof exchange-correlation functional (GGA-PBE) was adopted[8, 9]. Weak van der Waals interaction is considered by the DFT-D3 functional. Transition state was obtained using the climbing-image nudged elastic band method (CI-NEB). Transition state was identified by the number of imaginary frequencies (NIMG) with NIMG=1.

A cutoff energy was set to 400 eV. The structure optimization was converged until the force tolerance and energy difference were lower than 0.03 eV/Å and 10^{-5} eV, respectively.

The Gibbs free energy is calculated by

G=E+E_{ZPE}-TS

Where E, E_{ZPE}, and TS are total energy, zero-point energy and entropy of the system. In this study, the activation free energy (G_a) and reaction free energy (ΔG) of elementary reactions were calculated at 30 ℃.

Catalytic testing

All experiments were performed in a 20 mL Schlenk tube under magnetic stir. Typically, 1 mmol NPA was added to the Schlenk tube with 0.25 mol% catalyst (based on Pd content) and 4 mL 1,4-dioxane. Then the tube was vacuumed and purged with H_2 by a balloon (1 atm). The reaction mixture was stirred at room temperature. After reaction for a desired time, the catalyst was recycled by centrifugation, washed with ethanol and dried for next run. The supernatant was analyzed by GC-MS (Agilent, 7890B GC/5977A MS) equipped with a DB-35 MS UI capillary column (0.25 mm × 30 m) to determine the conversion and selectivity.

Supporting Figures

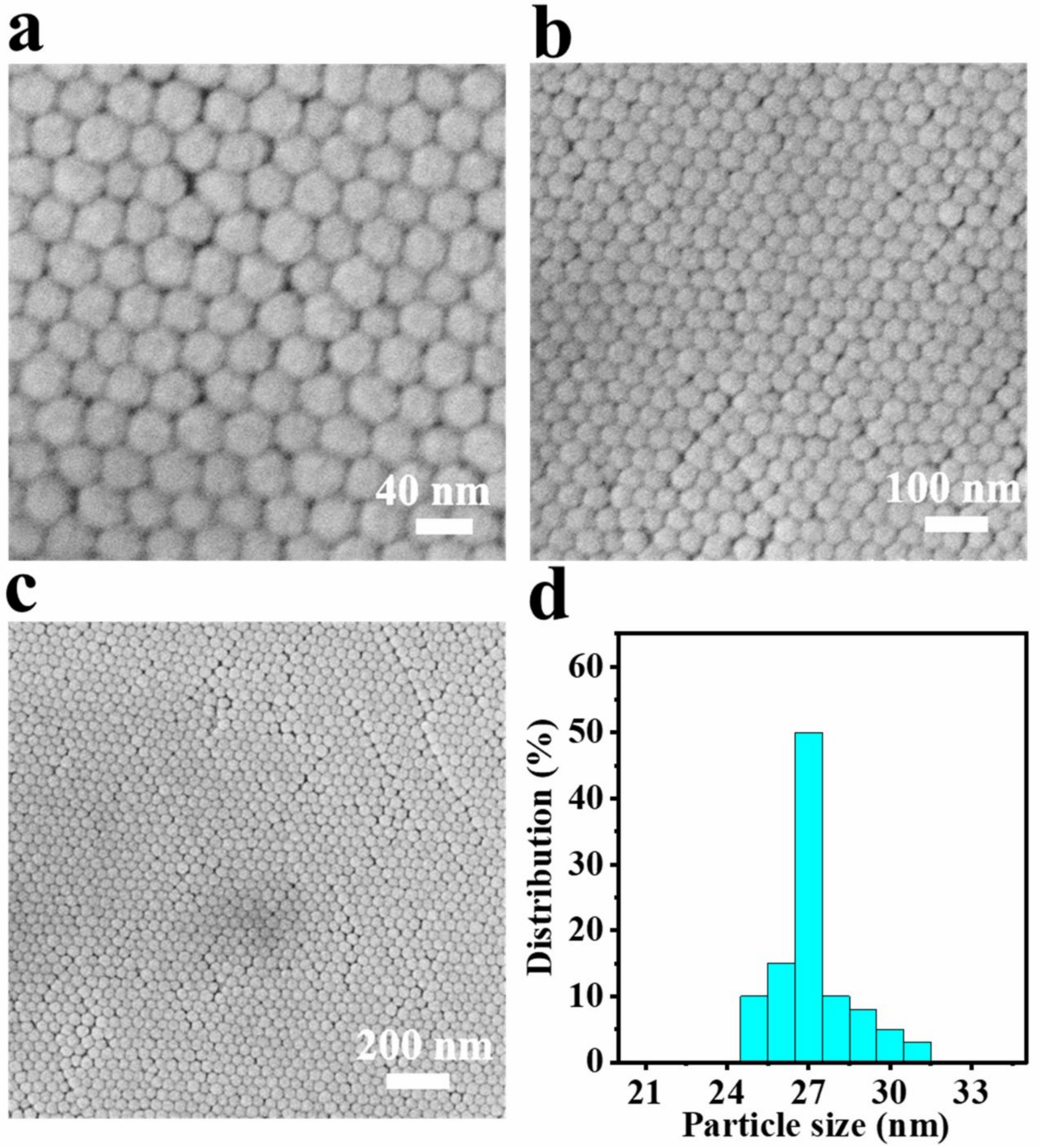

Figure S1. (a-c) SEM images and (d) the corresponding particle size distribution of 3DO-SiO_2 with an average particle size of ~27 nm, which were employed as a hard template for fabricating 3DOm-SnO_2.

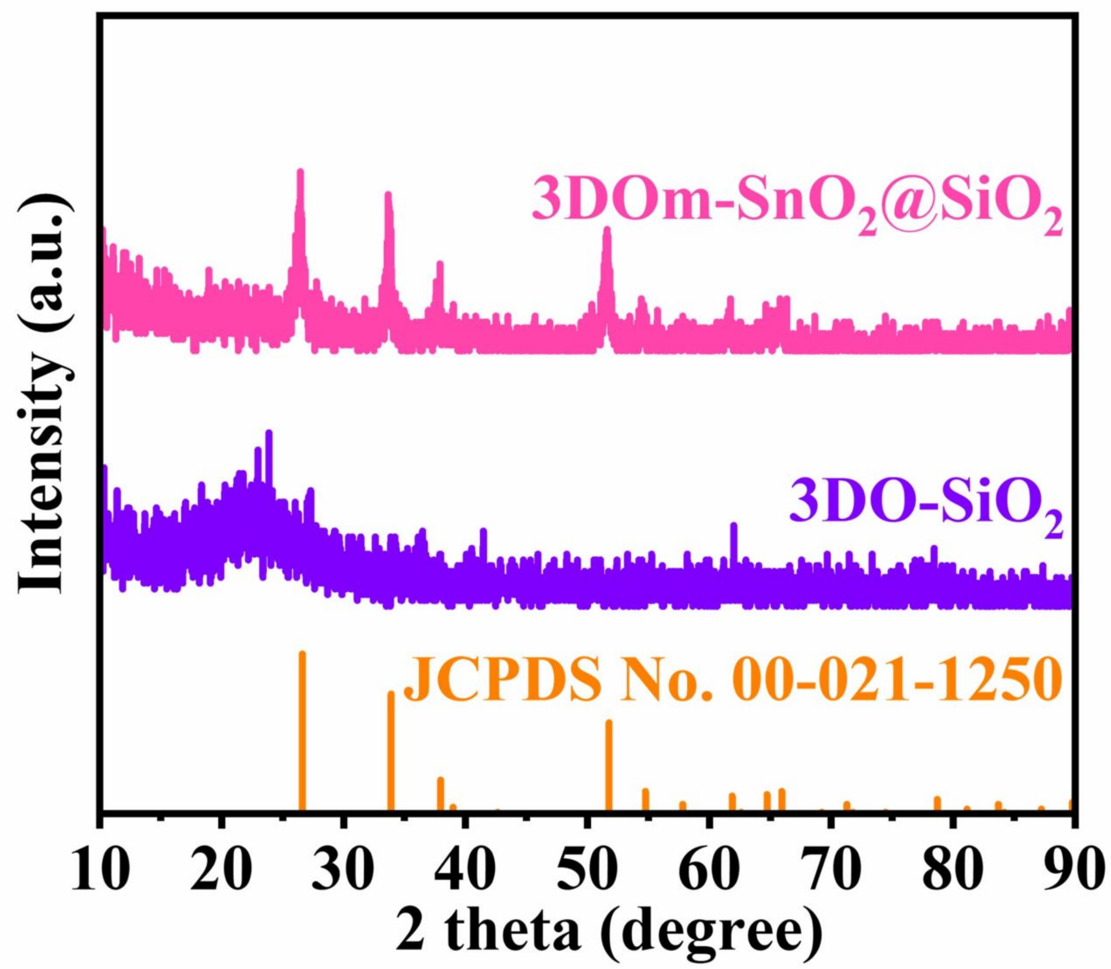

Figure S2. XRD patterns of 3DO-SiO_2 and 3DOm-SnO_2@SiO_2 and the standard rutile SnO_2 (JCPDS No. 00-021-1250), which confirm the successful formation of crystalline SnO_2 species in 3DO-SiO_2 after the hydrothermal process.

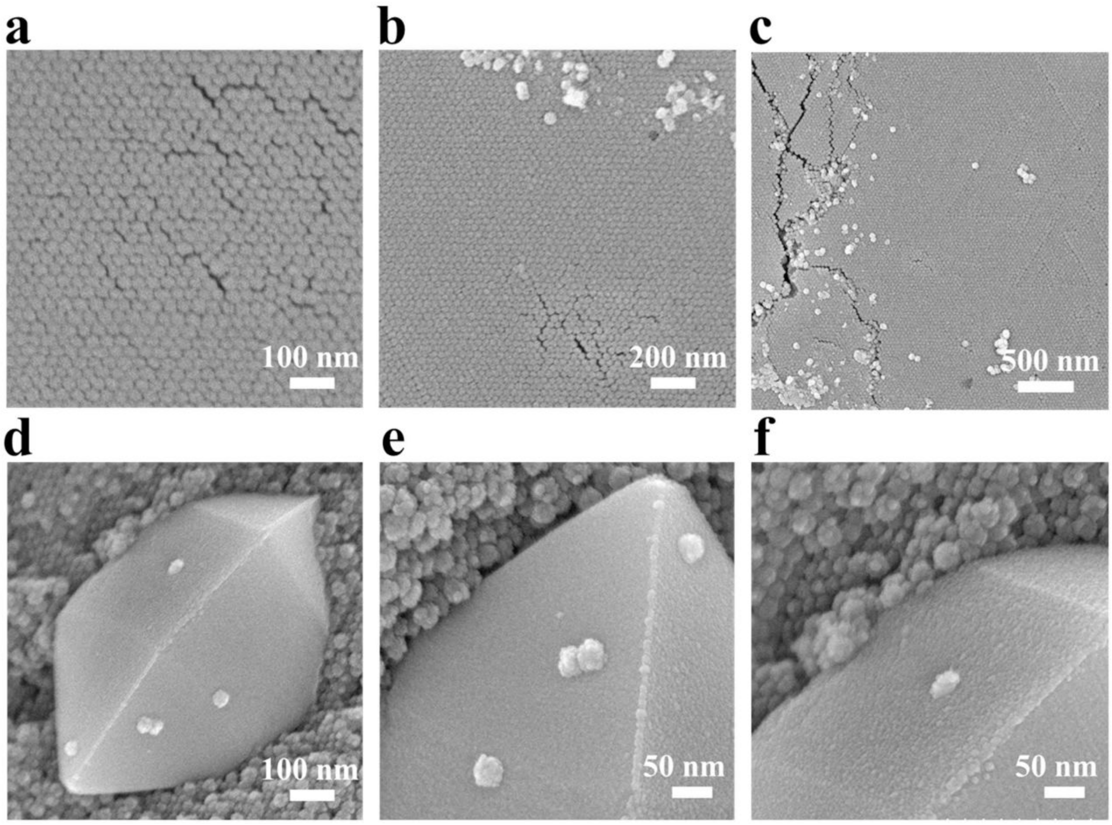

Figure S3. (a-c) SEM images of the surface of 3DOm-SnO_2@SiO_2 after the hydrothermal process to in-situ grow 3DOm-SnO_2 in the mesoscopic periodic voids of the 3DO-SiO_2 mould. (d-f) SEM images of an individual partly-templated 3DOm-SnO_2 single crystals growing on the surface of 3DOm-SnO_2@SiO_2.

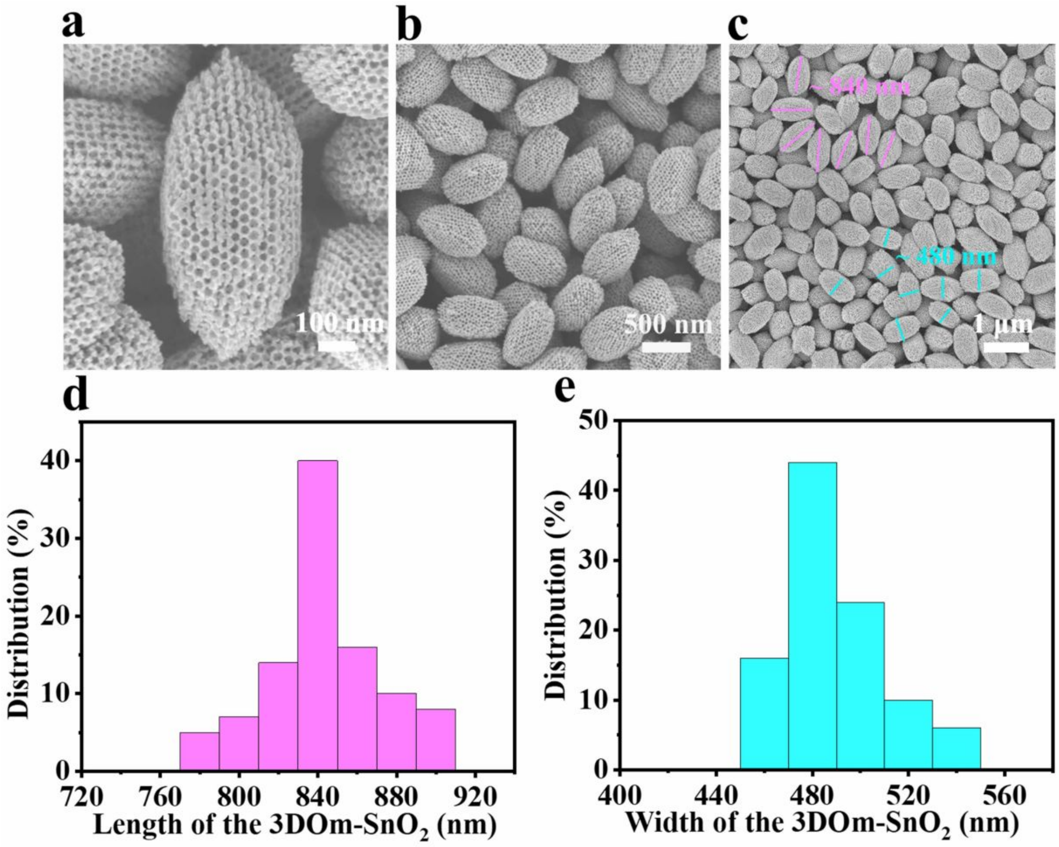

Figure S4. (a-c) SEM images and (d, e) length and width distributions of 3DOm-SnO_2. Clearly, 3DOm-SnO_2 has a well-developed olivary morphology with a uniform size of ~480 nm in width and ~840 nm in length.

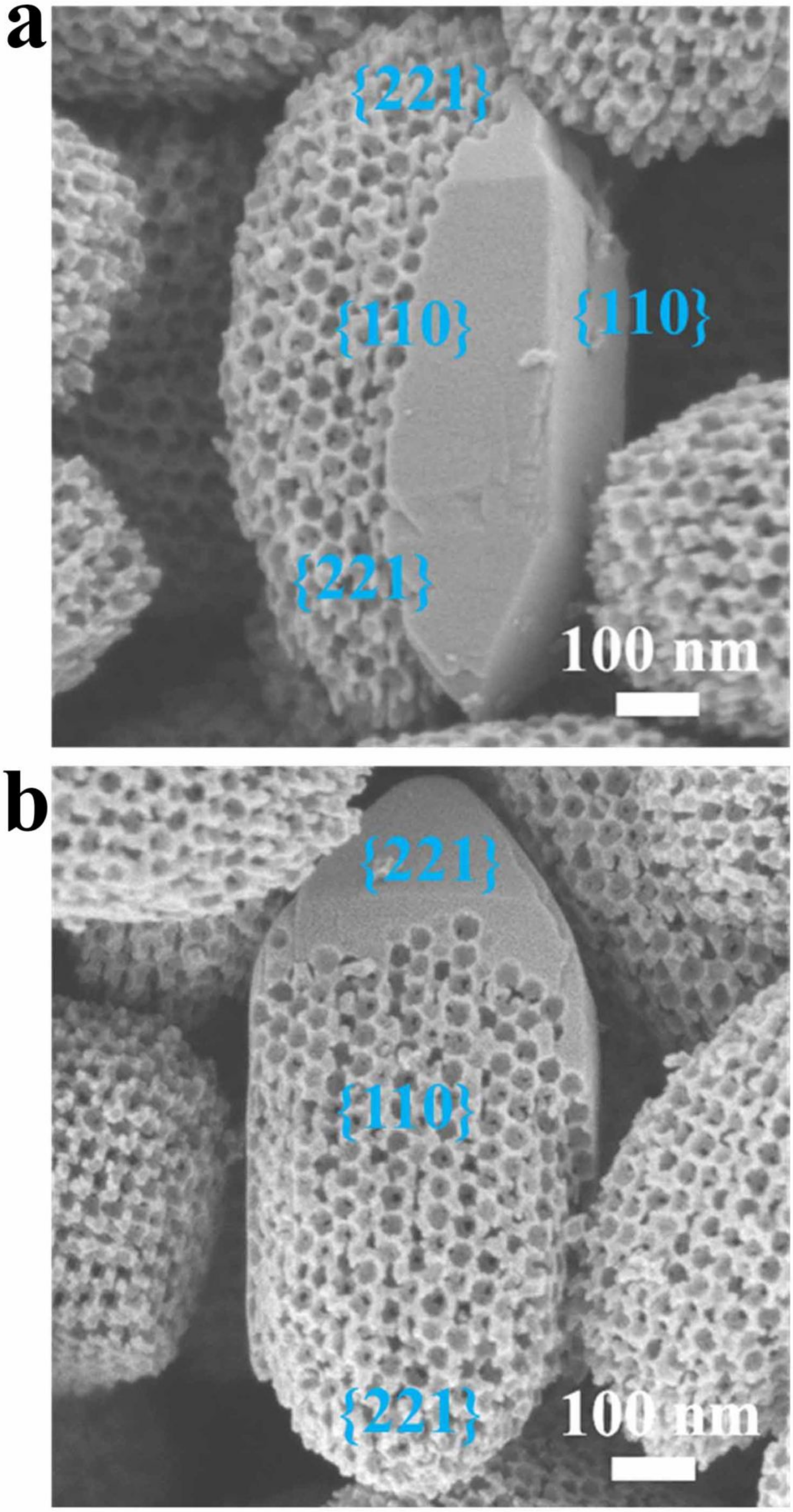

Figure S5. (a, b) SEM images of some partly-templated 3DOm-SnO_2 single crystals with well-defined facets. These untemplated parts show an unambiguous tetrakaidecahedron morphology with four {110} and eight {221} facets, suggesting the single-crystalline nature of 3DOm-SnO_2.

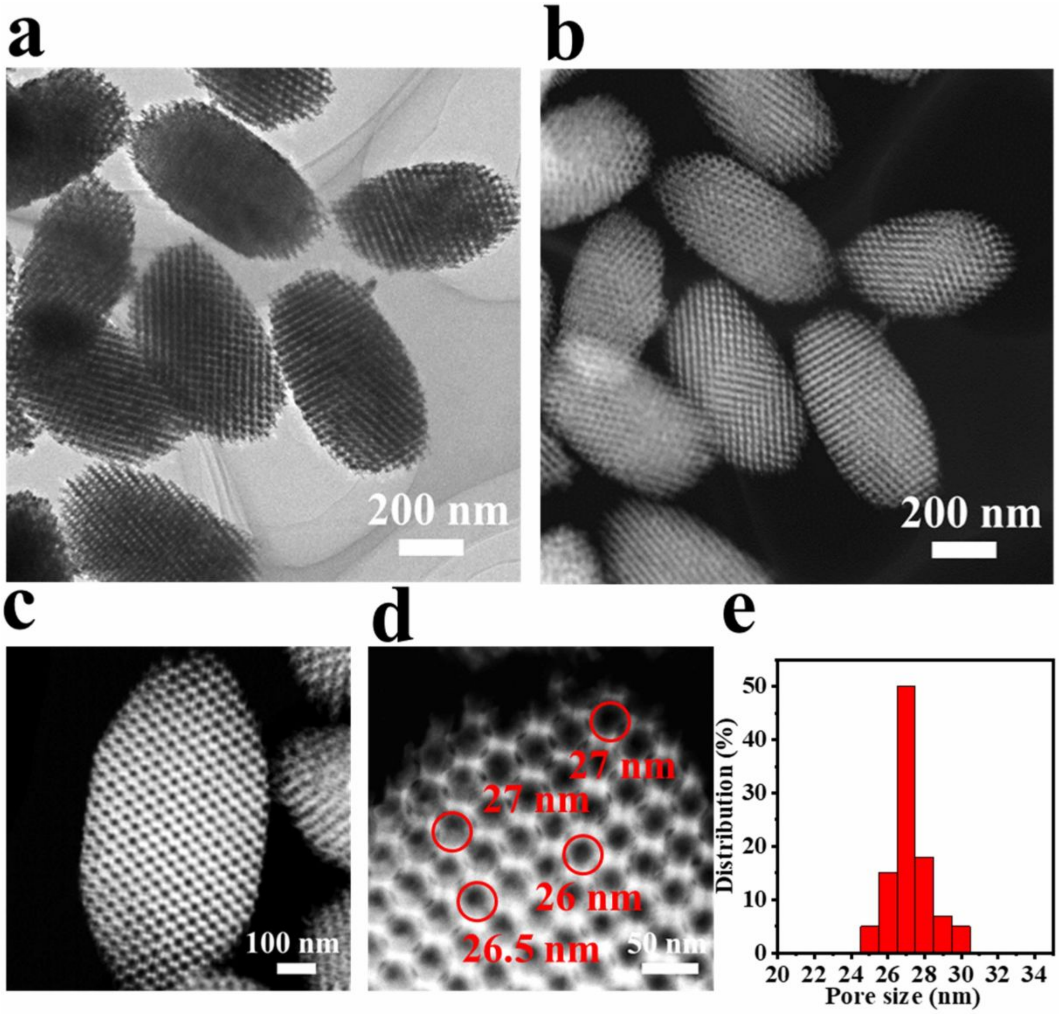

Figure S6. (a) TEM images, (b-d) STEM images and (e) pore size distribution of 3DOm-SnO_2. The highly-ordered mesopores with a uniform diameter of ~27 nm can be clearly observed from the surface to the internal center in 3DOm-SnO_2, which endows 3DOm-SnO_2 with larger active surface, faster mass transfer and higher storage volume as relative to its solid SnO_2 counterpart.

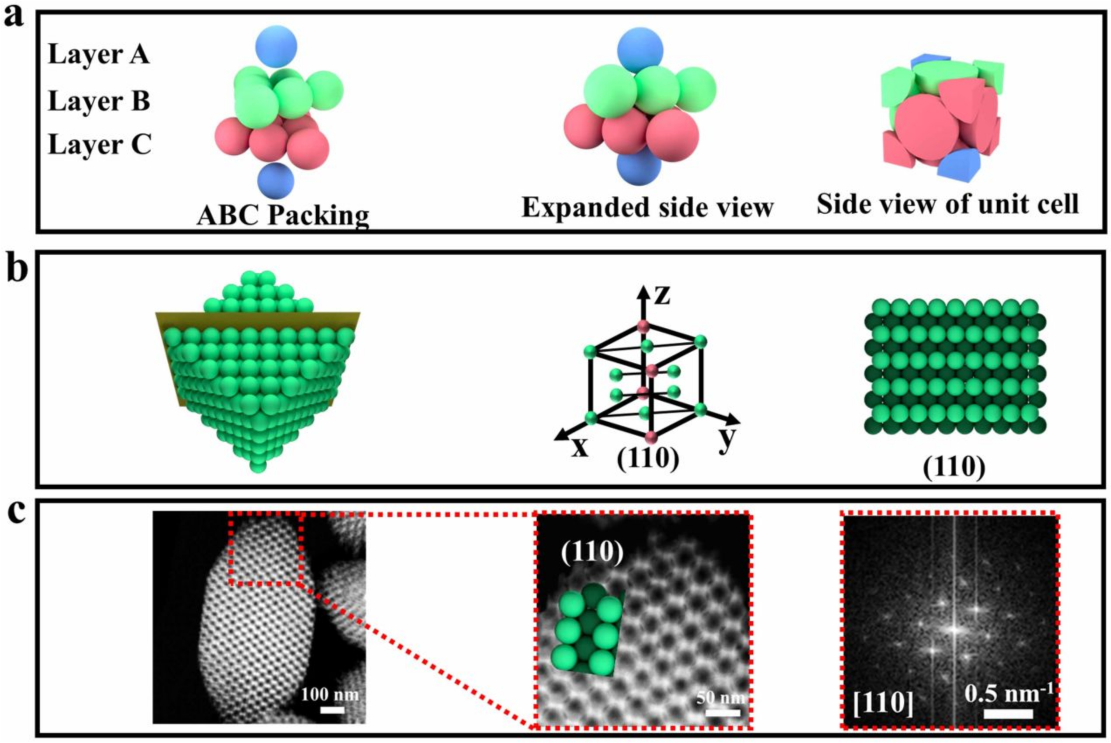

Figure S7. (a) ABCABC… packing models of the face centered cubic (FCC) arrangement. (b) Pore arrangement on the (110) plane of the FCC arrangement. (c) Cs-corrected STEM images and the corresponding FT pattern of 3DOm-SnO_2, whose ordered mesopores correspond to the (110) planes in an FCC arrangement with a high interconnectivity.

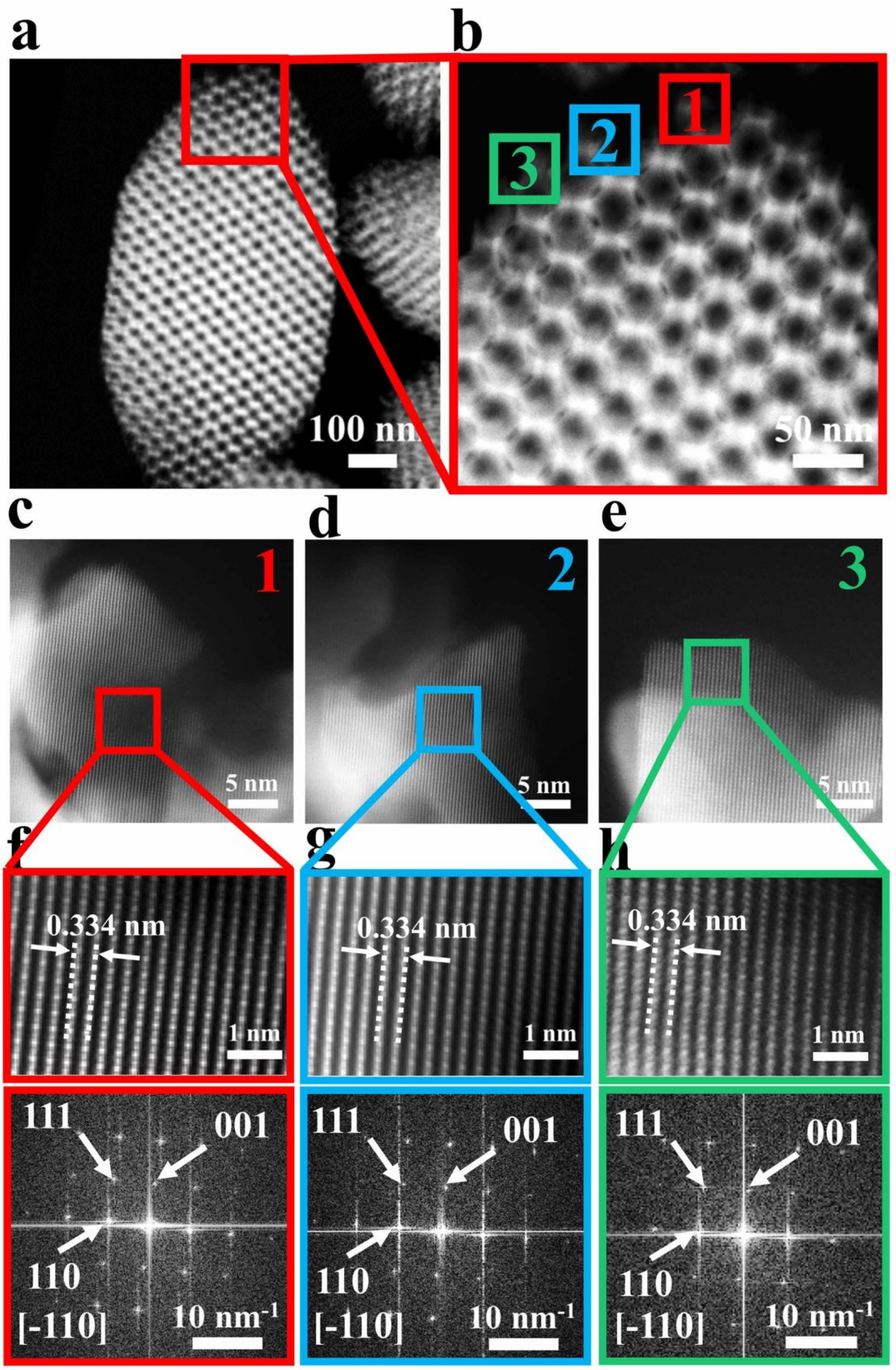

Figure S8. (a-h) Cs-corrected STEM images at different magnifications and their corresponding FFT patterns of 3DOm-SnO_2. The Cs-corrected STEM images of three different positions taken from the same 3DOm-SnO_2 particle show the same clear lattice fringe spacing of 0.334 nm, which is consistent with the (110) planes of tetragonal SnO_2, in line with the corresponding Fast Fourier Transform (FFT) patterns.

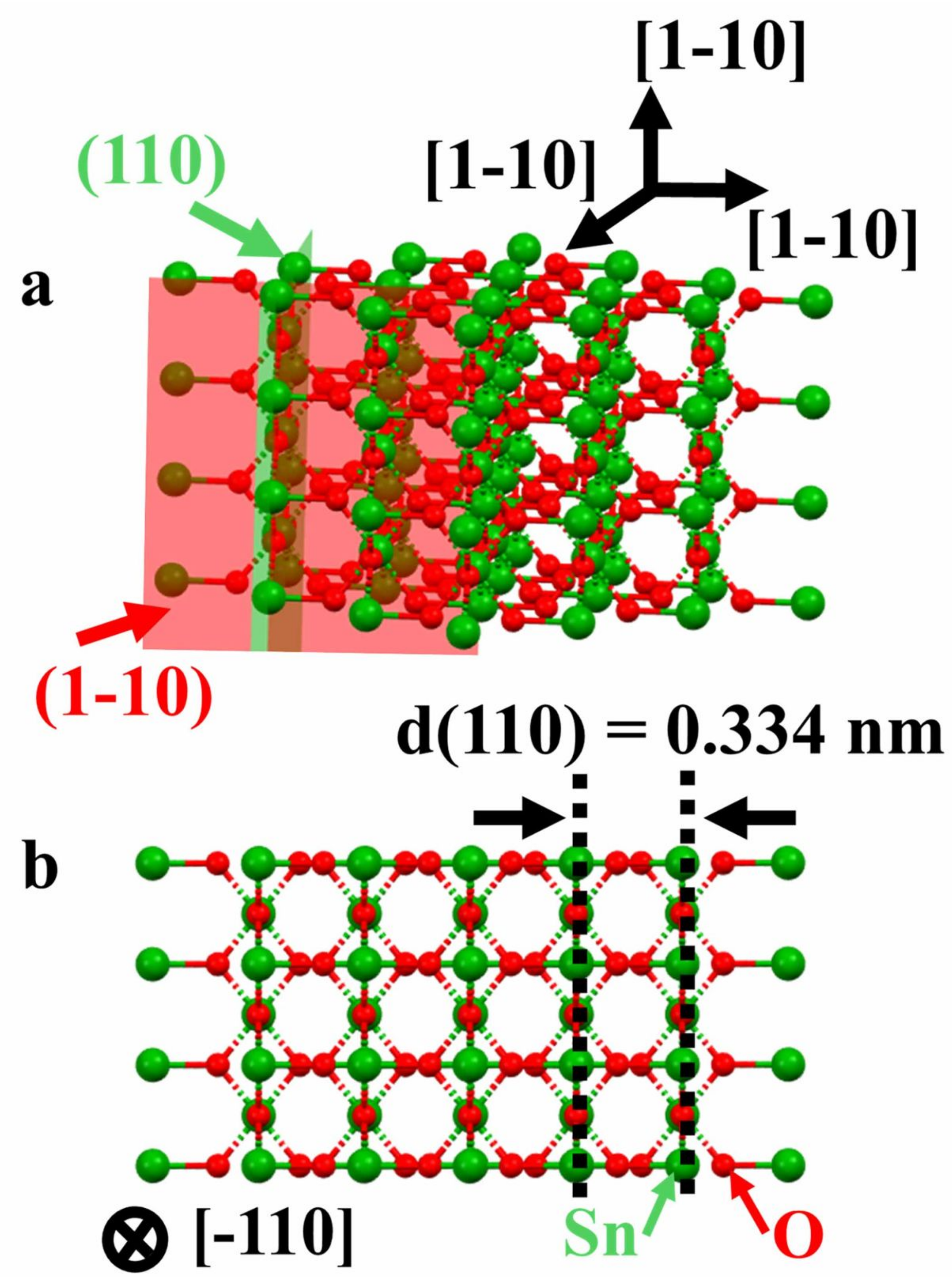

Figure S9. (a) three-dimensional and (b) two-dimensional atomic models of 3DOm-SnO_2, which show the atomic structure of the (110) plane of tetragonal SnO_2 with a lattice fringe spacing of ~ 0.334 nm, which is in good agreement with the above atomic-resolution STEM images in Figure S8.

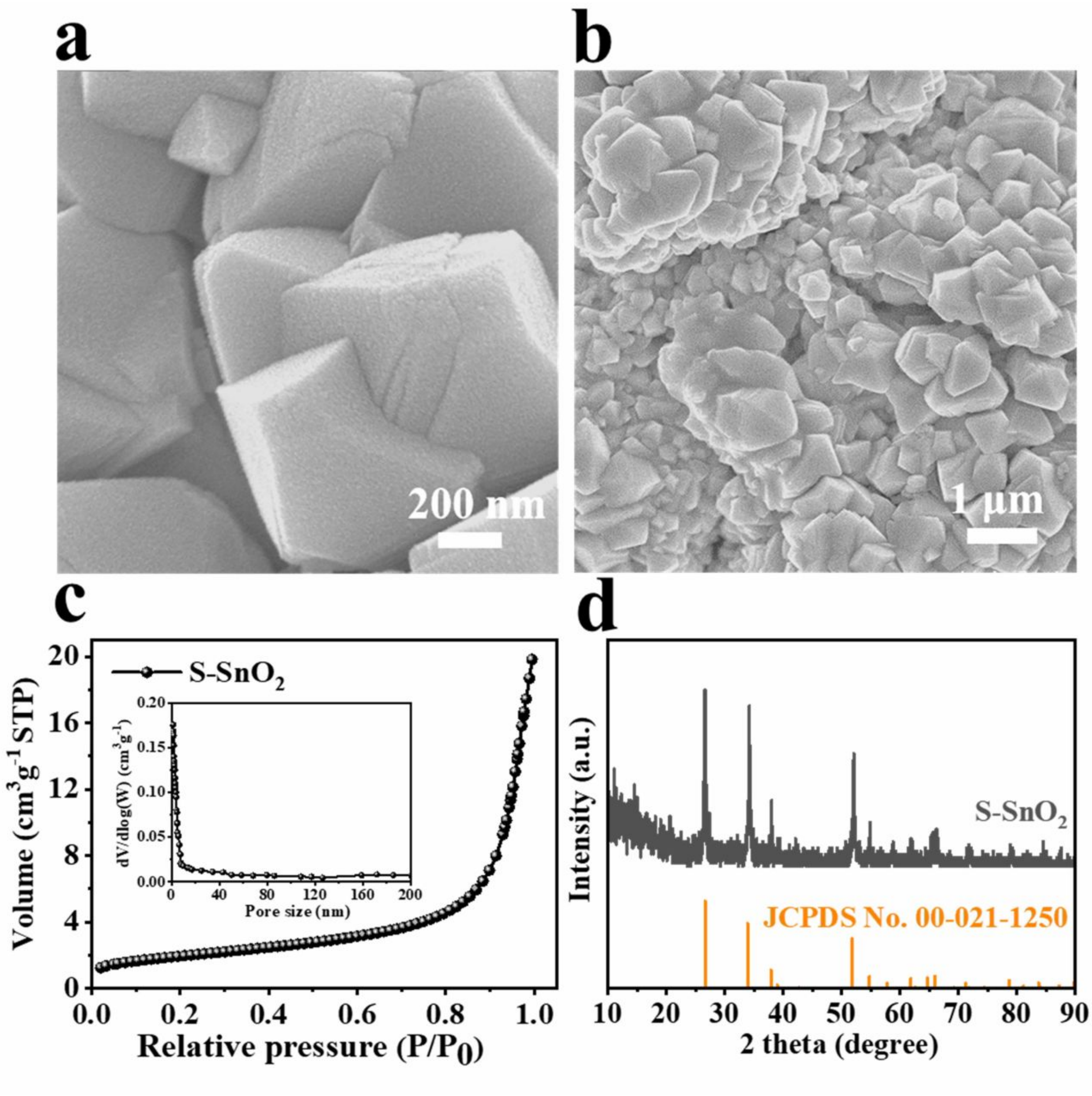

Figure S10. (a, b) SEM images, (c) N_2 adsorption/desorption isotherm and (d) XRD pattern of S-SnO_2. The inset of e is the corresponding pore size distribution (based on DFT model by using the desorption branch of the isotherms). Obviously, the S-SnO_2 particles prepared in the absence of 3DO-SiO_2 aggregate severely with no identifiable facets.

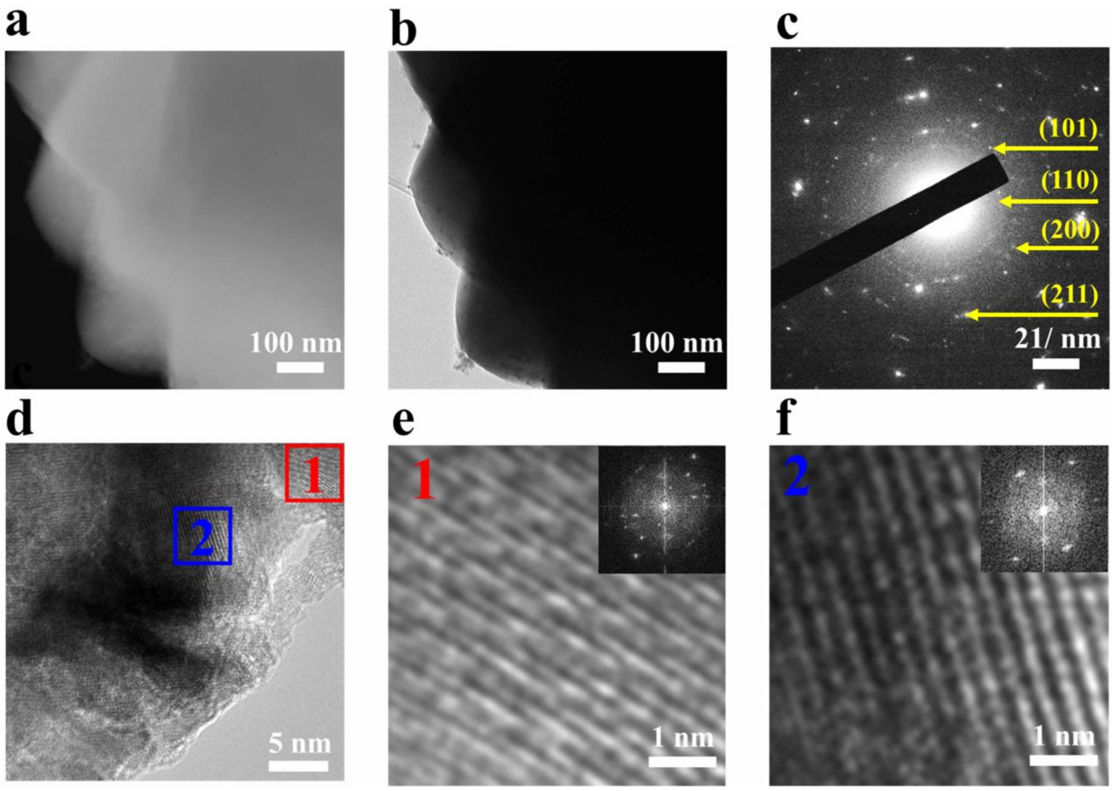

Figure S11. (a) STEM image, (b, d) TEM images, (c) SAED pattern of S-SnO_2. (e, f) High-resolution TEM images and the corresponding FFT patterns of S-SnO_2. The SAED pattern taken from an isolated S-SnO_2 particle reveals its polycrystalline characteristic of diffraction rings, which is also confirmed by the corresponding high-lattice resolution TEM images, where different regions show different lattice streaks.

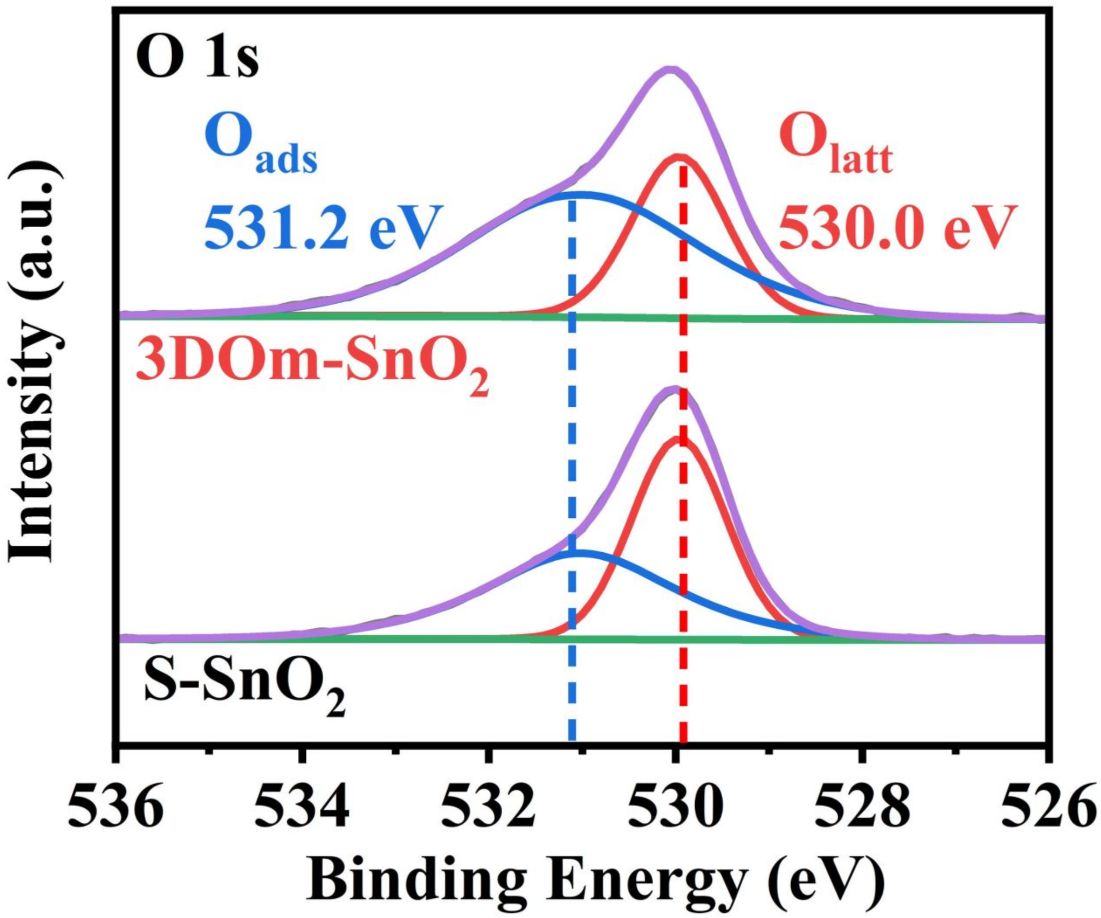

Figure S12. High-resolution O 1s XPS spectra of 3DOm-SnO_2 and S-SnO_2 show two characteristic peaks at 530.0 eV and 531.2 eV, which can be assigned to the lattice oxygen species in SnO_2 lattices (denoted as O_{latt}) and the surface adsorbed oxygen species with unsaturated coordination (denoted as O_{ads}), respectively. It is generally recognized that the unsaturated O_{ads} can lead to the formation of favorable oxygen vacancies on catalyst surface. As indicated by Table S3, 3DOm-SnO_2 has a much higher O_{ads} / (O_{ads} + O_{latt}) ratio (64.57%) than S-SnO_2 (47.51%), revealing the formation of more oxygen vacancies in its 3DOm structure, which can not only facilitate the dispersion and stabilization of active metal species by forming strong metal–support interaction, but also provide abundant adsorption sites for reactants/intermediates.

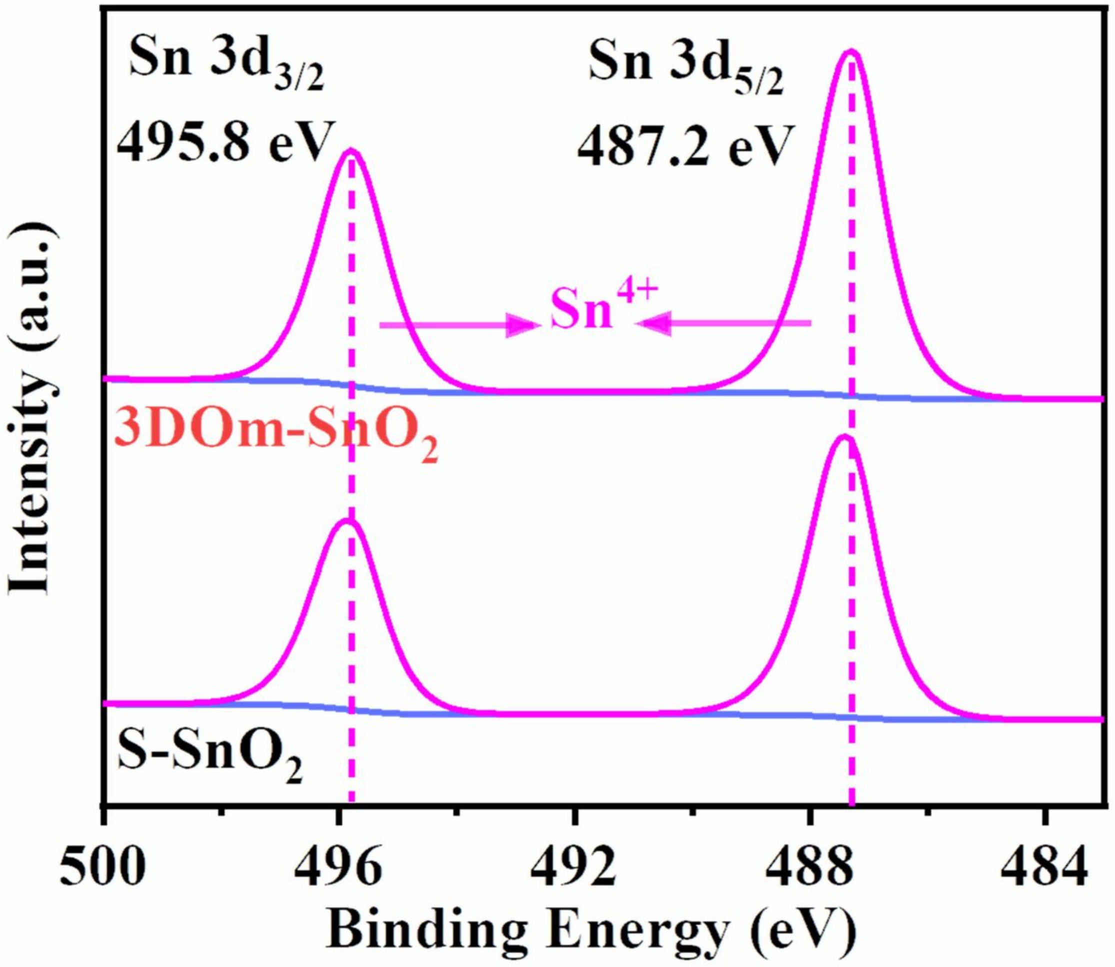

Figure S13. High-resolution Sn 3d XPS spectra of 3DOm-SnO_2 and S-SnO_2, which show two peaks at 495.8 eV and 487.2 eV corresponding to the Sn $3d_{3/2}$ and Sn $3d_{5/2}$ of Sn^{4+} oxidation state, being the characteristic of SnO_2 species.

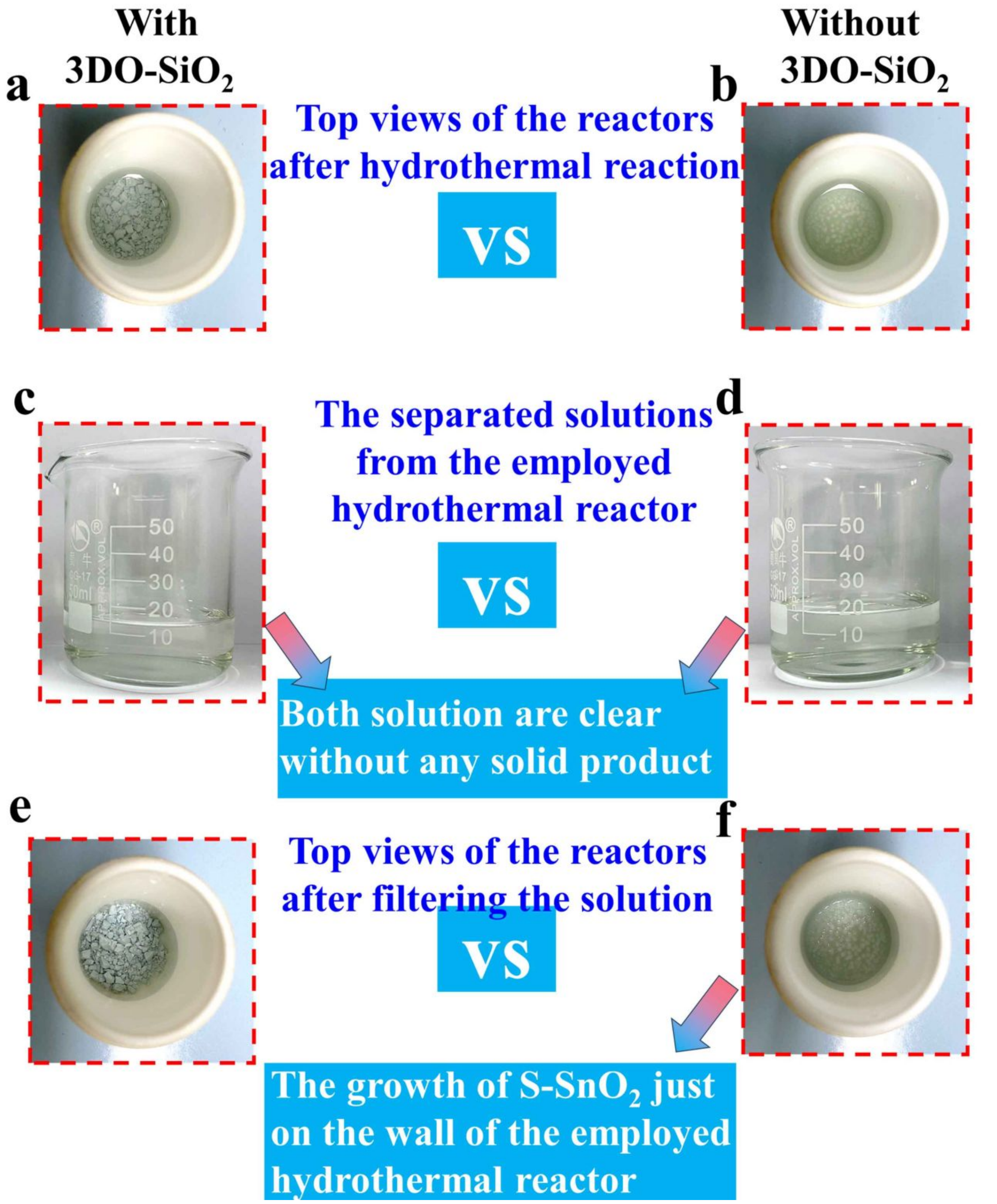

Figure S14. Photographs of the reactors after a hydrothermal process with 3DO-SiO_2 (a) vs without 3DO-SiO_2 (b). Photographs of the separated solutions from the employed hydrothermal reactor with 3DO-SiO_2 (c) vs without 3DO-SiO_2 (d). Photographs of the top views of the reactors after filtering the solution with 3DO-SiO_2 (e) vs without 3DO-SiO_2 (f). We can clearly see that the separated solution from 3DOm-SnO_2@SiO_2 is still clear without containing any solid product, which reveals that SnO_2 cannot nucleate in bulk solution, in good accordance with the growth of S-SnO_2 with a very low yield of ~1.5% just on the wall of the employed hydrothermal reactor.

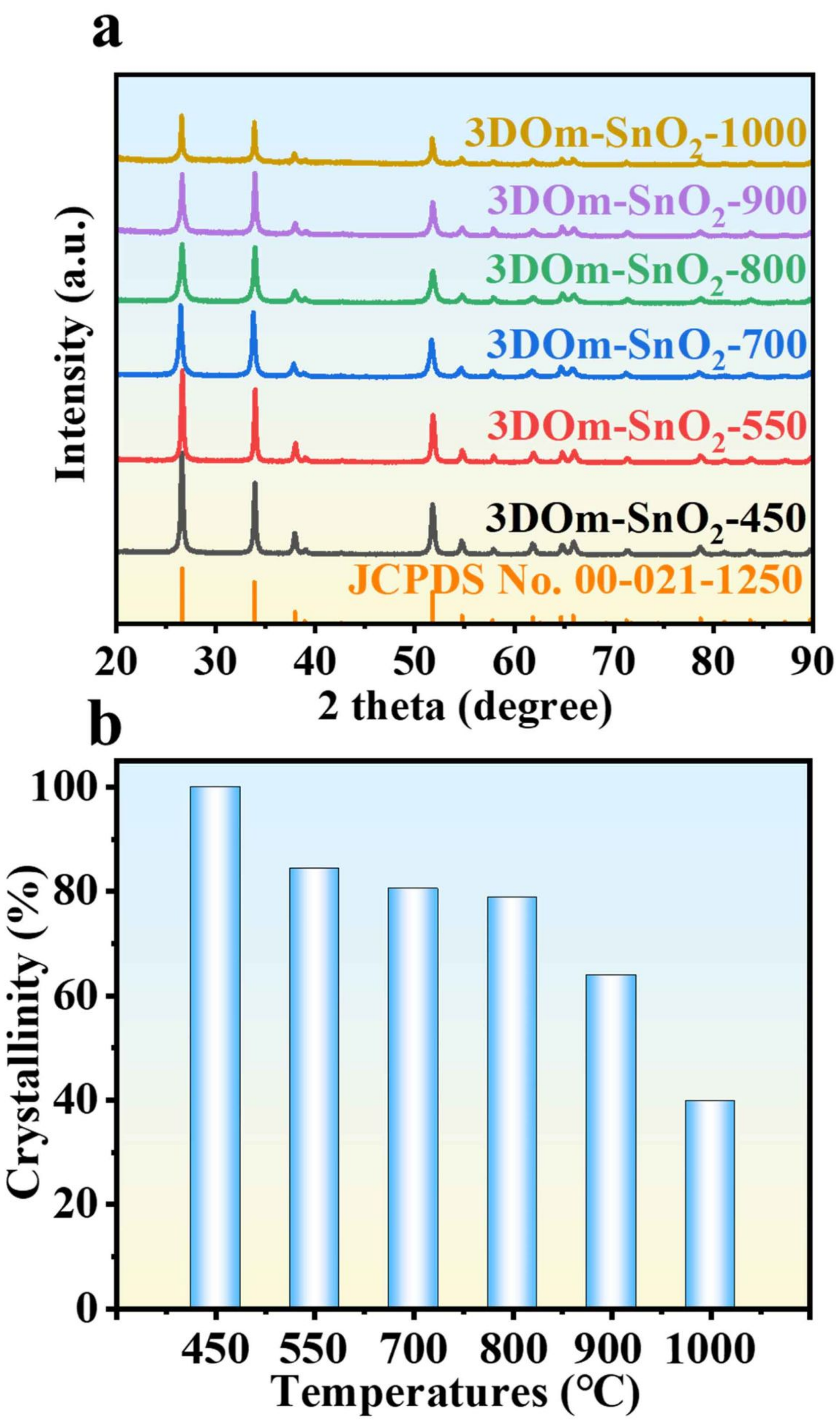

Figure S15. (a) XRD patterns of various 3DOm-SnO_2-T samples. (b) Crystallinities of various 3DOm-SnO_2-T samples as a function of the calcination temperature (assuming that the crystallinity of 3DOm-SnO_2-450 is 100%).

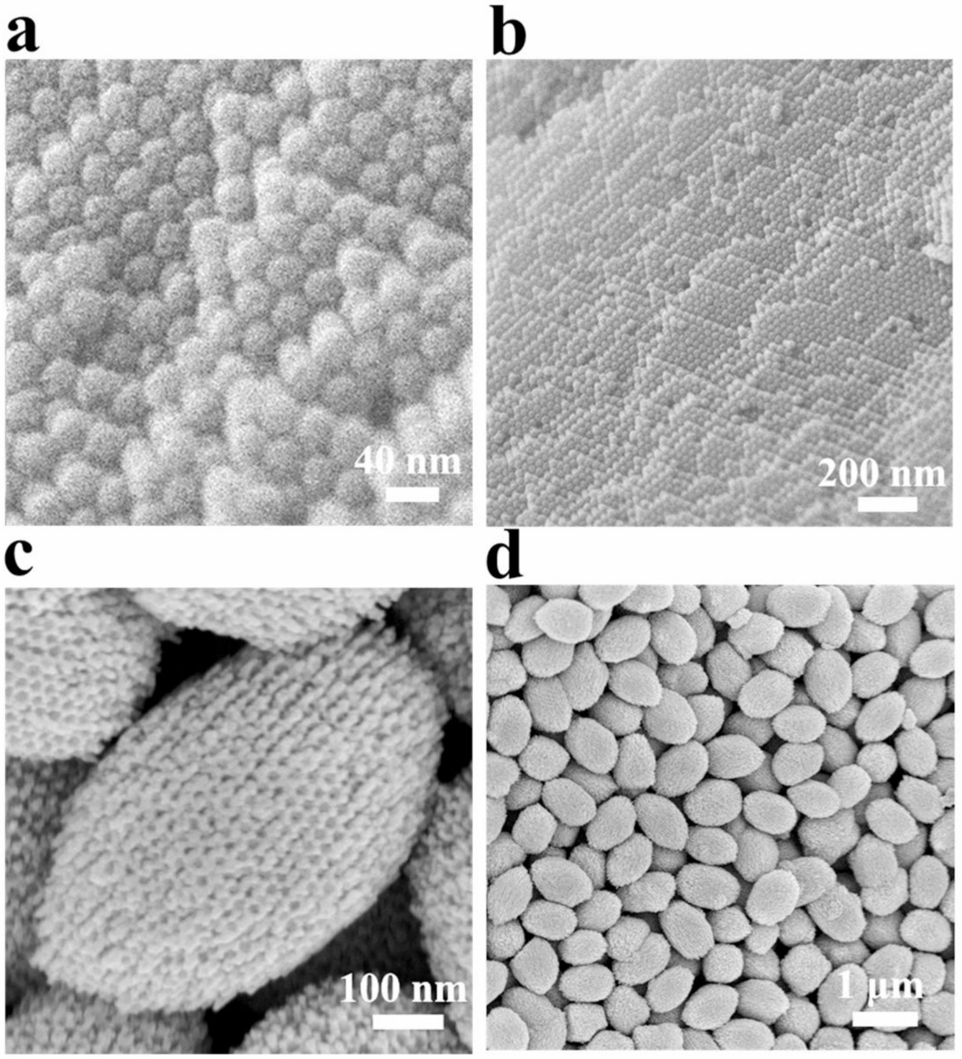

Figure S16. SEM images of (a, b) 3DO-SiO_2-550 and (c, d) 3DOm-SnO_2-550 at different magnifications.

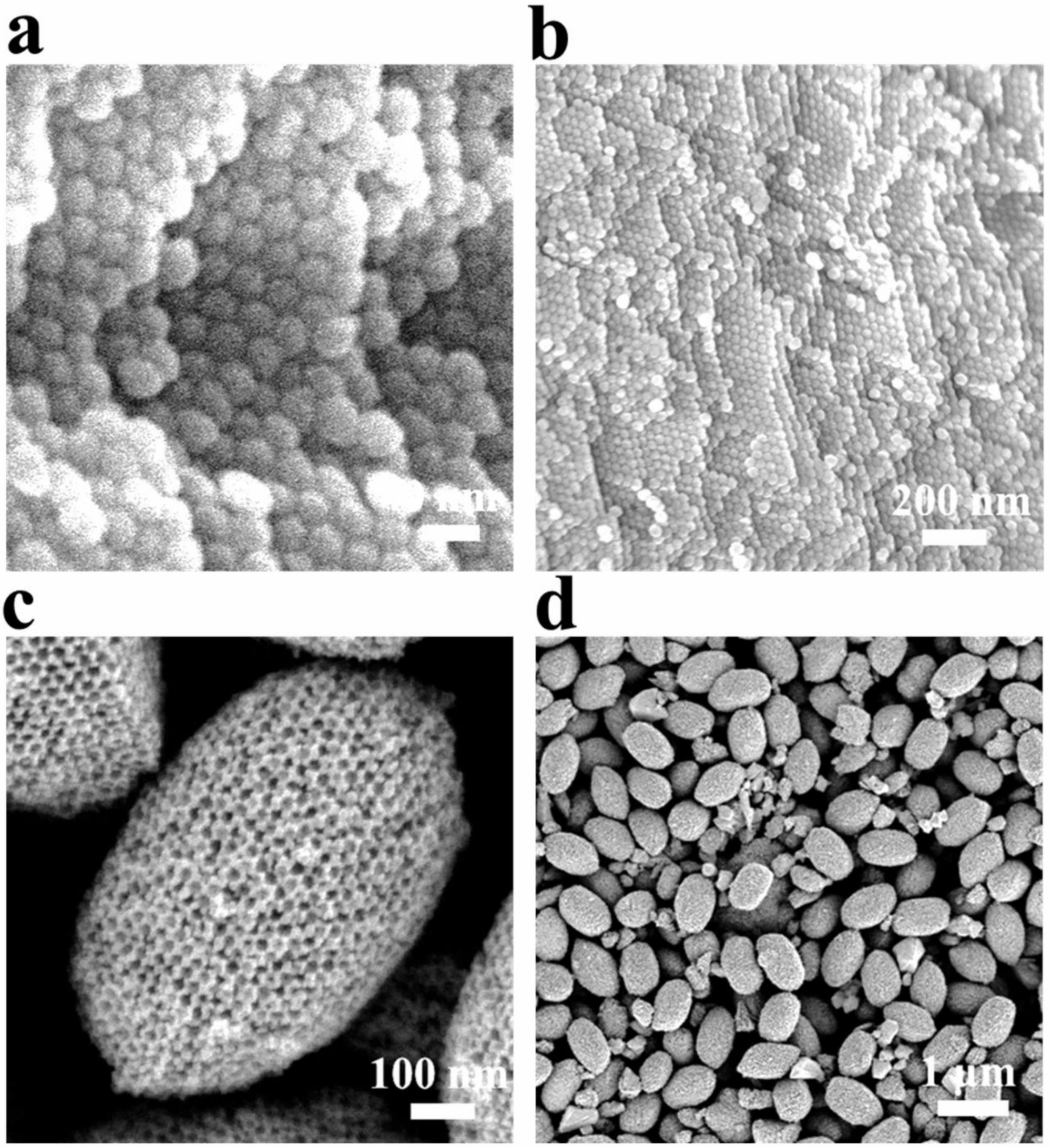

Figure S17. SEM images of (a, b) 3DO-SiO_2-700 and (c, d) 3DOm-SnO_2-700 at different magnifications.

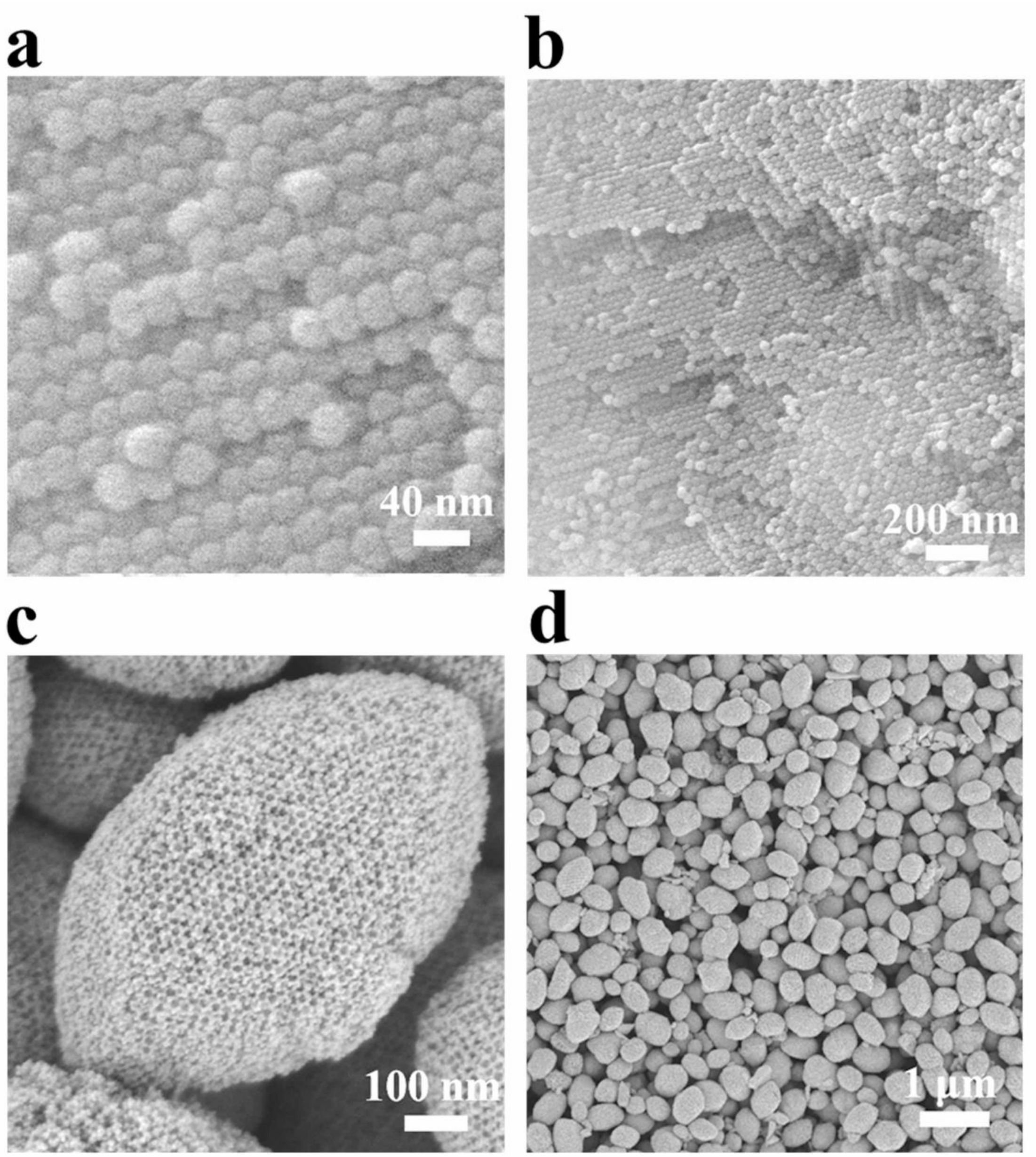

Figure S18. SEM images of (a, b) 3DO-SiO_2-800 and (c, d) 3DOm-SnO_2-800 at different magnifications.

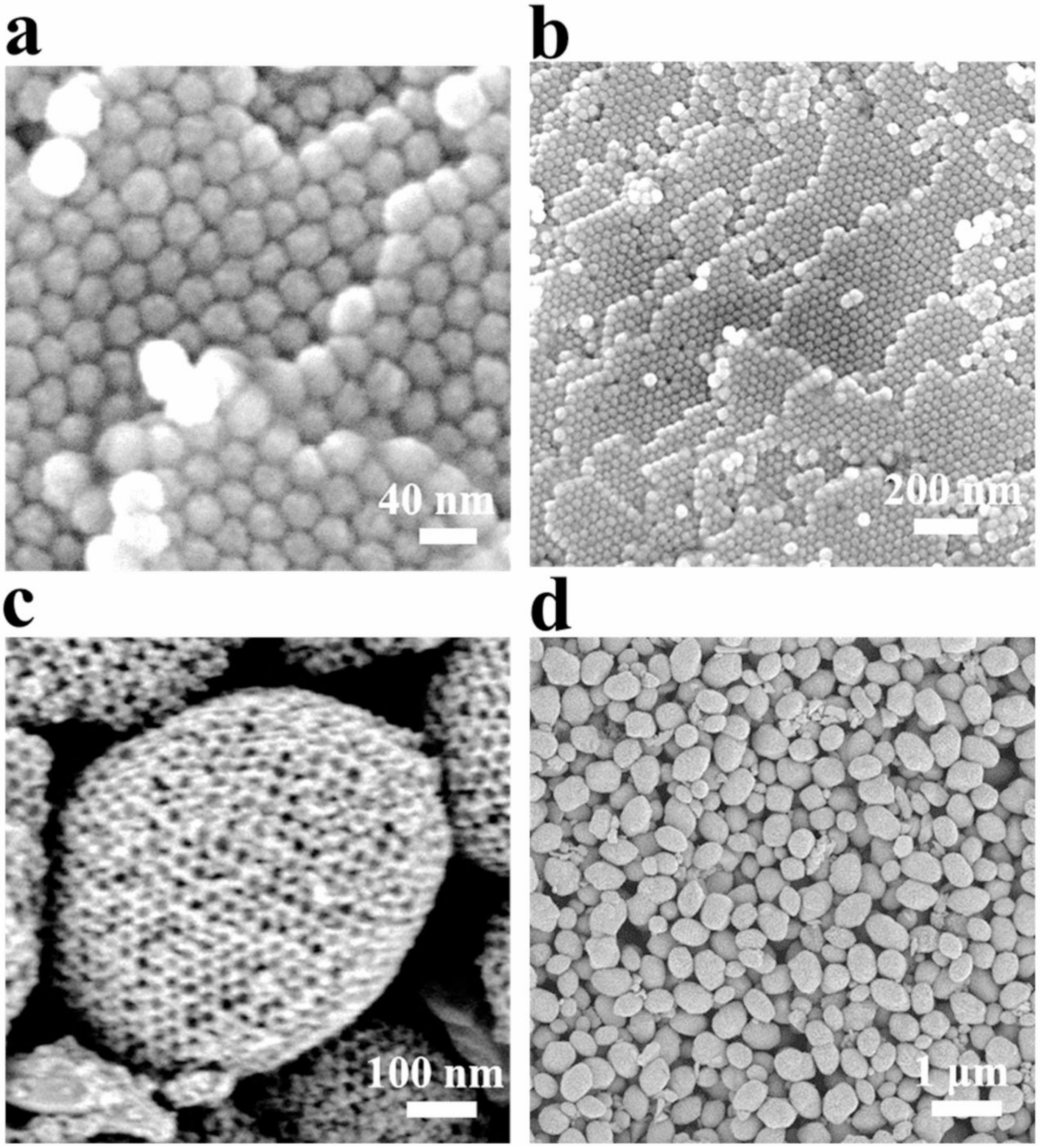

Figure S19. SEM images of (a, b) 3DO-SiO_2-900 and (c, d) 3DOm-SnO_2-900 at different magnifications.

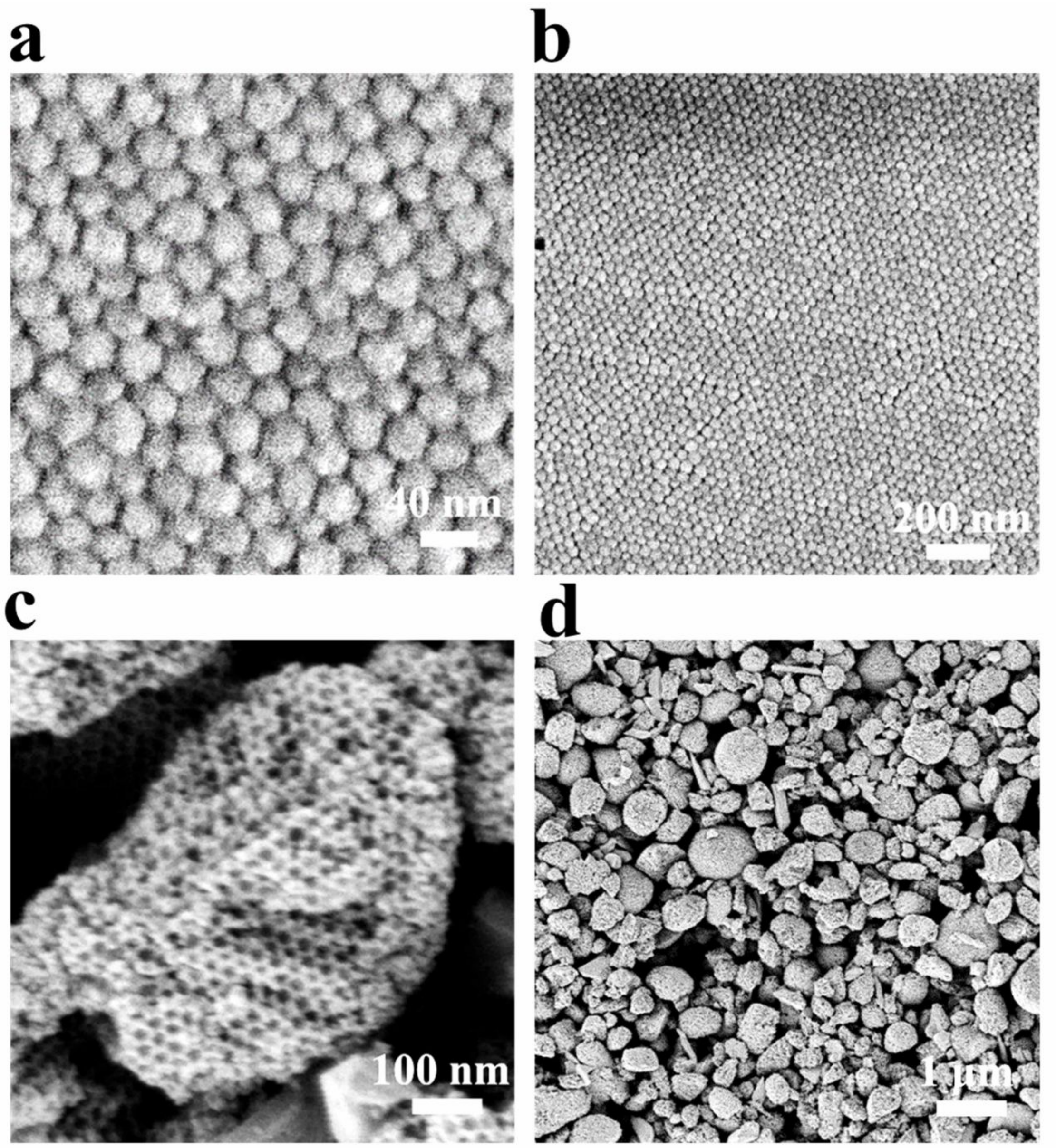

Figure S20. SEM images of (a, b) 3DO-SiO_2-1000 and (c, d) 3DOm-SnO_2-1000 at different magnifications.

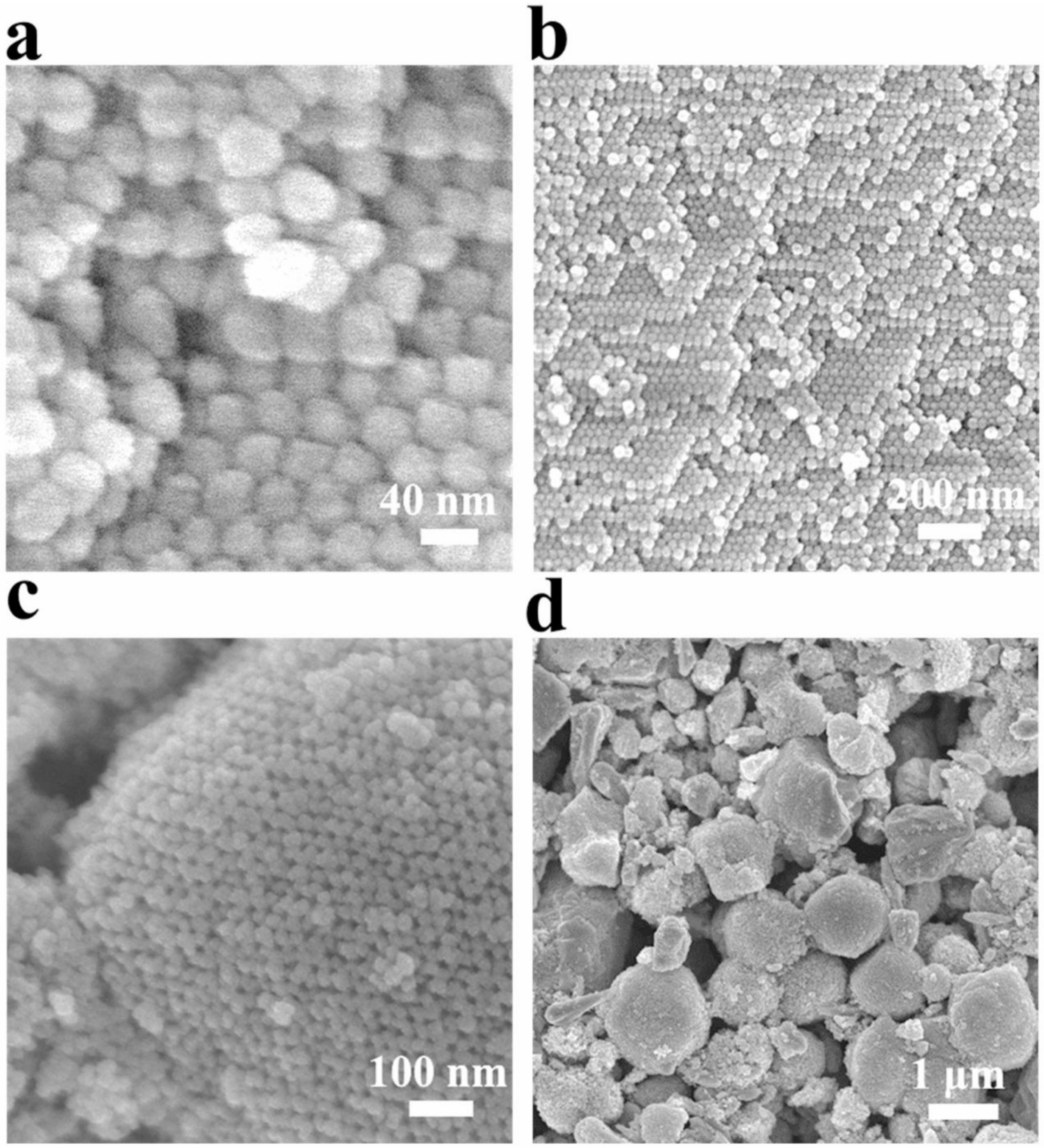

Figure S21. SEM images of (a, b) 3DO-SiO_2-M and (c, d) 3DOm-SnO_2-M at different magnifications.

$$Si\text{-}OH + H_2O\ (g) \underset{\text{High P}}{\overset{\text{High T}}{\rightleftharpoons}} Si\text{-}O^- + H_3O^+$$

Figure S22. The possible dynamic equilibrium reaction of Si-OH on the surface of 3DO-SiO_2 under hydrothermal conditions.

According to Le chtelier's principle, since the above reaction is endothermic and decompressive, increasing the temperature and pressure can pushe this reaction in a positive direction, resulting in more Si-O^- being generated. The resultant abundant Si-O^- groups can capture Sn^{4+} ions by forming Si-O-Sn groups due to the coulomb interaction between Si-O^- groups and Sn^{4+} ions based on the classical hard-soft-acid-base theory. Subsequently, with the enrichment of Sn in 3DO-SiO_2, SnO_2 can spontaneously nucleate on the interior surface of 3DO-SiO_2, thus initiating the heterogeneous crystallization of 3DOm-SnO_2 in its mesoscopic periodic voids.

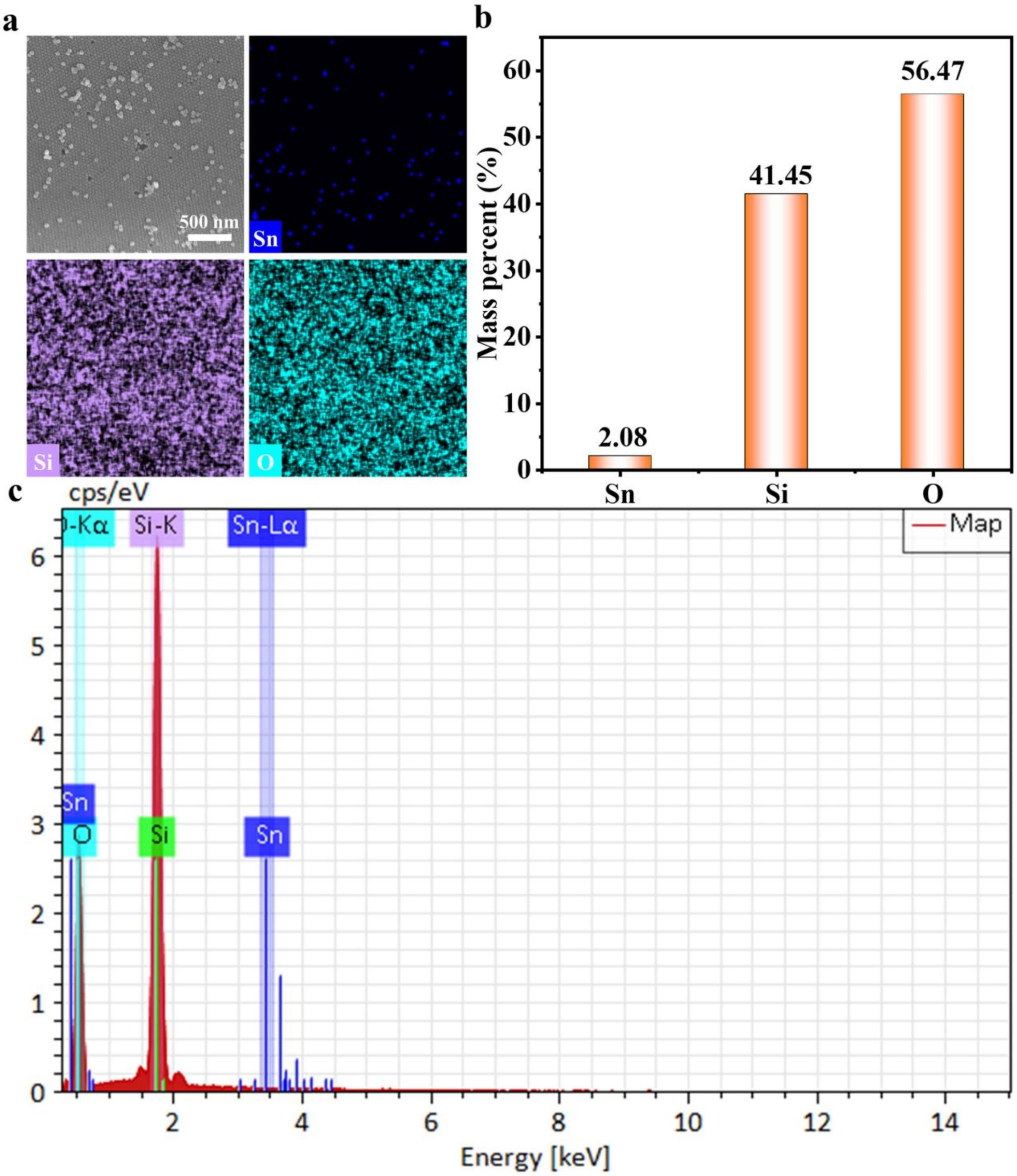

Figure S23. (a) SEM plus EDS mapping images of the 3DO-SiO_2-1000 hydrothermally treated at 150 °C for 12 h. (b) Mass percents of various elements detected on the 3DO-SiO_2-1000 hydrothermally treated at 150 °C for 12 h. (c) The corresponding elemental spectrum of the 3DO-SiO_2-1000 hydrothermally treated at 150 °C for 12 h.

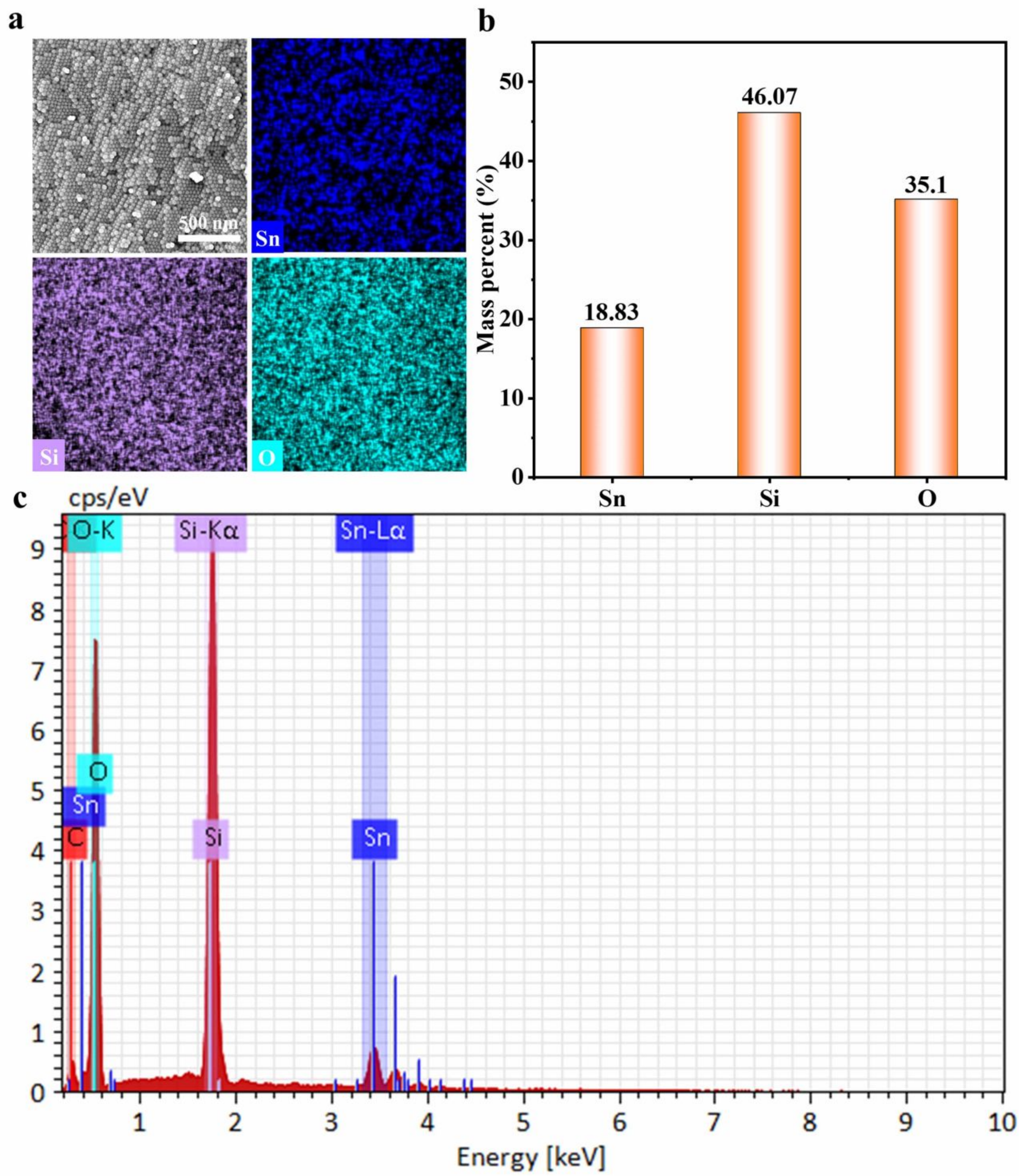

Figure S24. (a) SEM plus EDS mapping images of the 3DO-SiO_2-450 hydrothermally treated at 150 °C for 12 h. (b) Mass percents of various elements detected on the 3DO-SiO_2-450 hydrothermally treated at 150 °C for 12 h. (c) The corresponding elemental spectrum of the 3DO-SiO_2-450 hydrothermally treated at 150 °C for 12 h.

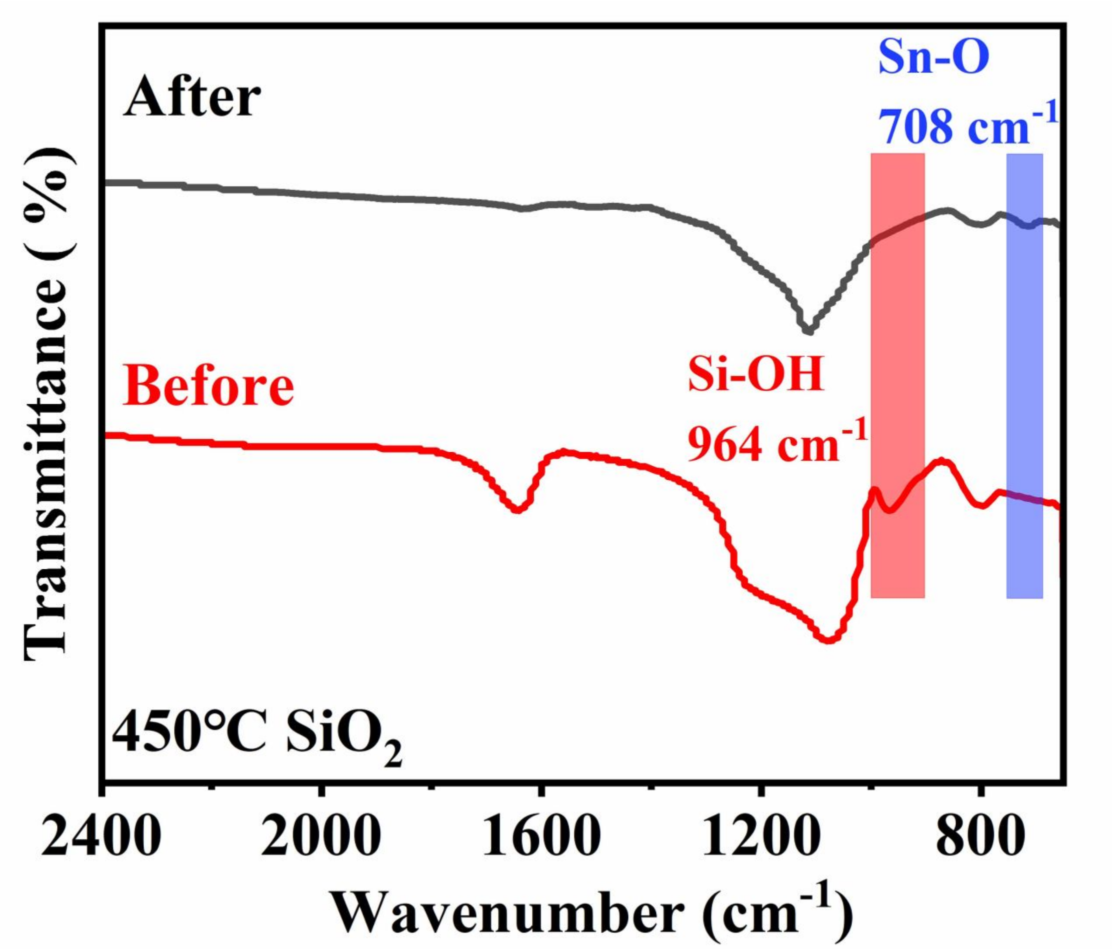

Figure S25. FTIR spectra of 3DO-SiO_2-450 and the hydrothermally-treated 3DO-SiO_2-450 at 150 °C for 24 h. The FTIR spectrum of the hydrothermally-treated 3DO-SiO_2-450 confirms that the peak of Si-OH (964 cm^{-1}) disappears with a new peak appearing at 708 cm^{-1}, which can be attributed to the stretching vibration of Sn-O bonds, revealing the strong interaction between Si-O^- and Sn^{4+}.

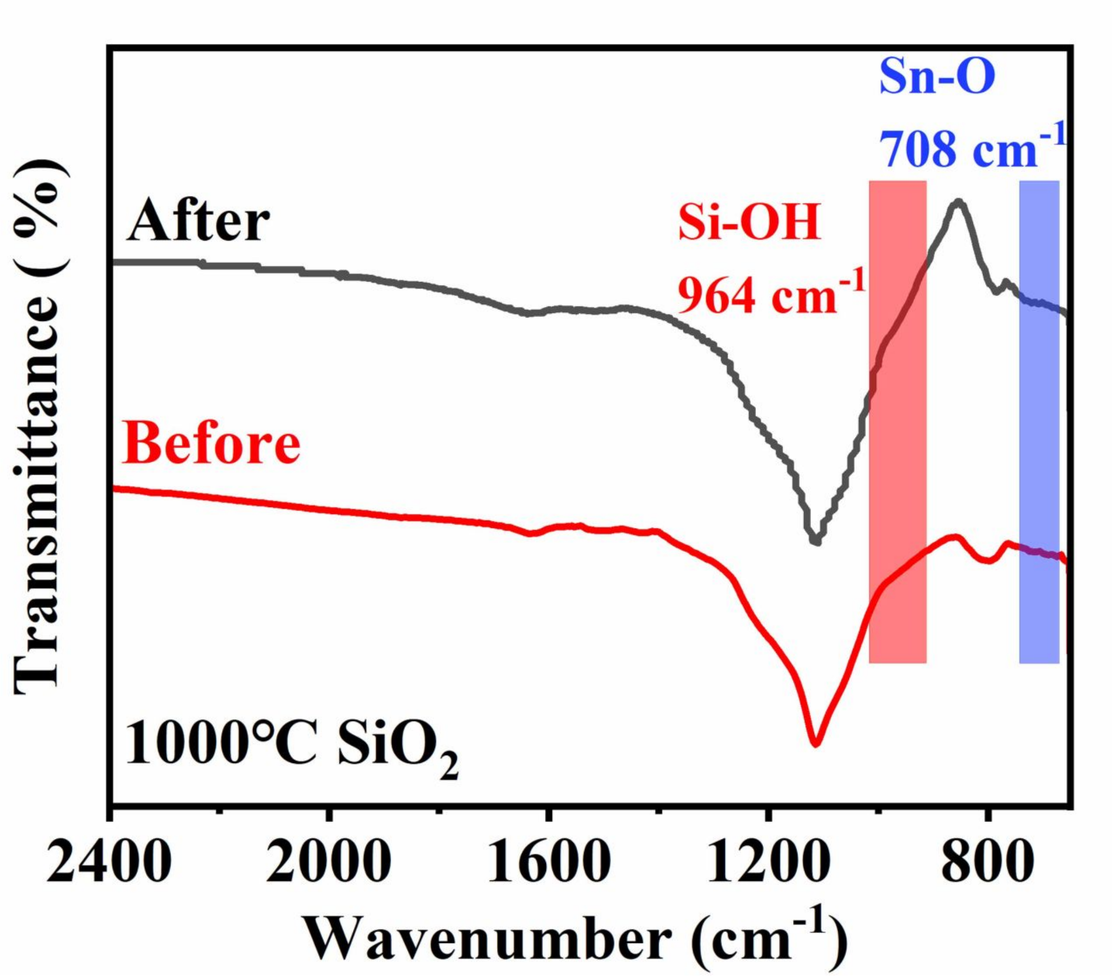

Figure S26. FTIR spectra of 3DO-SiO_2-1000 (blow) and the hydrothermally-treated 3DO-SiO_2-1000 at 150 ^{o}C for 24 h (above). The FTIR spectrum of hydrothermally-treated 3DO-SiO_2-1000 is consistent with that of the 3DO-SiO_2-1000 due to the fact that no Si-OH groups can be converted into the corresponding Sn-O groups in 3DO-SiO_2-1000. This results in the inability to enrich Sn^{4+} on the surface of 3DO-SiO_2-1000 for the spontaneous nucleation and in-situ growth of 3DOm-SnO_2 in the periodic voids of the 3DO-SiO_2-1000 template.

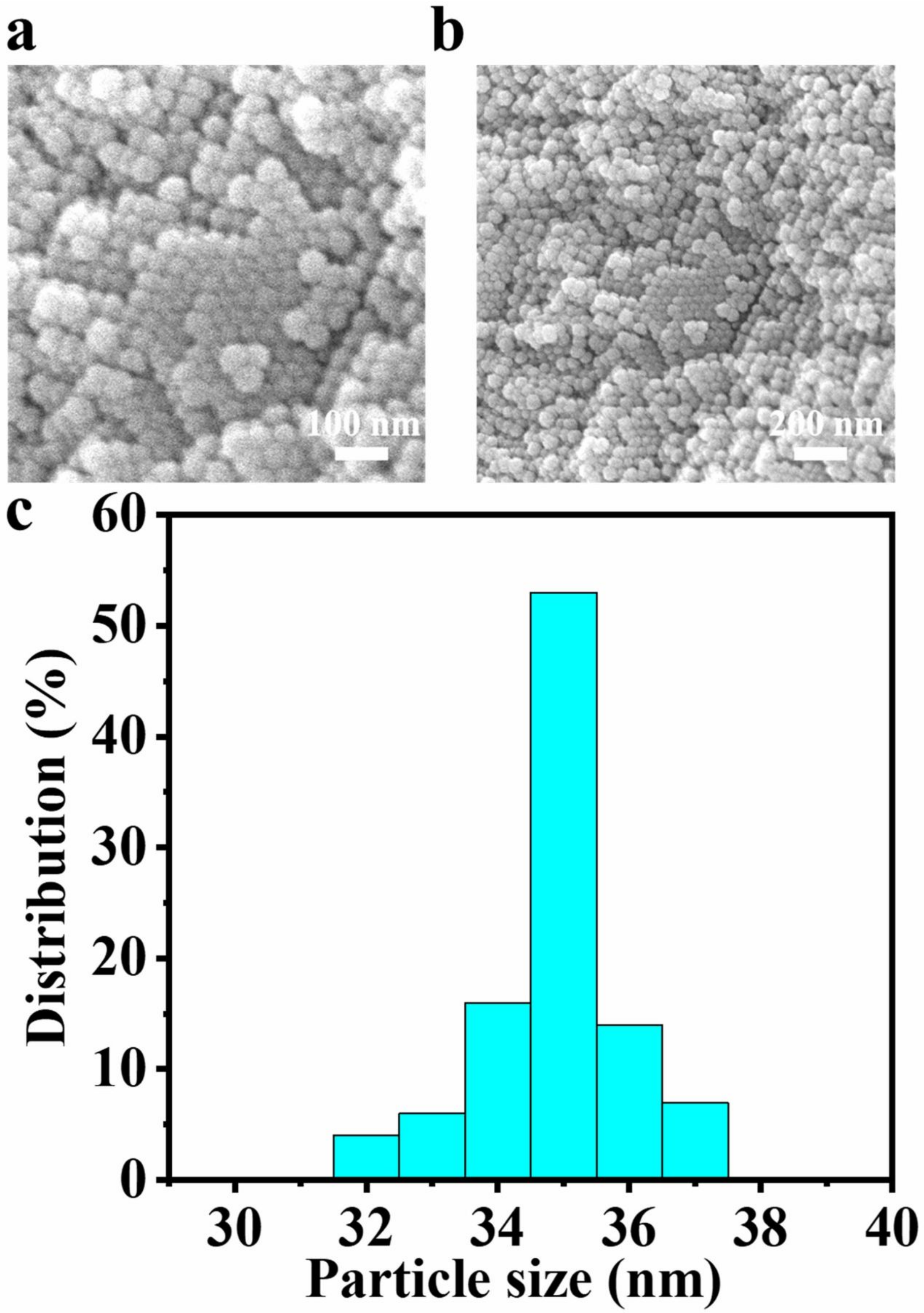

Figure S27. (a, b) SEM images at different magnifications and (c) the corresponding particle size distribution of 3DO-SiO_2(35).

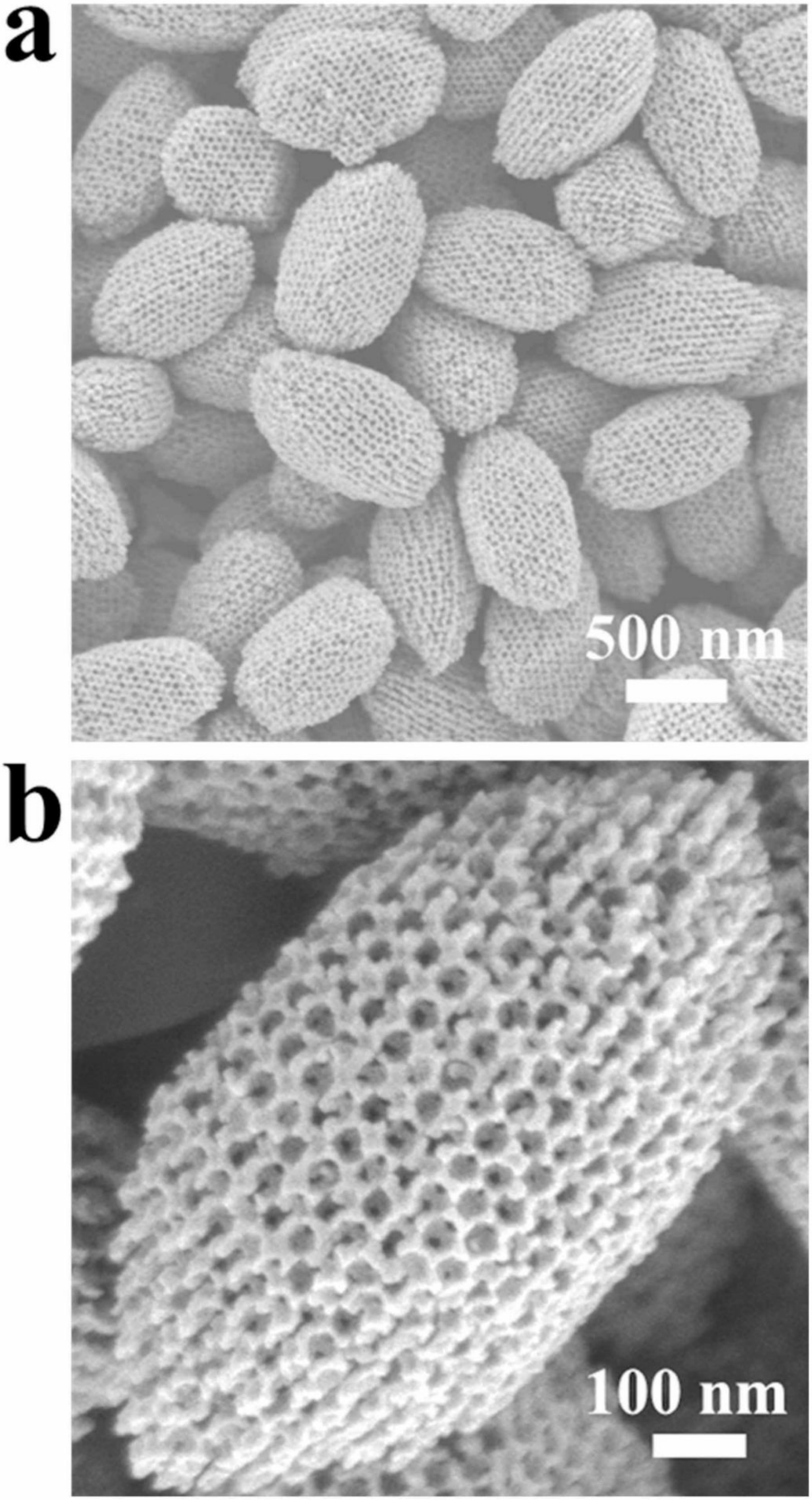

Figure S28. (a, b) SEM images of 3DOm-SnO_2(35) at different magnifications.

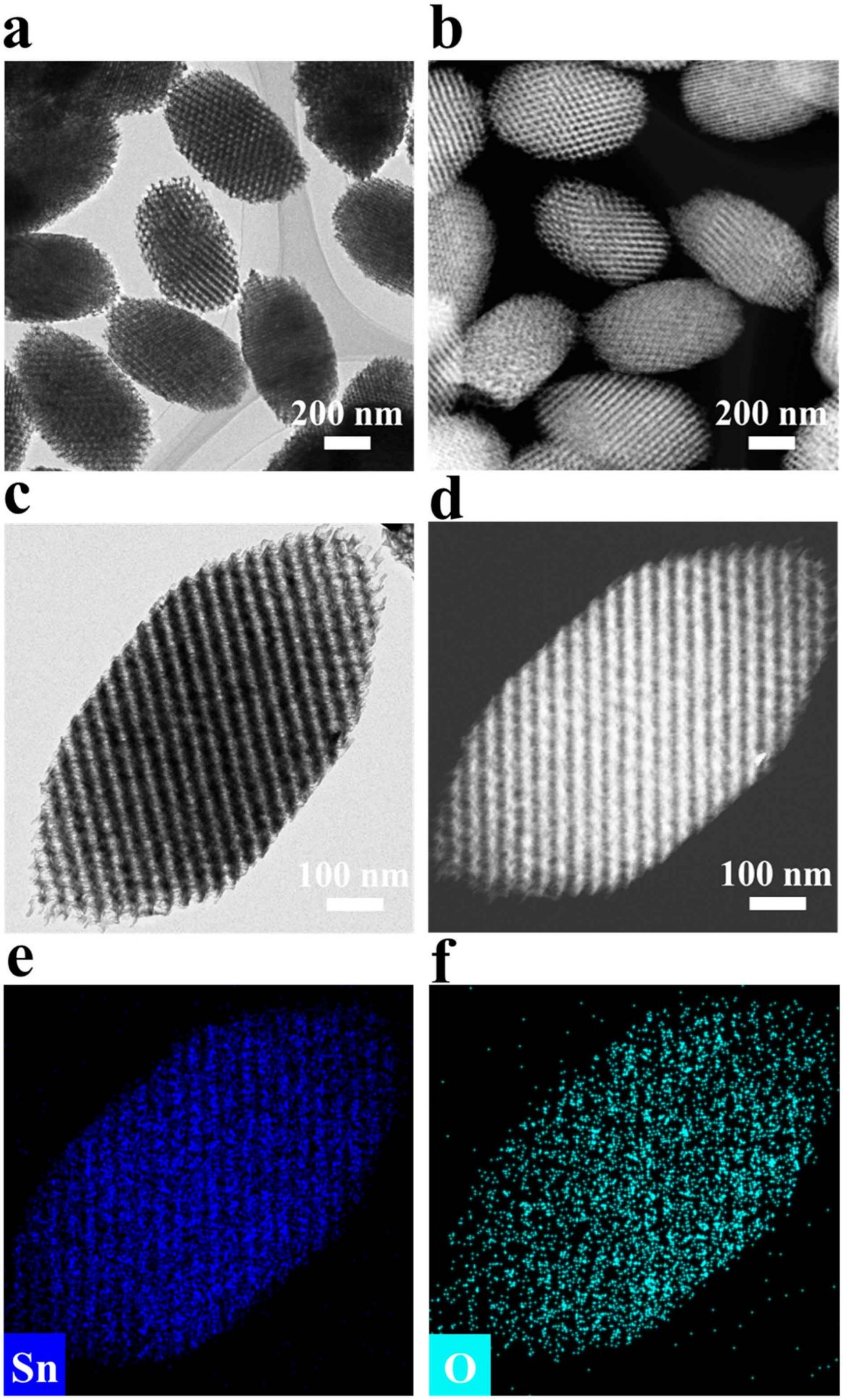

Figure S29. (a, c) TEM and (b, d) STEM images of 3DOm-SnO_2(35) at different magnifications. (e, f) The corresponding EDS mapping images of 3DOm-SnO_2(35).

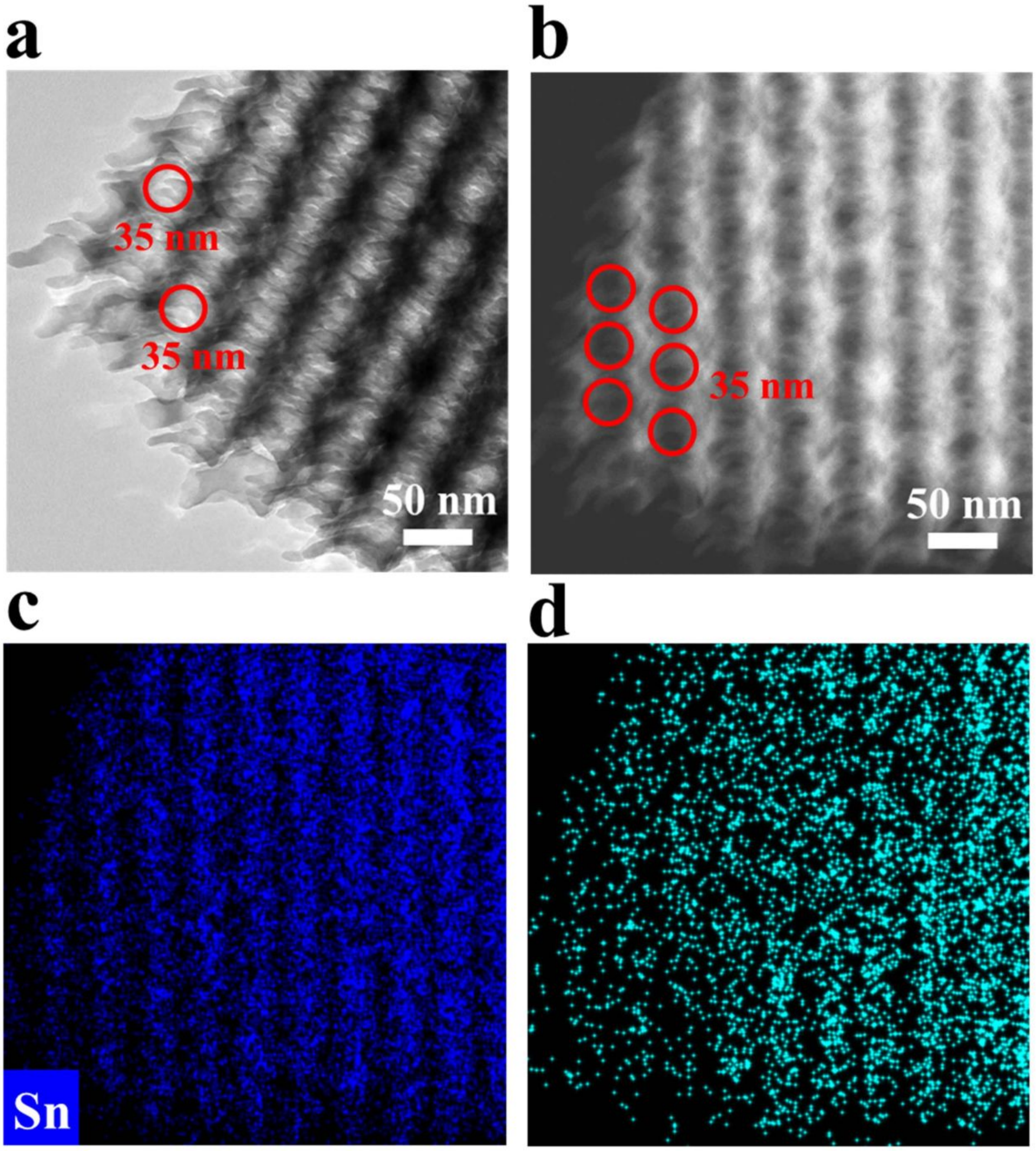

Figure S30. (a) TEM, (b) STEM and (c, d) the corresponding EDS mapping images of 3DOm-SnO_2(35).

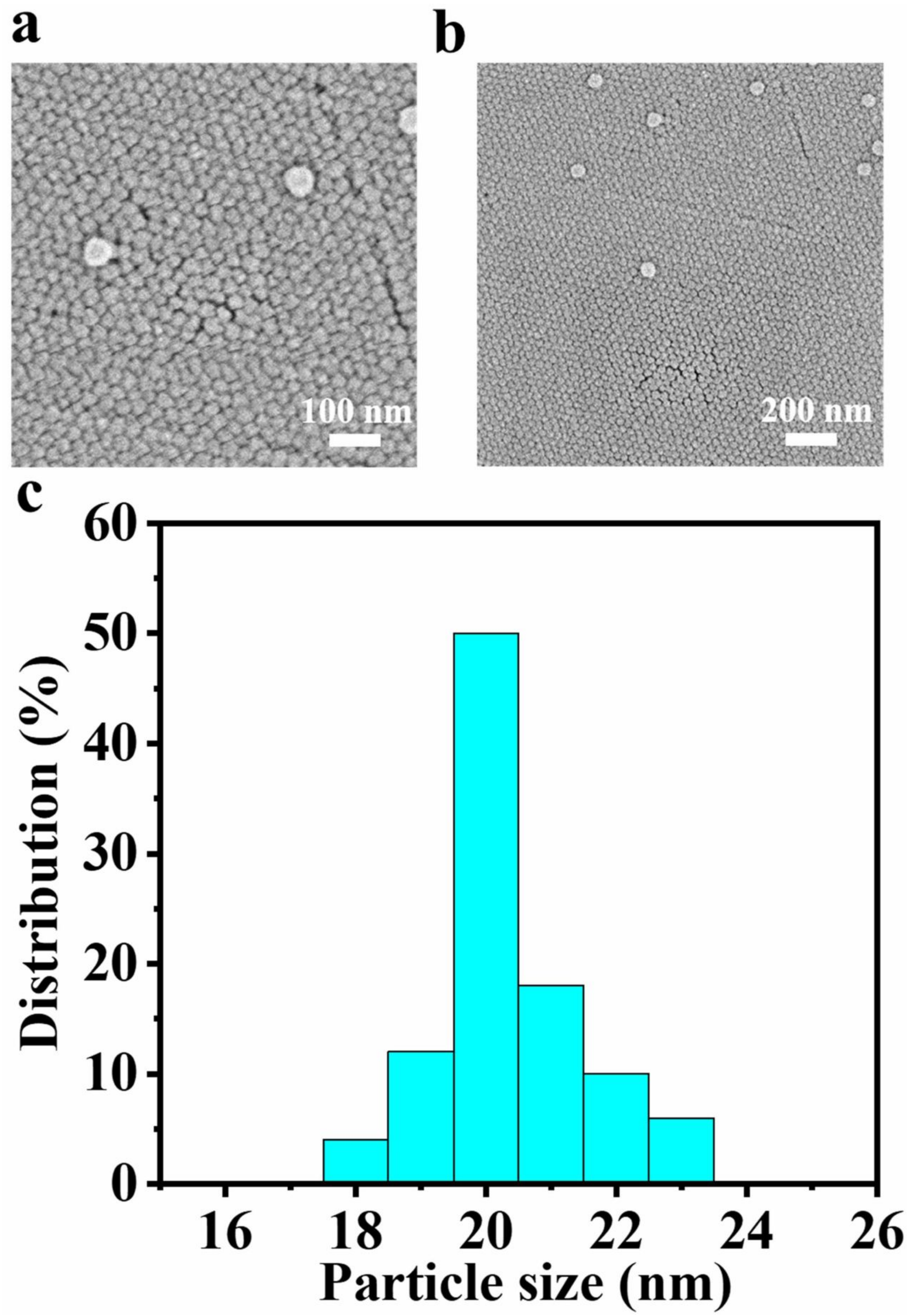

Figure S31. (a, b) SEM images at different magnifications and (c) the corresponding particle size distribution of 3DO-SiO_2(20).

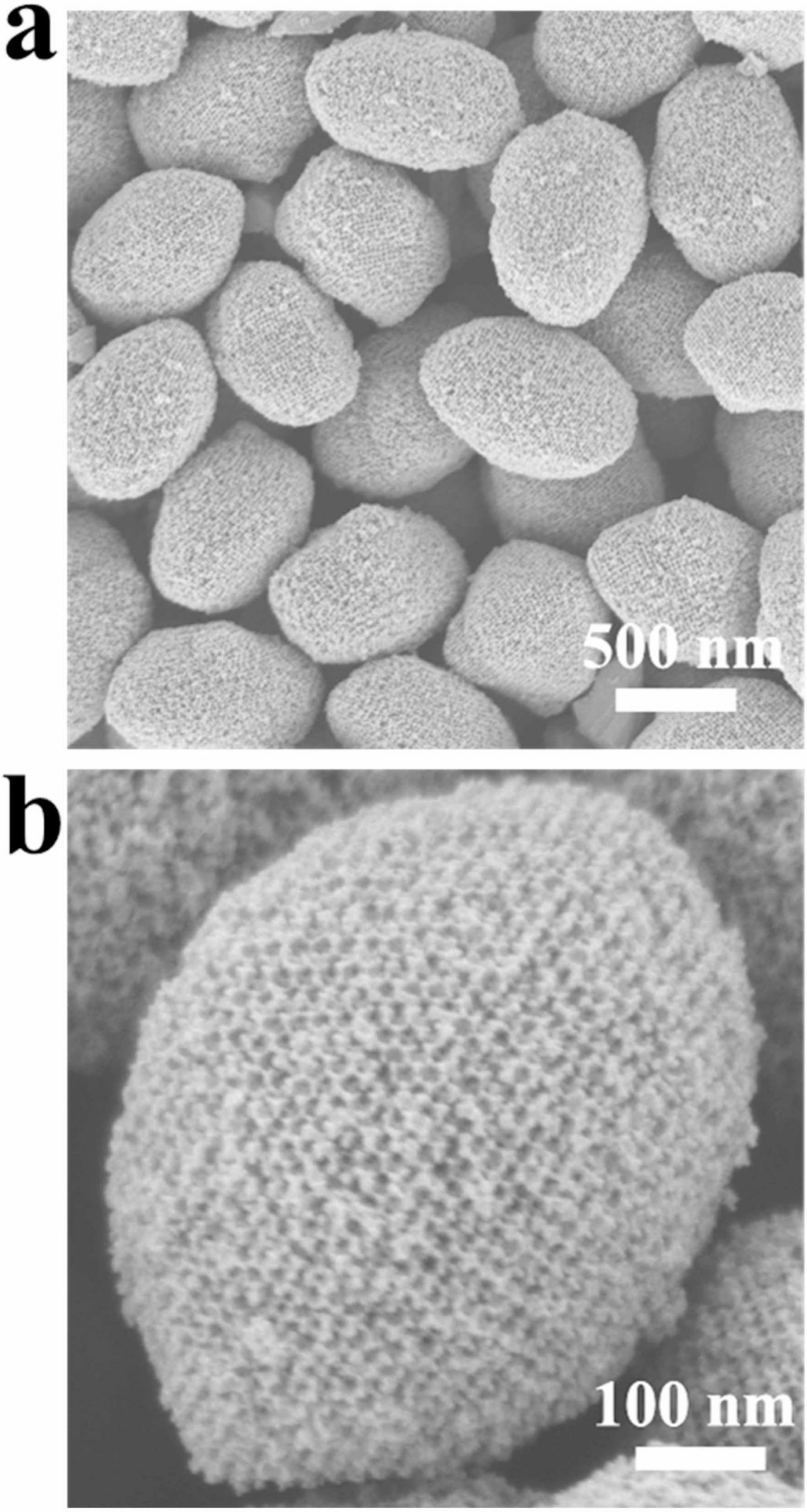

Figure S32. (a, b) SEM images of 3DOm-SnO_2(20) at different magnifications.

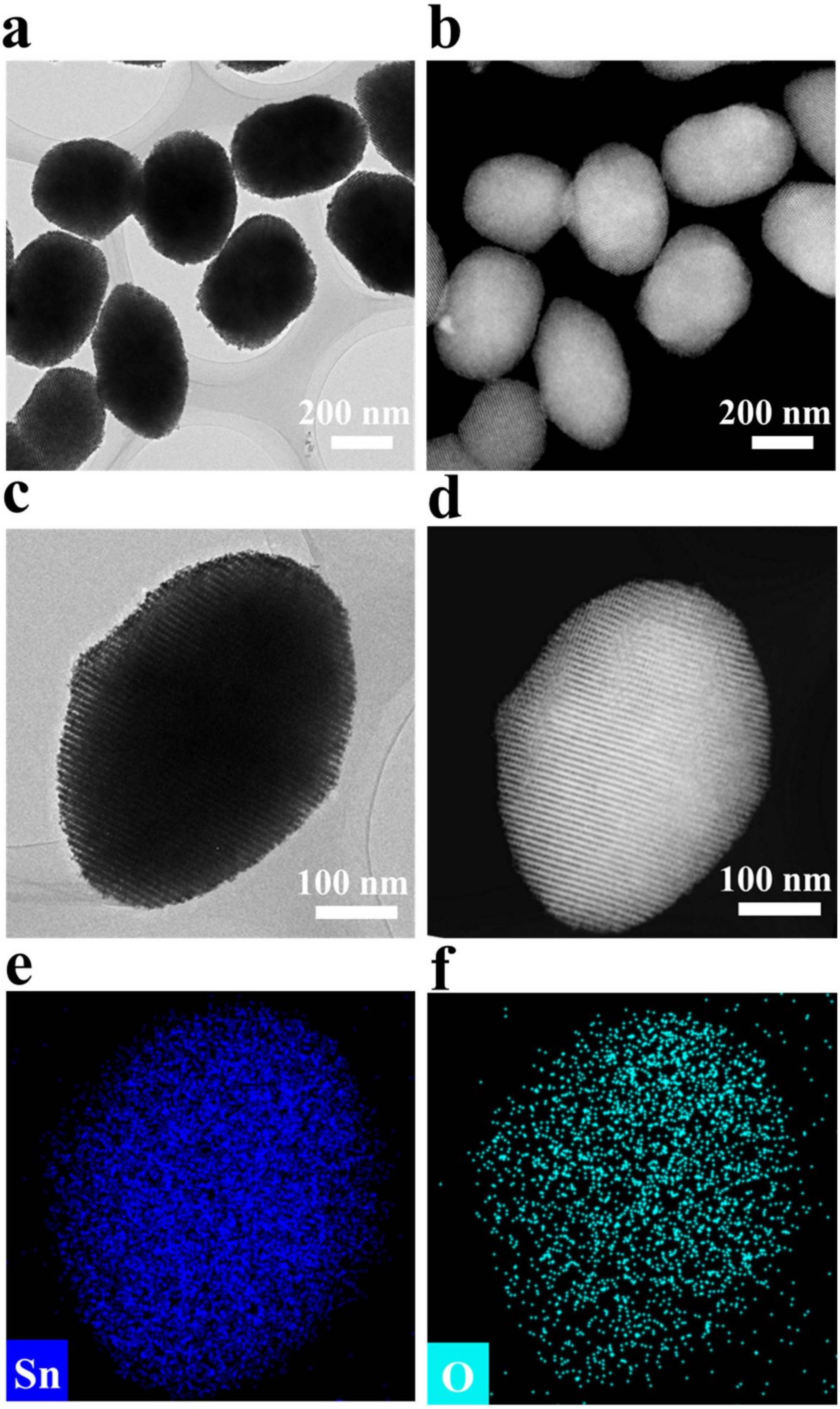

Figure S33. (a, c) TEM and (b, d) STEM images of 3DOm-SnO_2(20) at different magnifications. (e, f) The corresponding EDS mapping images of 3DOm-SnO_2(20).

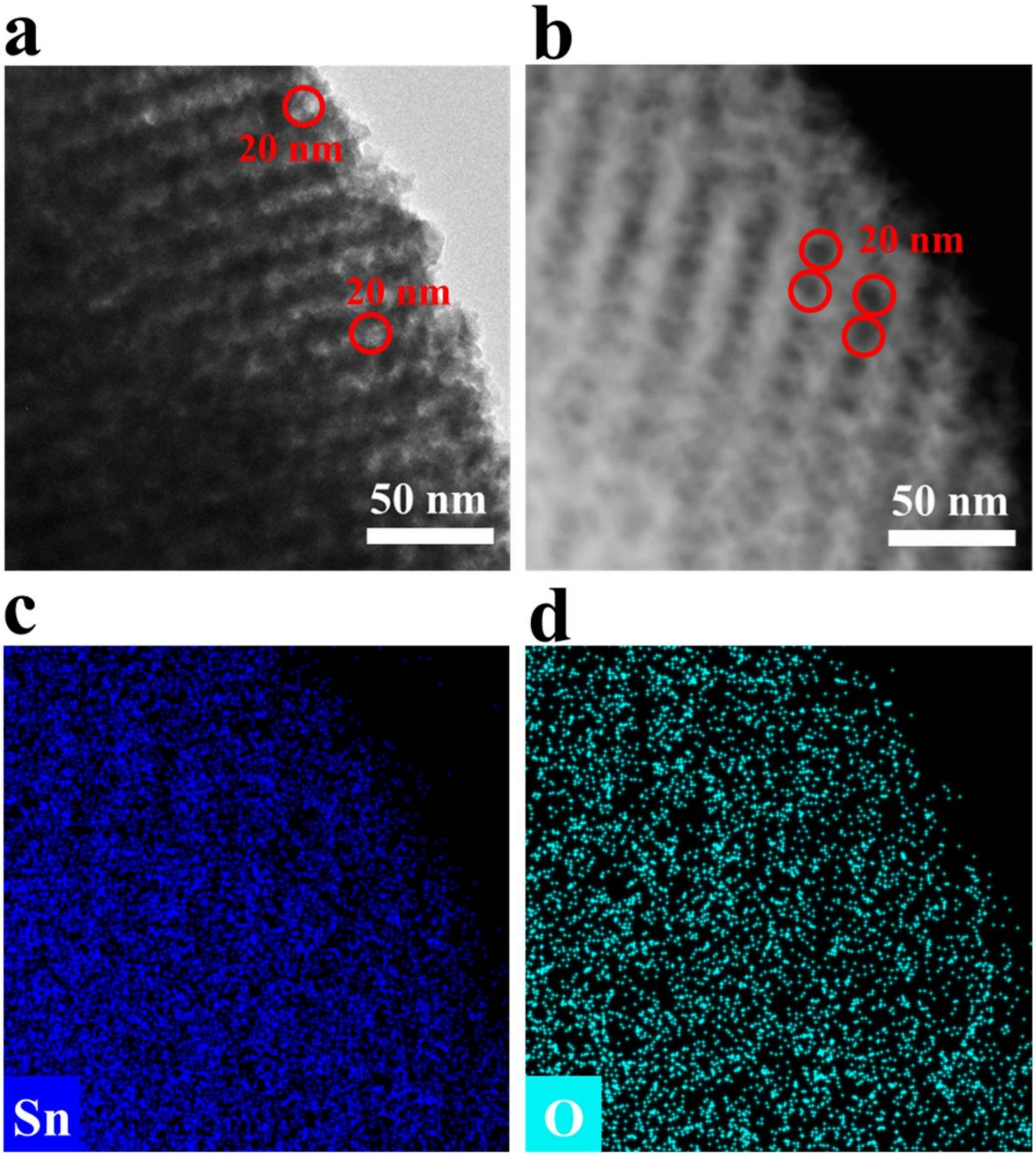

Figure S34. (a) TEM, (b) STEM and (c, d) the corresponding EDS mapping images of 3DOm-SnO_2(20).

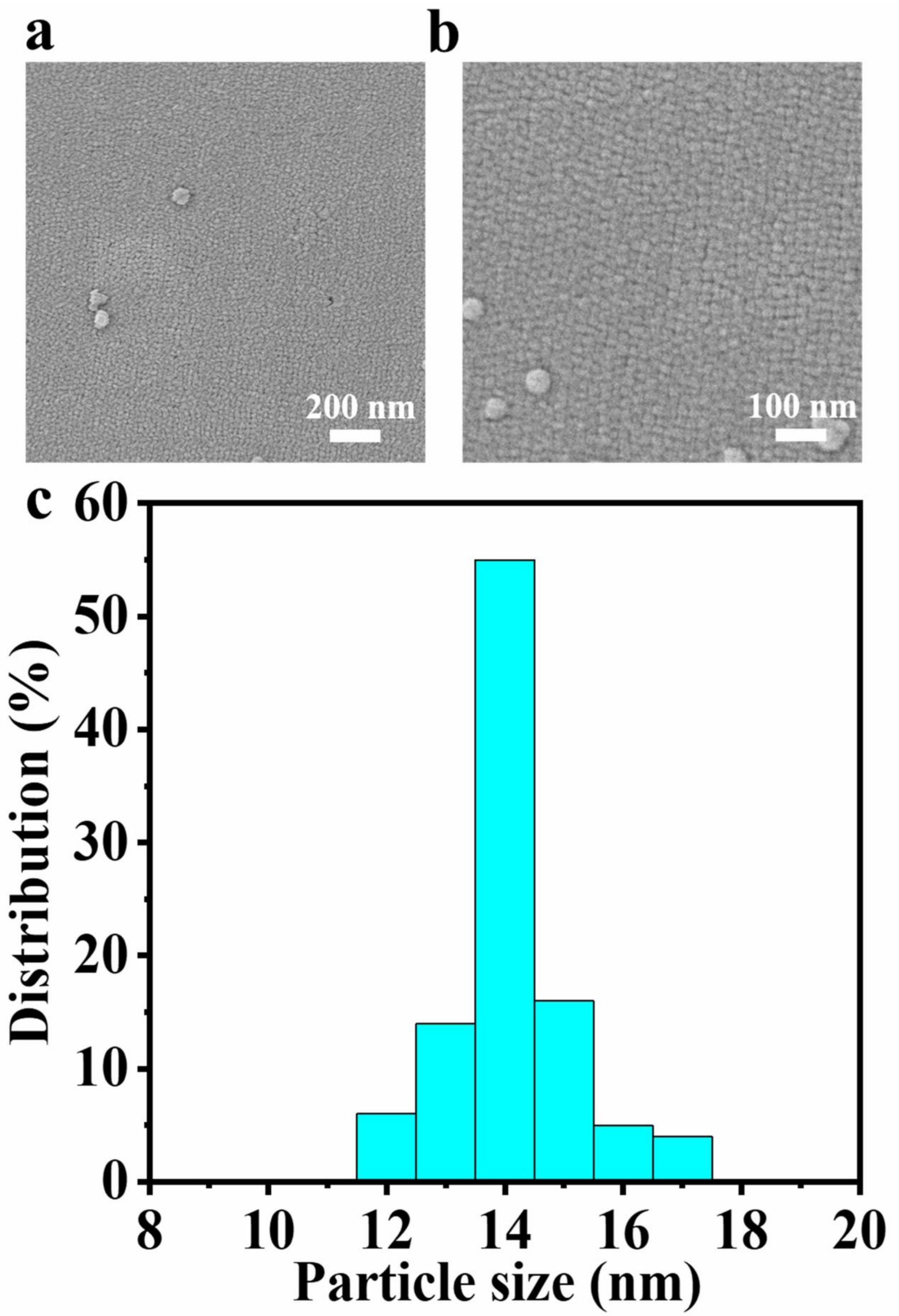

Figure S35. (a, b) SEM images at different magnifications and (c) the corresponding particle size distribution of 3DO-SiO_2(14).

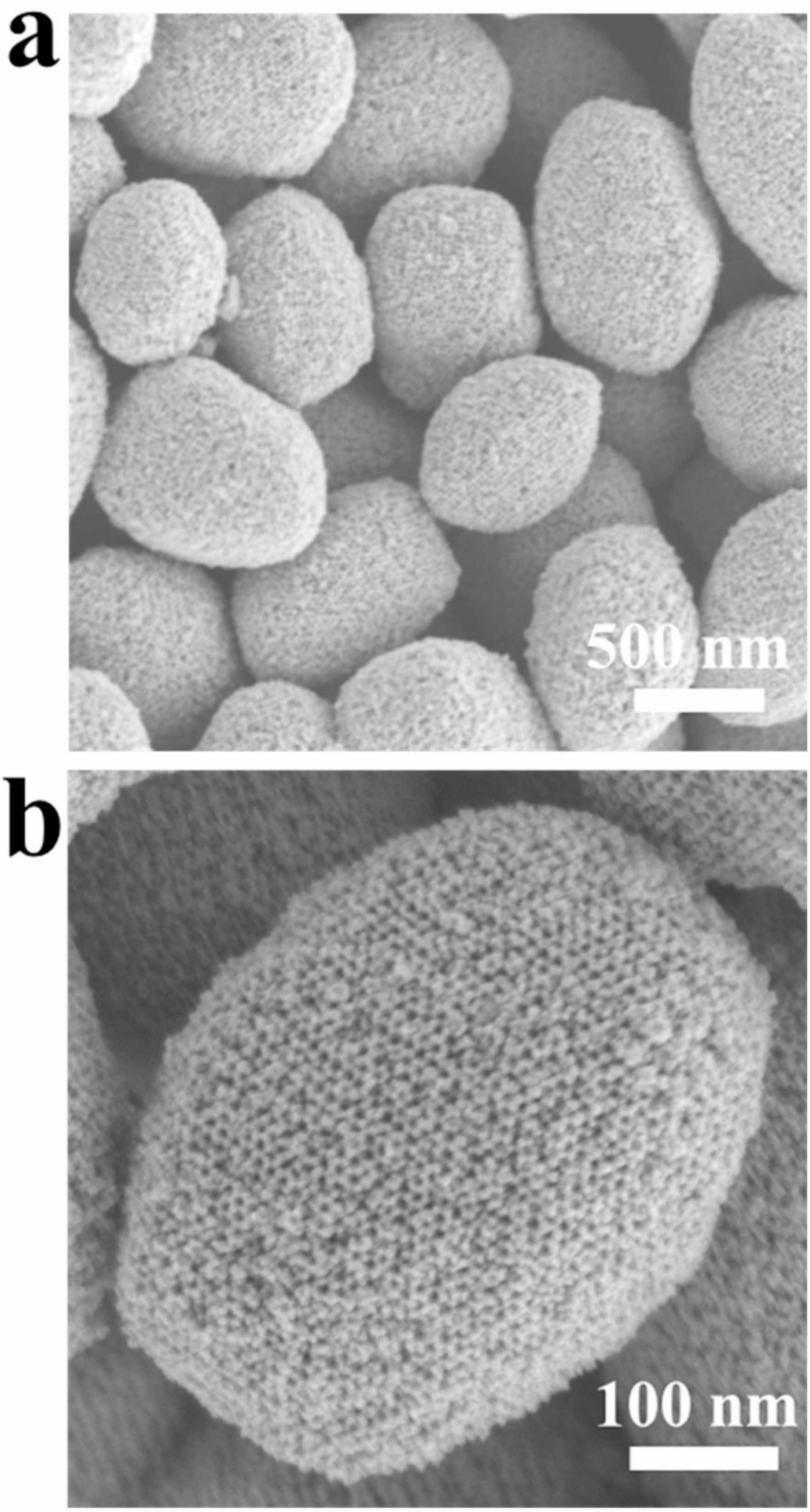

Figure S36. (a, b) SEM images of 3DOm-SnO_2(14) at different magnifications.

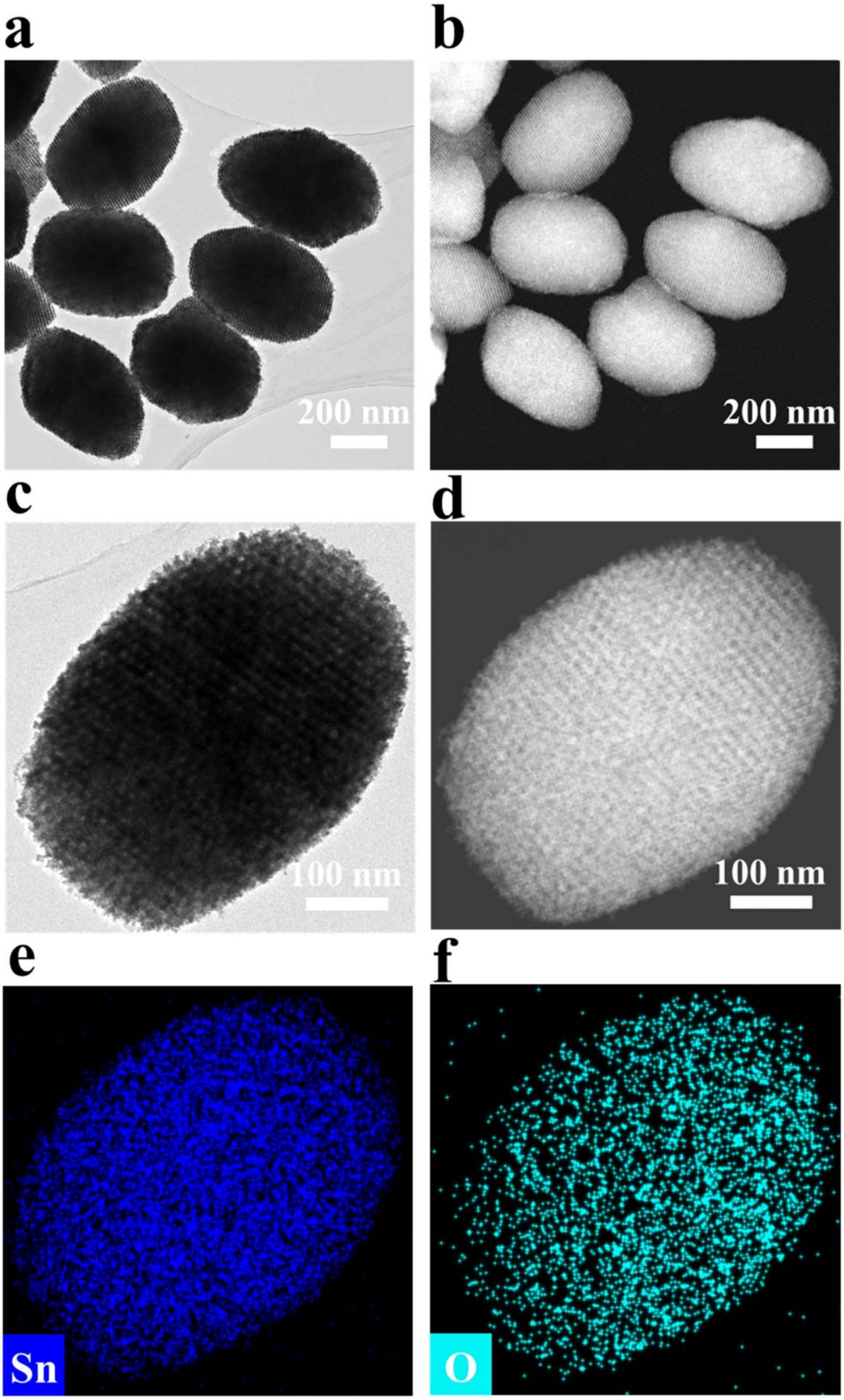

Figure S37. (a, c) TEM and (b, d) STEM images of 3DOm-SnO2(14) at different magnifications. (e, f) The corresponding EDS mapping images of 3DOm-SnO_2(14).

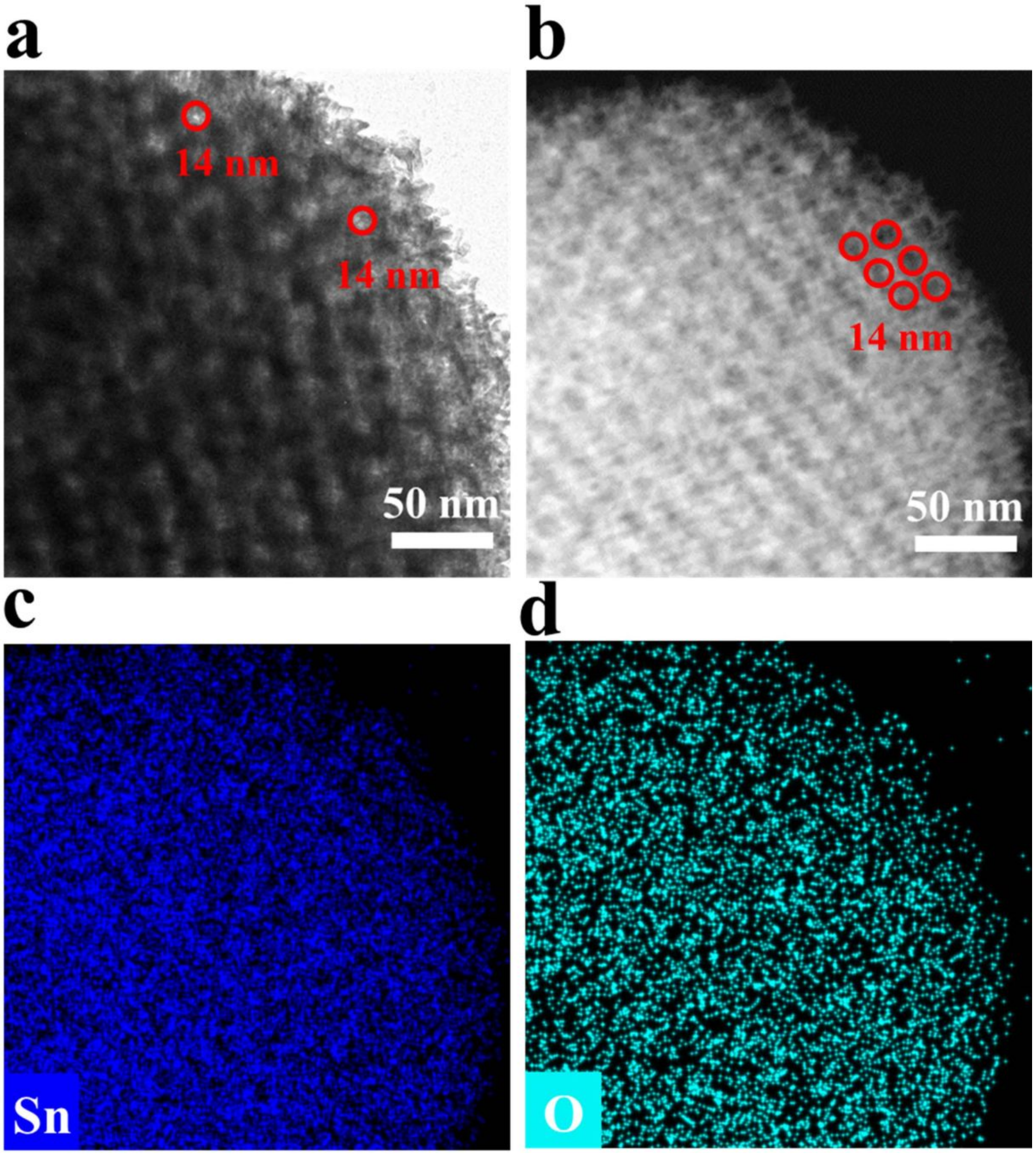

Figure S38. (a) TEM, (b) STEM and (c, d) the corresponding EDS mapping images of 3DOm-SnO_2(14).

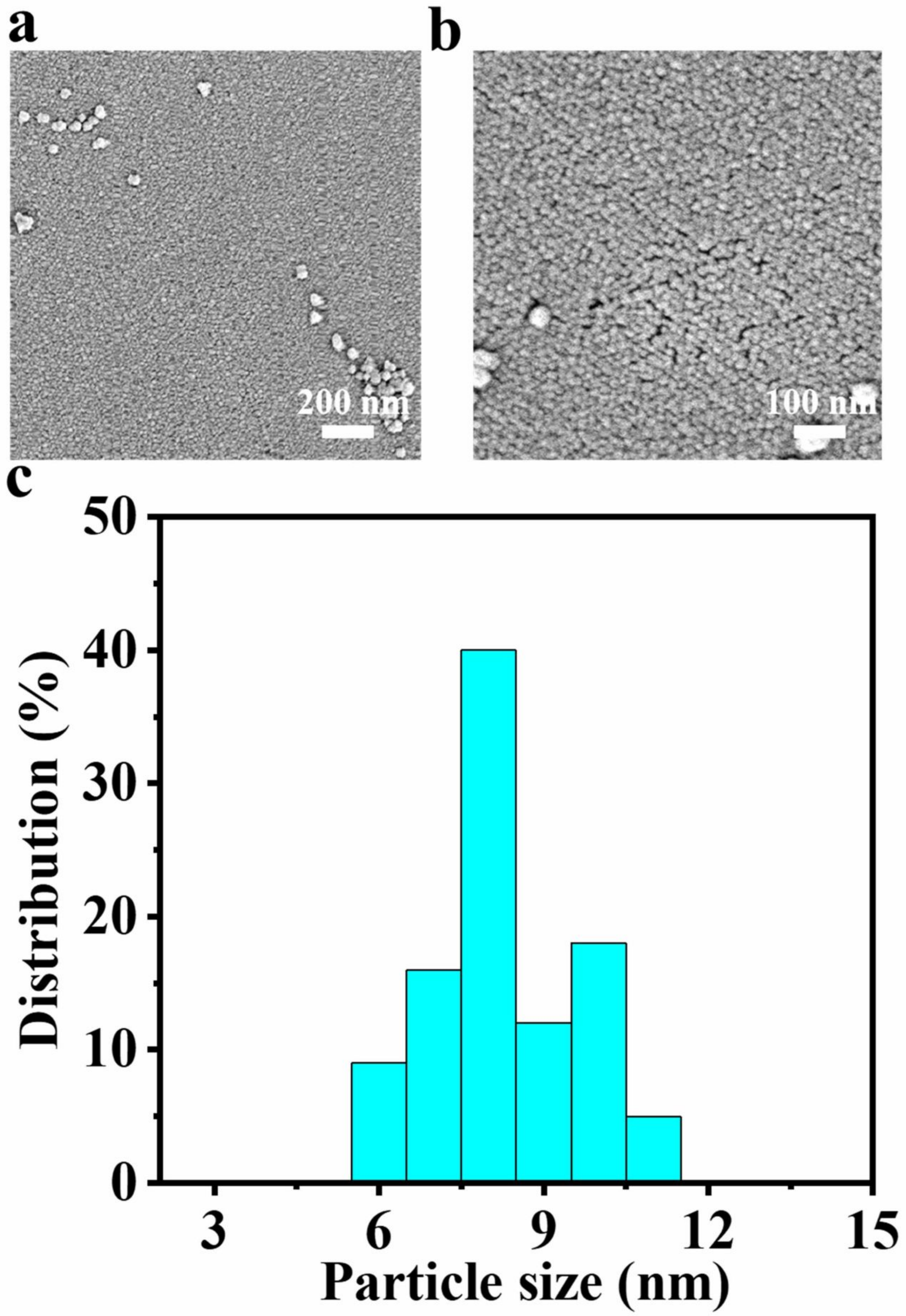

Figure S39. (a, b) SEM images at different magnifications and (c) the corresponding particle size distribution of 3DO-SiO_2(8).

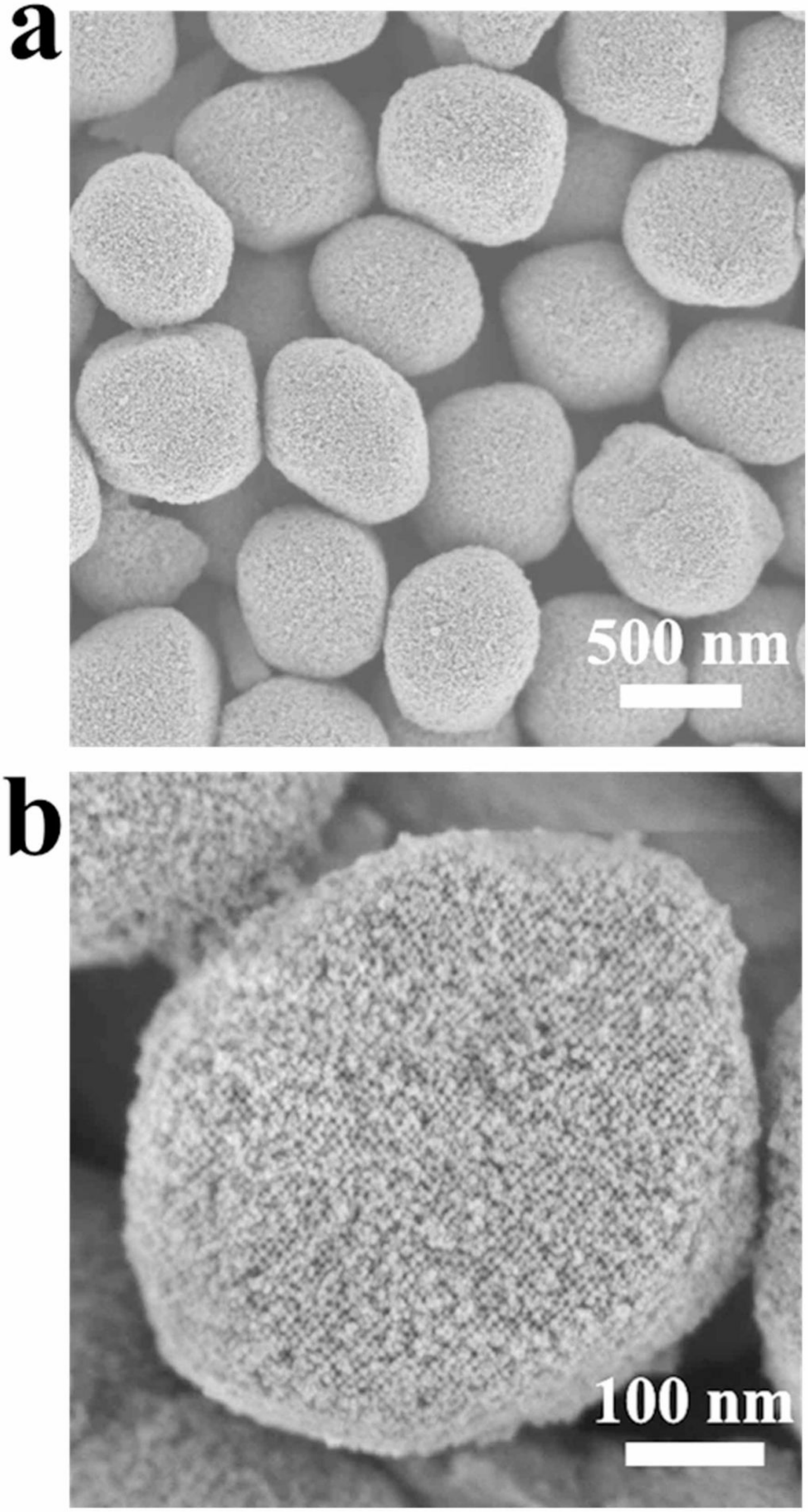

Figure S40. (a, b) SEM images of 3DOm-SnO_2(8) at different magnifications.

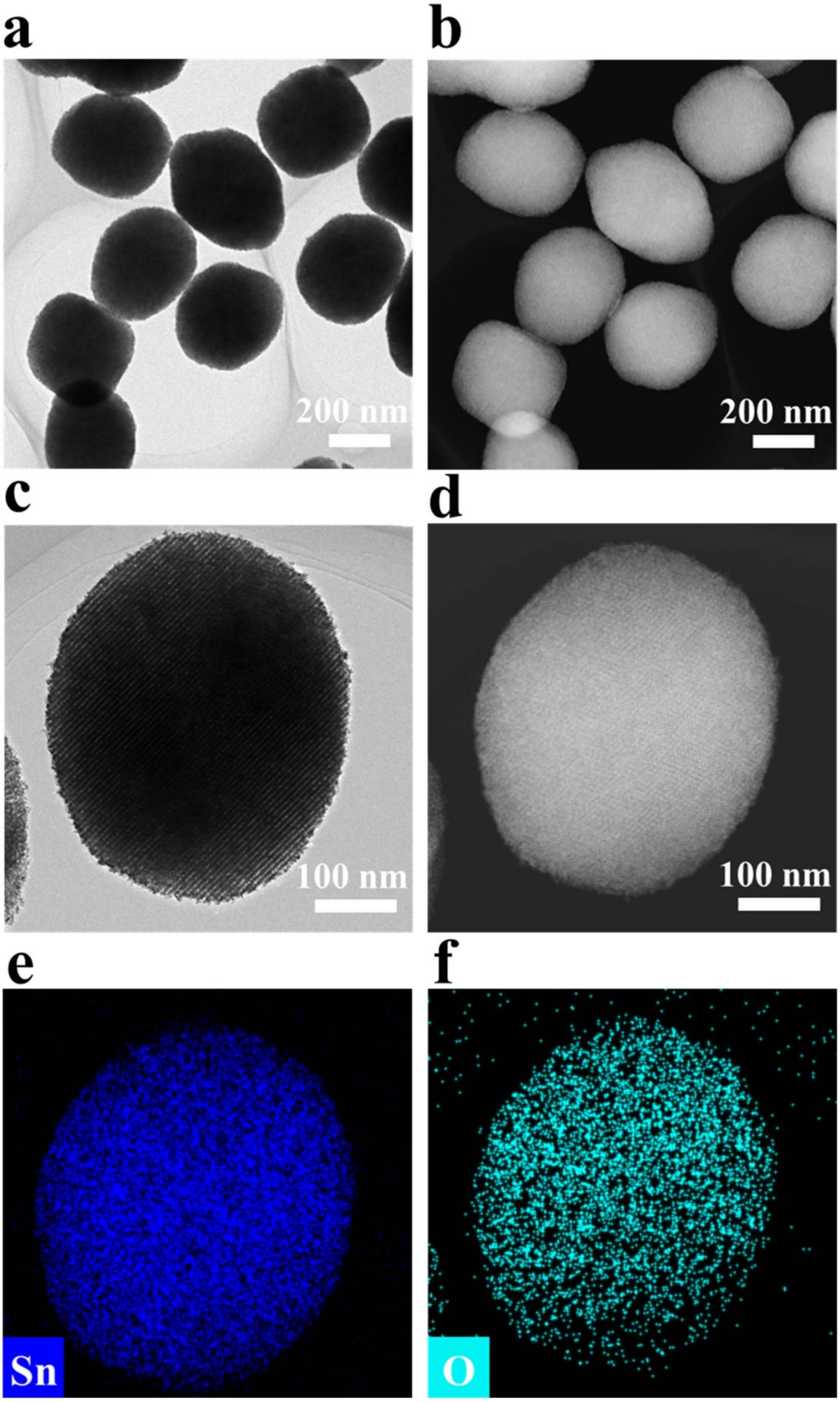

Figure S41. (a, c) TEM and (b, d) STEM images of 3DOm-SnO_2(8) at different magnifications. (e, f) The corresponding EDS mapping images of 3DOm-SnO_2(8).

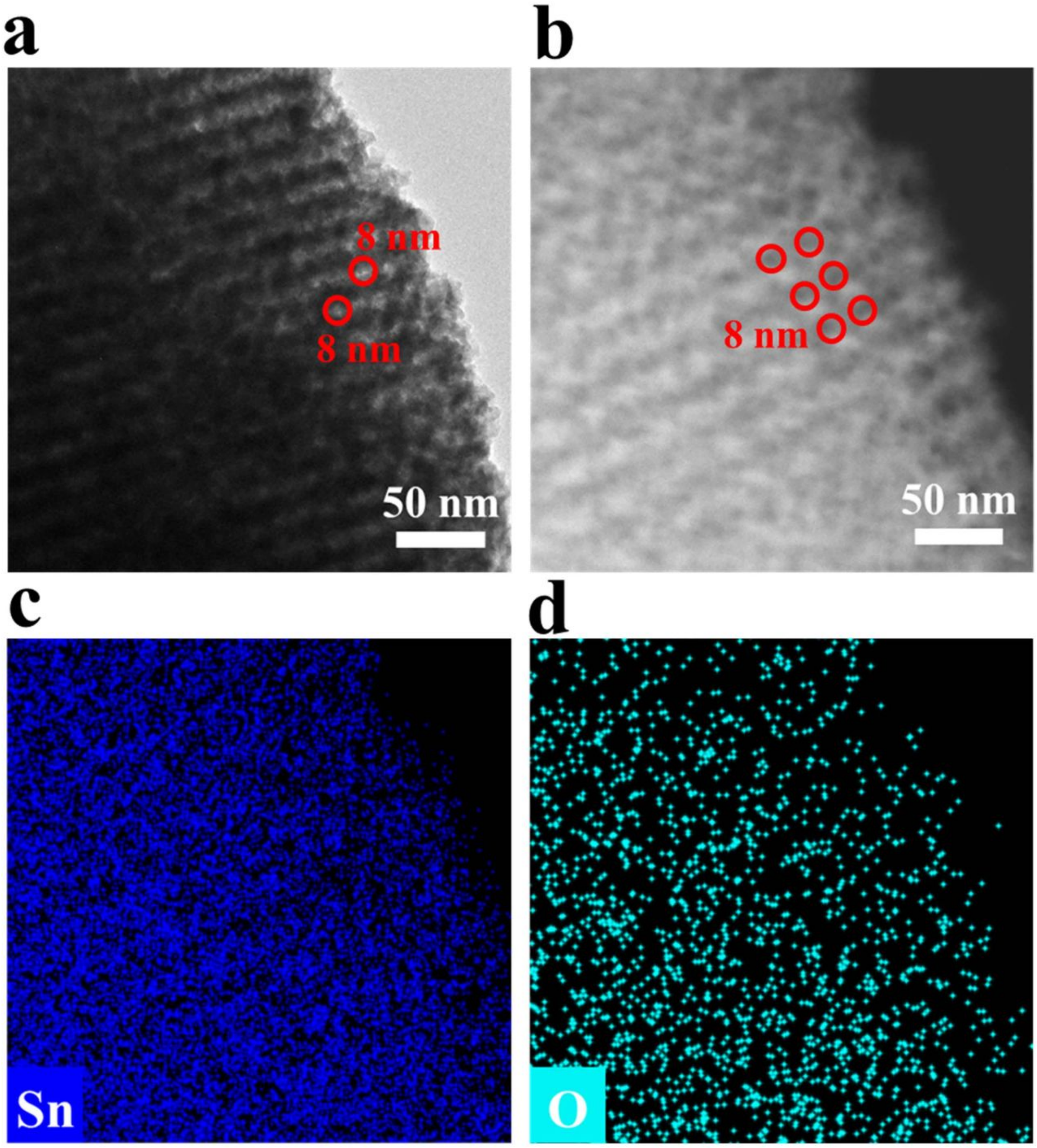

Figure S42. (a) TEM, (b) STEM and (c, d) the corresponding EDS mapping images of 3DOm-SnO_2(8).

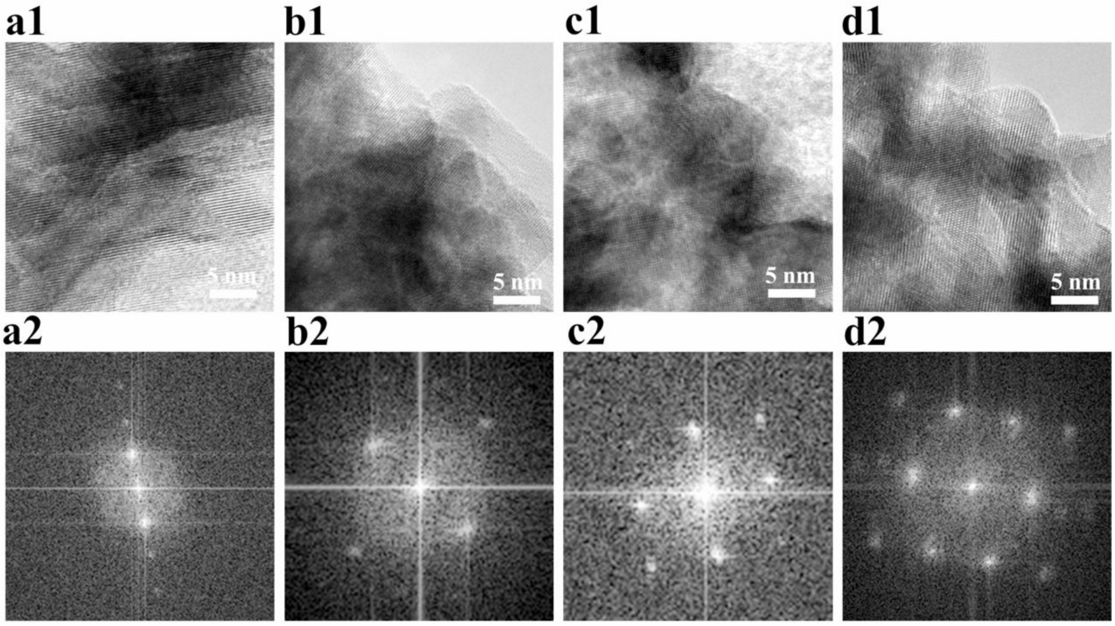

Figure S43. (a1-d1) Lattice-resolution TEM images and (a2-d2) the corresponding FFT patterns of 3DOm-SnO_2(35) (a1, a2), 3DOm-SnO_2(20) (b1, b2), 3DOm-SnO_2(14) (c1, c2) and 3DOm-SnO_2(8) (d1, d2).

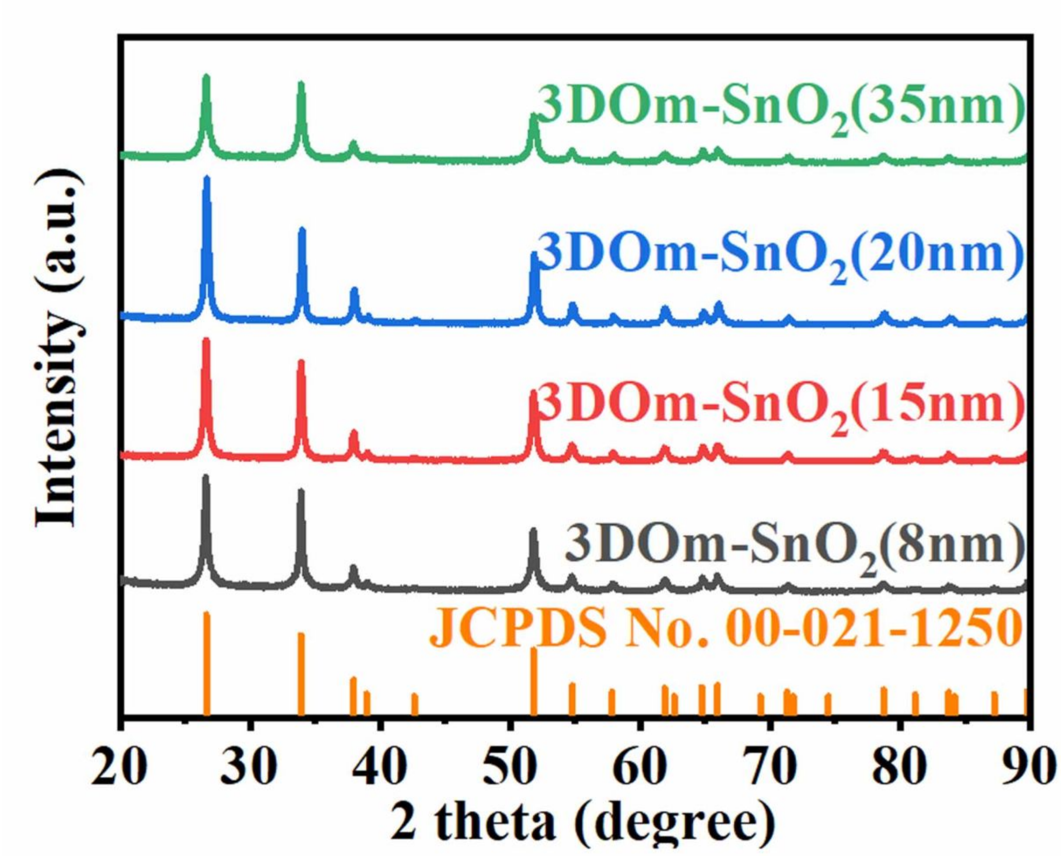

Figure S44. XRD patterns of various 3DOm-SnO_2(S) samples.

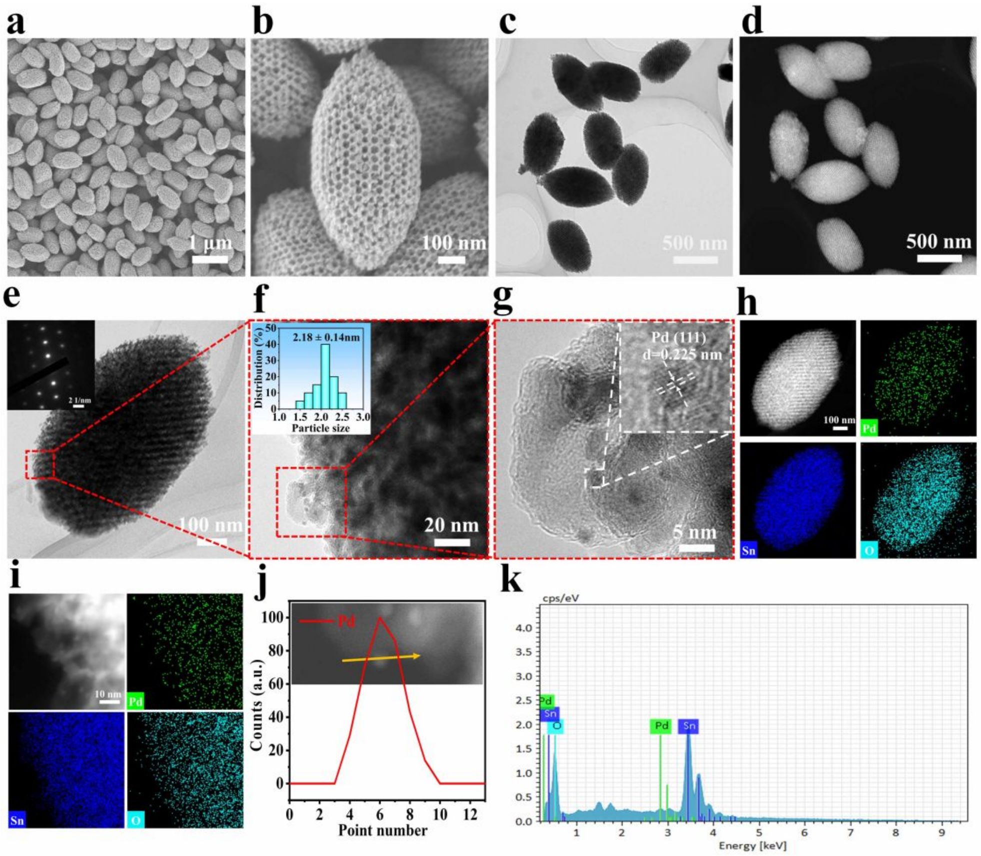

Figure S45. Characterization of Pd/3DOm-SnO_2(27). (a, b) SEM, (c) TEM and (d) STEM of Pd/3DOm-SnO_2(27). (e-h) TEM images of Pd/3DOm-SnO_2(27) taken from the same Pd/3DOm-SnO_2(27) particle at different magnifications. (h, i) STEM and the corresponding EDS mapping images of Pd/3DOm-SnO_2(27) at different magnifications. (j) EDX line-scan profile across a single Pd nanoparticle selected from I and (k) its corresponding elemental spectrum. The inset of e is the corresponding SAED pattern, the inset of f is the corresponding particle size distribution histogram, and the inset of g is the HRTEM image of an individual Pd nanoparticle.

The SEM and TEM images reveal that the olivary morphology and 3DOm structure of 3DOm-SnO_2 can be well preserved after the loading of Pd NPs, and the 3DOm-SnO_2 support still possesses the single-crystalline property. A closer observation on the high-magnification TEM image of Pd/3DOm-SnO_2 reveals that the Pd NPs are homogeneously distributed on Pd/3DOm-SnO_2(27) with a mean diameter of 2.18 ± 0.14 nm, where no remarkable formation of aggregates can be observed. The corresponding HRTEM image shows the well-resolved lattice fringes with an interplane distance of ~ 0.225 nm, which can be attributed to the (111) plane of the Pd NPs with a face-centered cubic (fcc) structure. Subsequently, the representative STEM and the corresponding EDS mapping images show that the uniform distributions of Pd, Sn and O elements are observed in the same area, further confirming that Pd NPs are effectively anchored on the 3DOm-SnO_2(27) matrix with potential interaction between Pd NPs and the support.

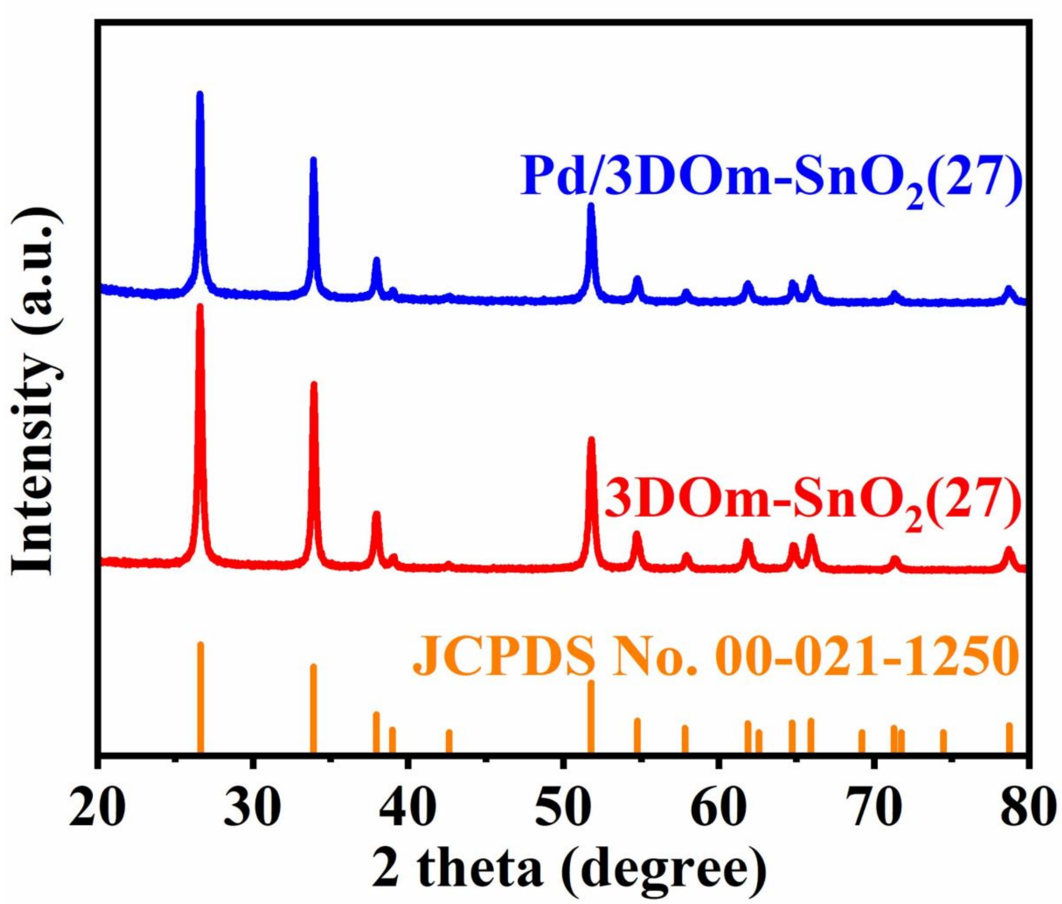

Figure S46. PXRD patterns of Pd/3DOm-SnO_2(27) and 3DOm-SnO_2(27). It is obvious that the PXRD pattern of Pd/3DOm-SnO_2(27) matches well with that of the as-synthesized 3DOm-SnO_2(27), indicating that the crystalline structure of 3DOm-SnO_2(27) can be well retained after loading Pd NPs. In addition, the diffraction peaks of Pd NPs do not appear, which is due to the low loading or/and ultrafine particle size of Pd NPs in Pd/3DOm-SnO_2(27).

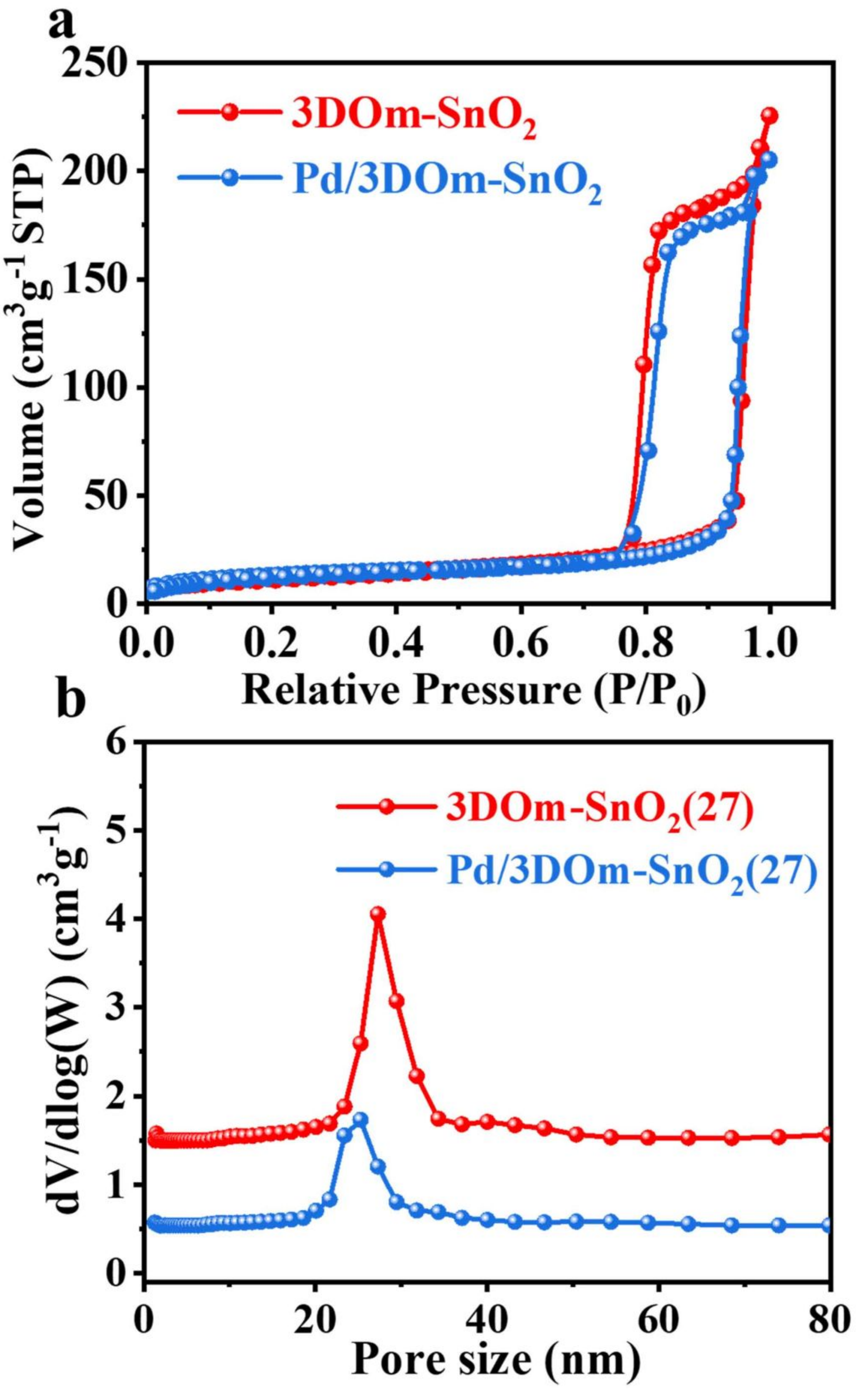

Figure S47. (a) N_2 adsorption/desorption isotherms and (b) the corresponding pore distributions of Pd/3DOm-SnO_2(27) and 3DOm-SnO_2(27) (based on DFT model by using the desorption branches of their isotherms).

As expected, the both samples exhibit similar type IV isotherms, further indicating the ordered porous structure of Pd/3DOm-SnO_2(27) is well reserved. However, the BET surface area (62 m^2/g), total pore volume (0.40 cm^3/g) and pore diameter (mean size=26.0 nm) of Pd/3DOm-SnO_2(27) are slightly diminished as compared with those of its parental 3DOm-SnO_2(27) (Table S2), which are attributed to the loadings and pore occupation of the immobilized Pd NPs.

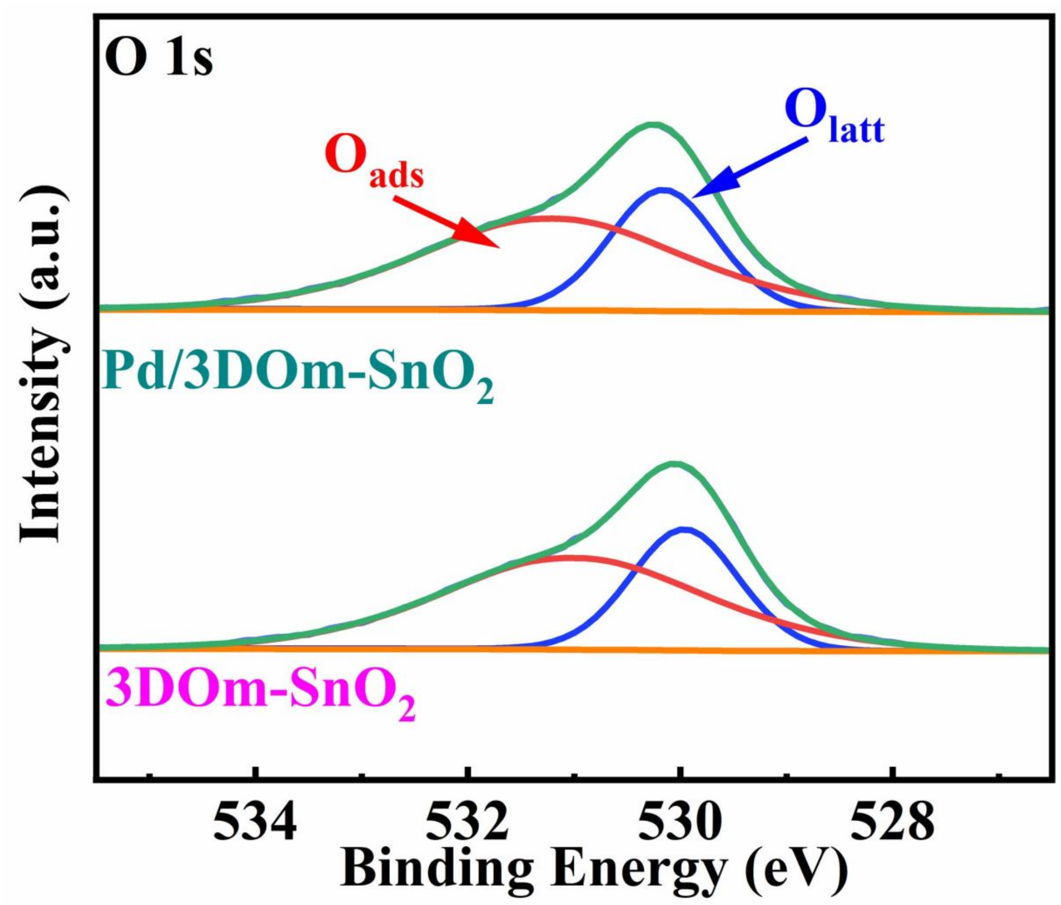

Figure S48. O 1s XPS spectra of Pd/3DOm-SnO_2(27) and 3DOm-SnO_2(27). The O_{ads} / (O_{ads} + O_{latt}) ratio of Pd/3DOm-SnO_2 (60.32%) is similar to that of 3DOm-SnO_2 (64.57%) (Table S3), indicating that the oxygen vacancies of 3DOm-SnO_2 do not alter significantly after loading Pd NPs.

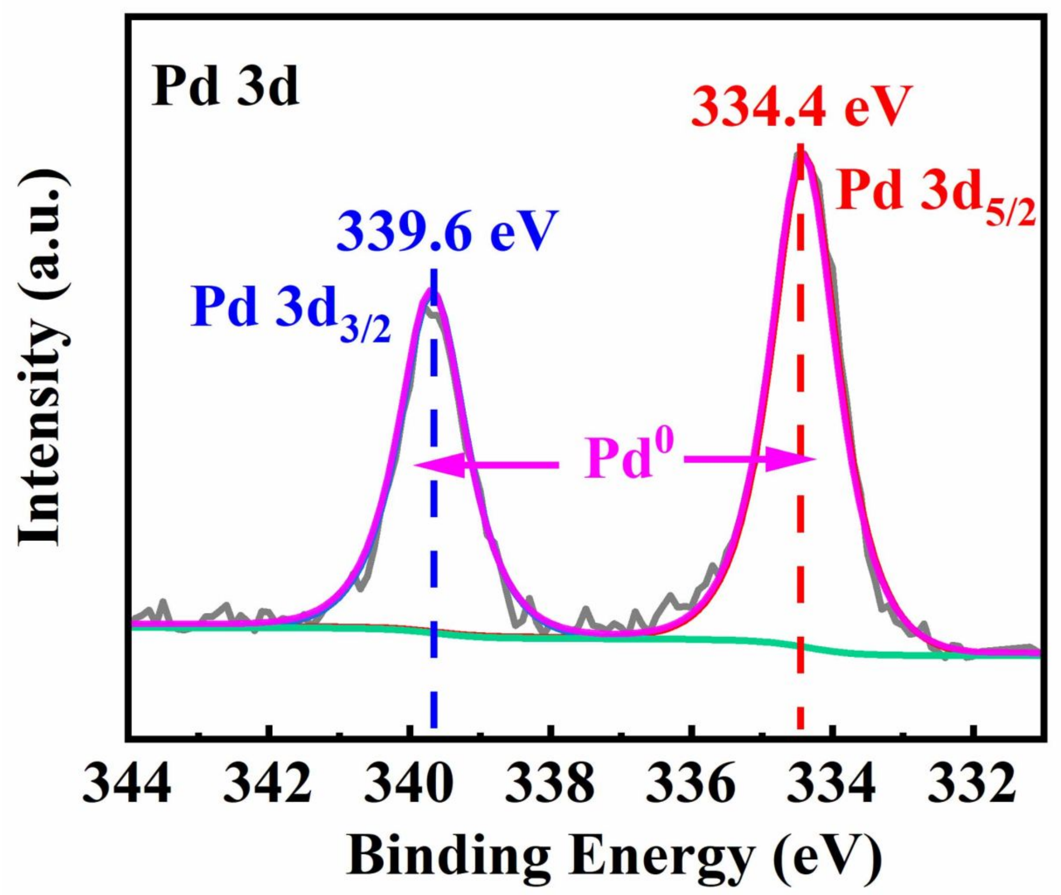

Figure S49. Pd 3d XPS spectrum of Pd/3DOm-SnO_2(27), which shows two obvious peaks at 334.4 eV and 339.6 eV, being ascribed to the binding energies of Pd $3d_{5/2}$ and Pd $3d_{3/2}$ of zerovalent Pd, respectively, indicating that Pd in this sample is in its metallic state.

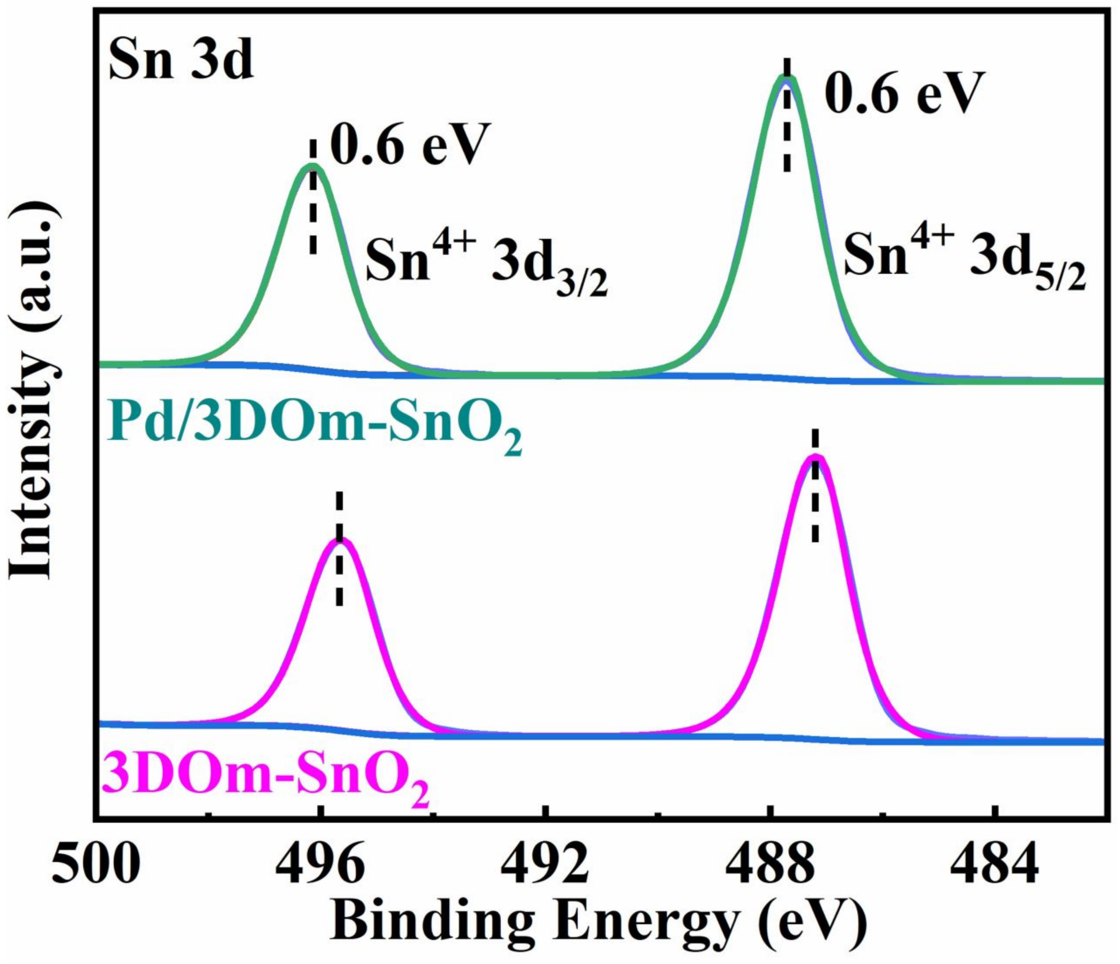

Figure S50. Sn 3d XPS spectra of Pd/3DOm-SnO_2(27) and 3DOm-SnO_2(27). The Sn 3d XPS peaks of Pd/3DOm-SnO_2(27) exhibit an obvious positive shift of ~0.6 eV as compared with those of 3DOm-SnO_2(27), which indicates that electrons are transferred from SnO_2 support to Pd NPs, resulting in the accumulation of electrons on Pd NPs to bring some particular catalytic properties.

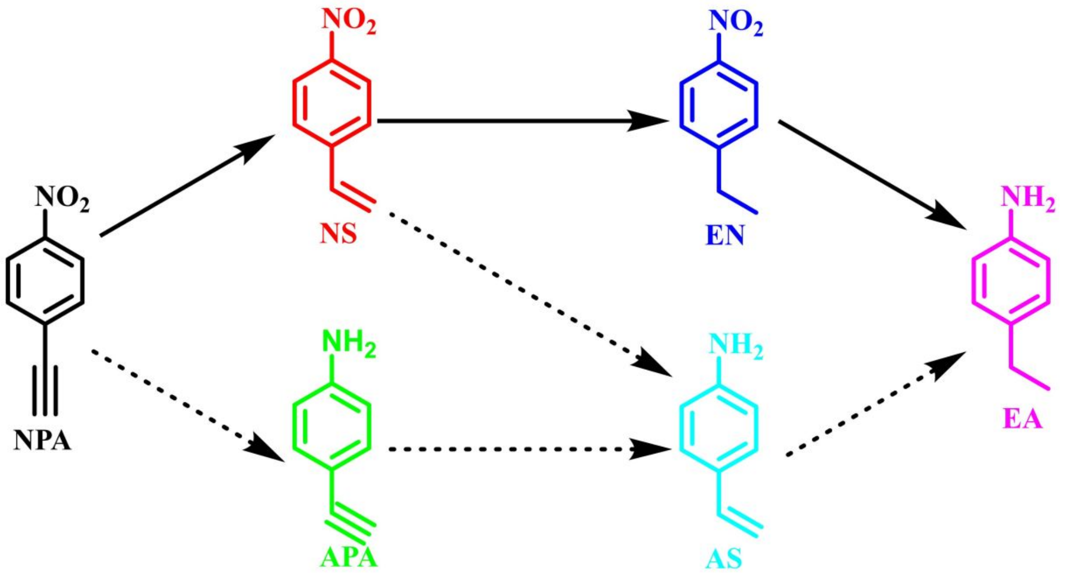

Figure S51. Possible hydrogenation routes and products of NPA. There are two different routes for the hydrogenation of NPA. One is the hydrogenation of nitryl group first, and the other is the hydrogenation of alkynyl (vinyl) groups first.

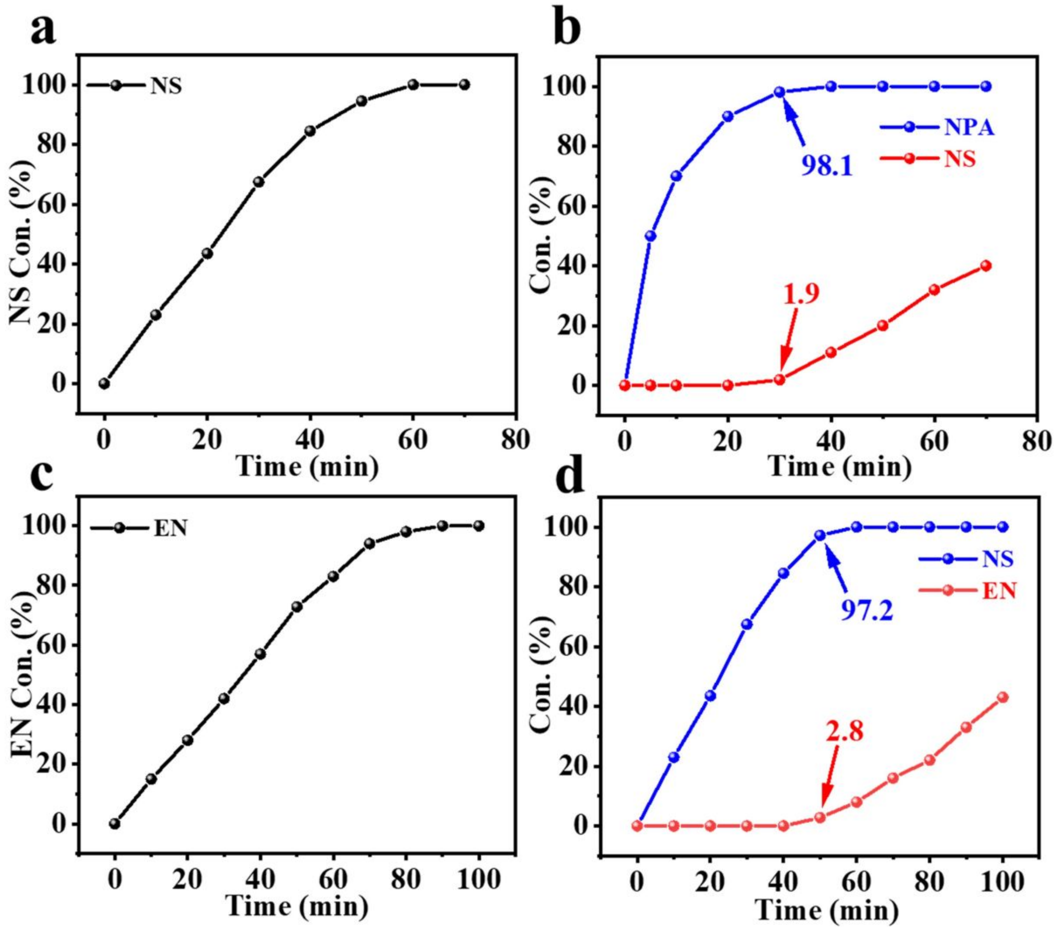

Figure S52. (a) Conversion of NS as a function of reaction time on Pd/3DOm-SnO_2(27). (b) Conversion of NS and NPA as a function of reaction time on Pd/3DOm-SnO_2(27) in presence of 1 mmol NPA. (c) Conversion of EN as a function of reaction time on Pd/3DOm-SnO_2(27). (d) Conversions of EN and NS as a function of reaction time on Pd/3DOm-SnO_2(27) in presence of 1mmol NS. Reaction conditions[a]: NS (1.0 mmol), catalyst (0.25 mol% of Pd), 1,4-dioxane (4 mL), hydrogen (1 bar), room temperature, 70 min. Reaction conditions[b]: the reaction condition is the same as that of above a except with adding additional 1mmol NPA. Reaction conditions[c]: EN (1.0 mmol), catalyst (0.25 mol% of Pd), 1,4-dioxane (4 mL), hydrogen (1 bar), room temperature, 100 min. Reaction conditions[d]: the reaction condition is the same as that of above c except with adding additional 1mmol NS.

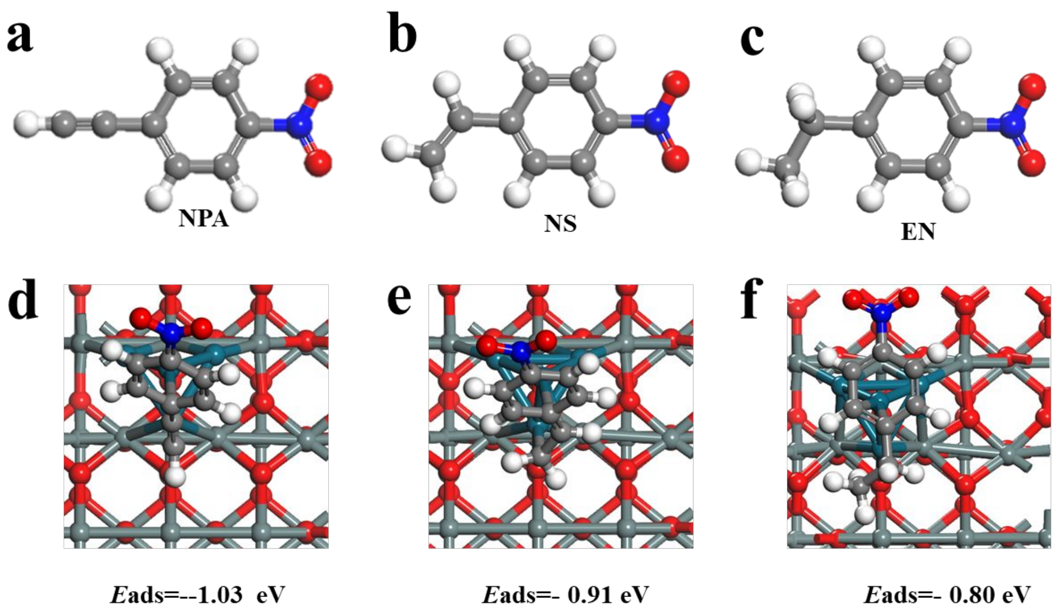

Figure S53. Molecular configuration models of NPA (a), NS (b) and EN (c), and their adsorption structures and adsorption free energies on the Pd_4/SnO_2 (d-f).

Density functional theory calculations are performed to further investigate the adsorption of NPA, NS and EN on Pd NPs of Pd/3DOm-SnO_2. Pd_4/SnO_2 is used as a model to carry out the calculation. The adsorption free energies of NPA, NS and EN on the Pd_4/SnO_2 are -1.03 eV, -0.91 eV and -0.80 eV, respectively. These results indicate that the binding interaction intensity order of NPA, NS and EN on Pd_4/SnO_2 is NPA>NS>EN. Namely, NPA can be preferentially adsorbed on Pd NPs of Pd/3DOm-SnO_2(27) to impede the hydrogenation of NS, and only when the hydrogenation of NPA is basically complete, NS tends to adsorb on Pd NPs for further hydrogenation. Similarly, when the hydrogenation of NS is basically complete to expose Pd NPs, EN begins to adsorb on them for subsequent reaction. Therefore, the sequential hydrogenation of NPA is observed on Pd/3DOm-SnO_2(27).

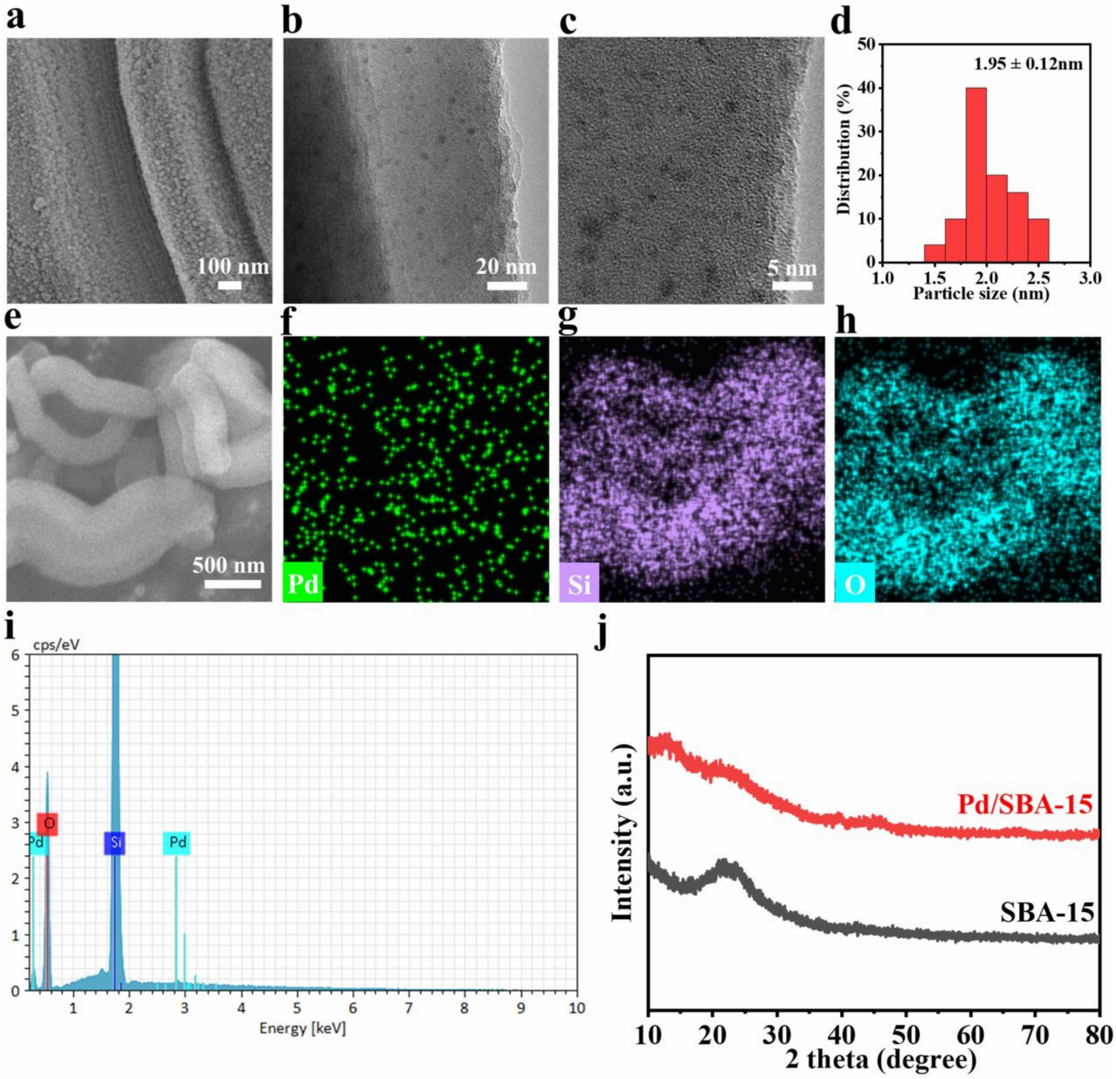

Figure S54. Characterization of Pd/SBA-15. (a) SEM and (b, c) TEM images of Pd/SBA-15. (d) The corresponding size distribution of Pd NPs of Pd/SBA-15. (e-h) SEM plus EDS mapping images and (i) the corresponding elemental spectrum of Pd/SBA-15. (j) PXRD patterns of SBA-15 and Pd/SBA-15.

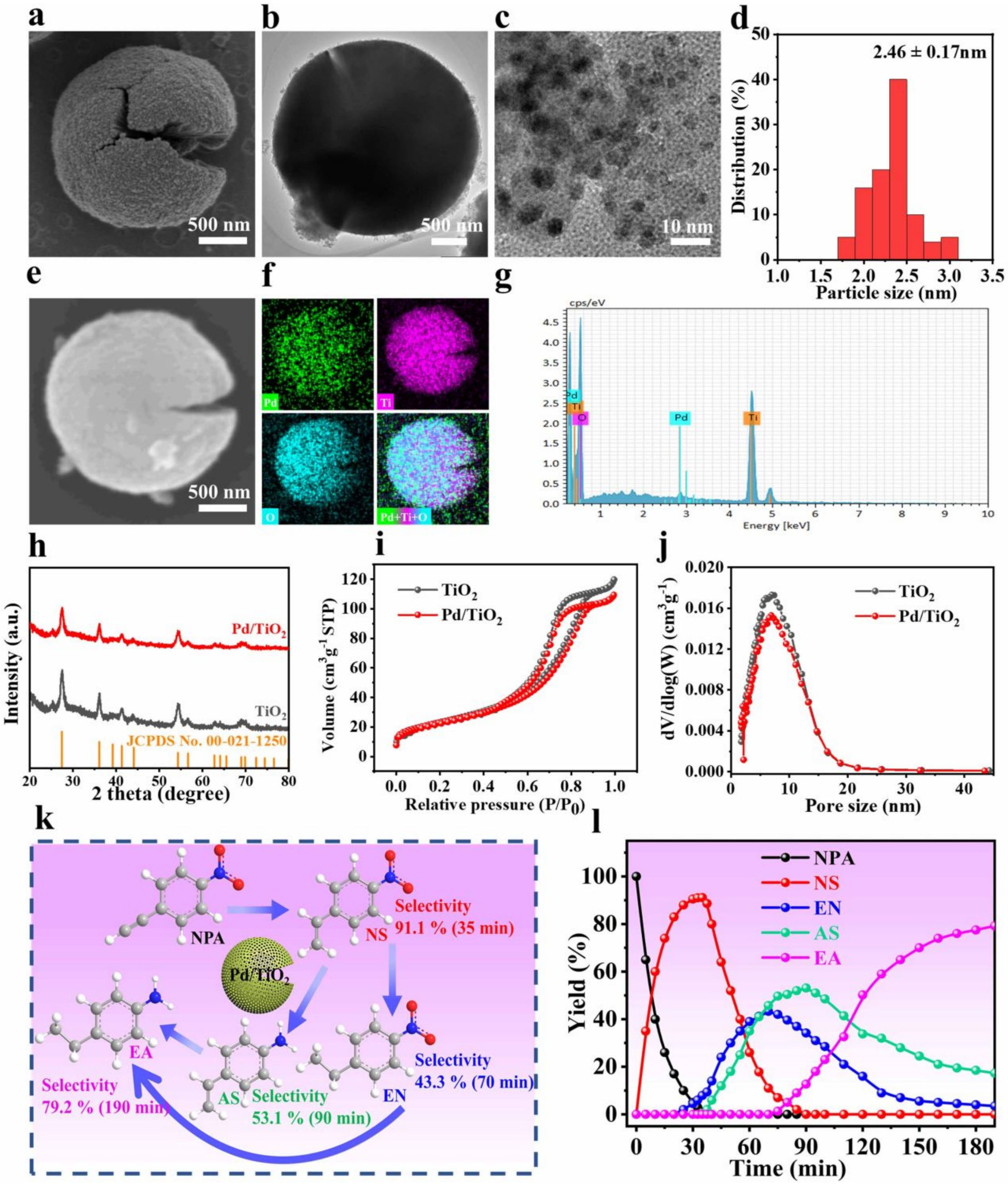

Figure S55. Characterization of Pd/TiO$_2$. (a) SEM and (b, c) TEM images of **Pd/TiO$_2$**. (d) The corresponding size distribution of Pd NPs of Pd/TiO$_2$. (e, f) SEM plus EDS mapping images and (g) the corresponding elemental spectrum of Pd/TiO$_2$. (h) PXRD patterns, (i) N_2 adsorption/desorption isotherms and (j) pore size distributions of TiO$_2$ and Pd/TiO$_2$ (based on DFT model by using the desorption branches of their isotherms).

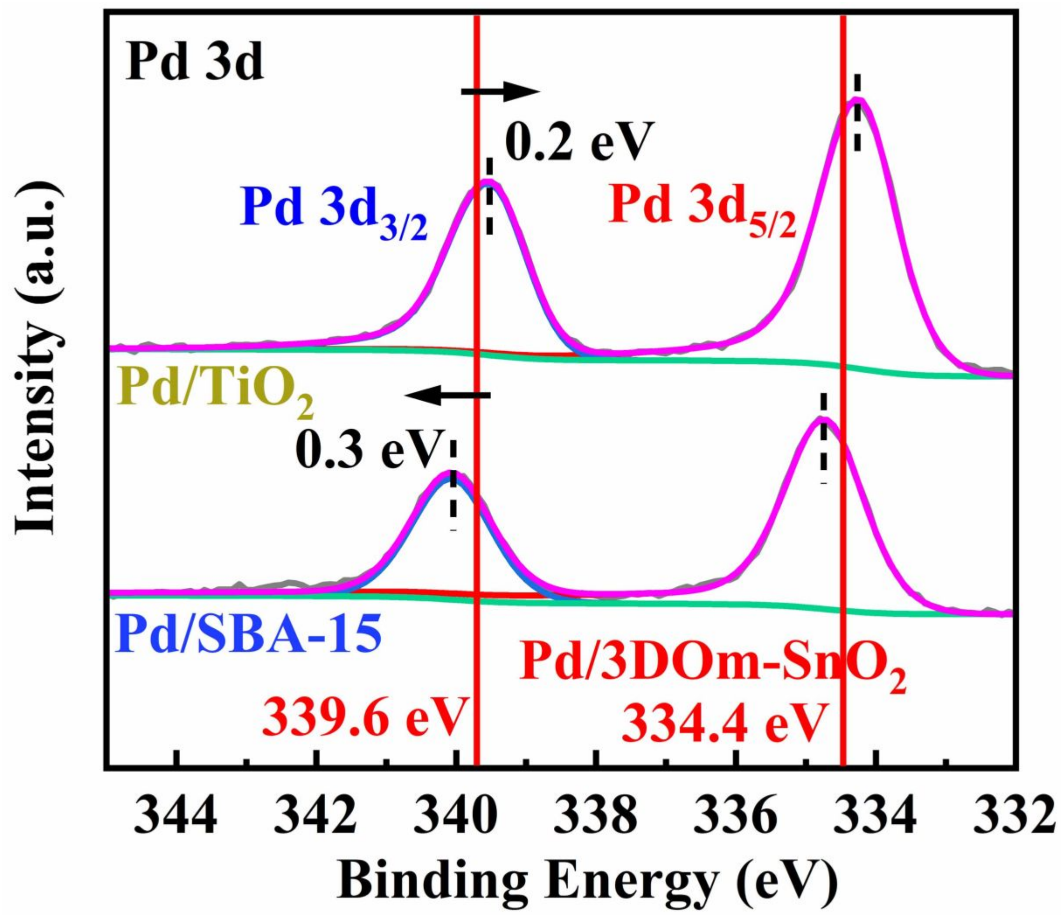

Figure S56. Pd 3d XPS spectra of Pd/TiO_2 and Pd/SBA-15, which indicate that the binding energies of Pd/SBA-15 and Pd/TiO_2 show a positive shift of ~0.3 eV and a negative shift of ~0.2 eV as compared with those of Pd/3DOm-SnO_2(27). These results demonstrate that excessive electrons are accumulated on the Pd NPs of Pd/TiO_2, while insufficient electrons are accumulated on the Pd NPs of Pd/SBA-15.

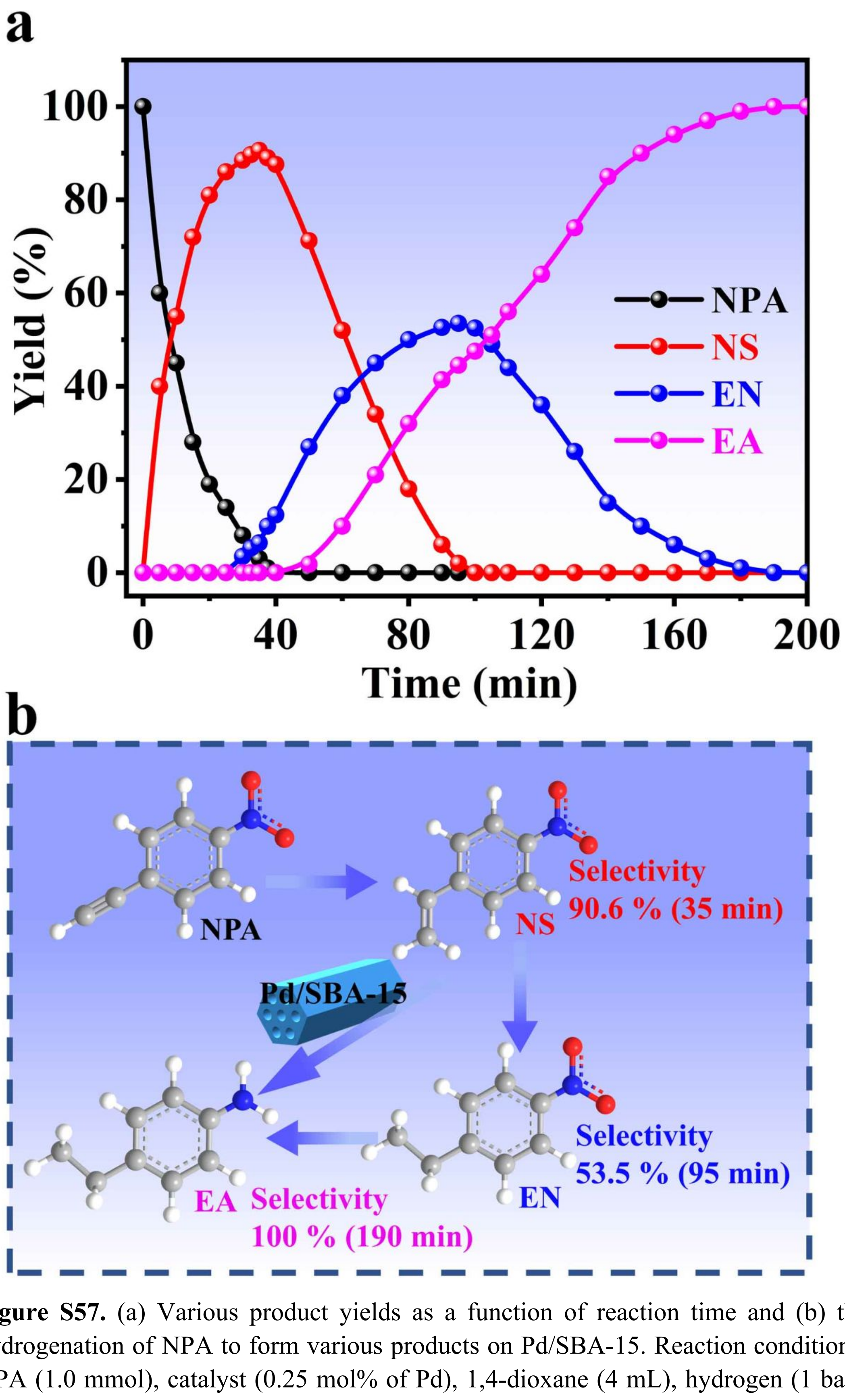

Figure S57. (a) Various product yields as a function of reaction time and (b) the hydrogenation of NPA to form various products on Pd/SBA-15. Reaction conditions: NPA (1.0 mmol), catalyst (0.25 mol% of Pd), 1,4-dioxane (4 mL), hydrogen (1 bar), room temperature, 200 min.

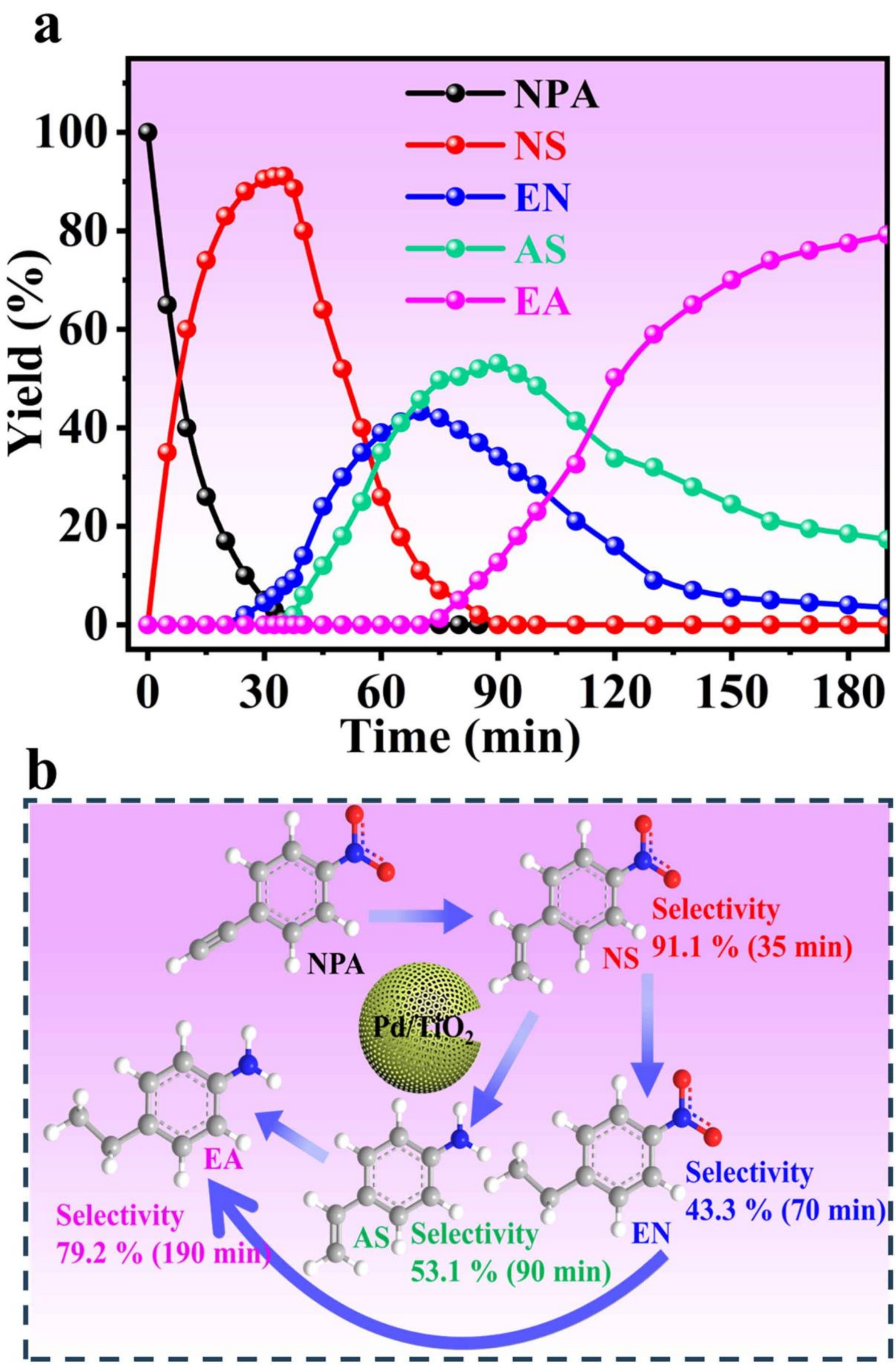

Figure S58. (a) Various product yields as a function of reaction time and (b) sequential hydrogenation of NPA to form various products on Pd/TiO_2.

Reaction conditions: NPA (1.0 mmol), catalyst (0.25 mol% of Pd), 1,4-dioxane (4 mL), hydrogen (1 bar), room temperature, 190 min.

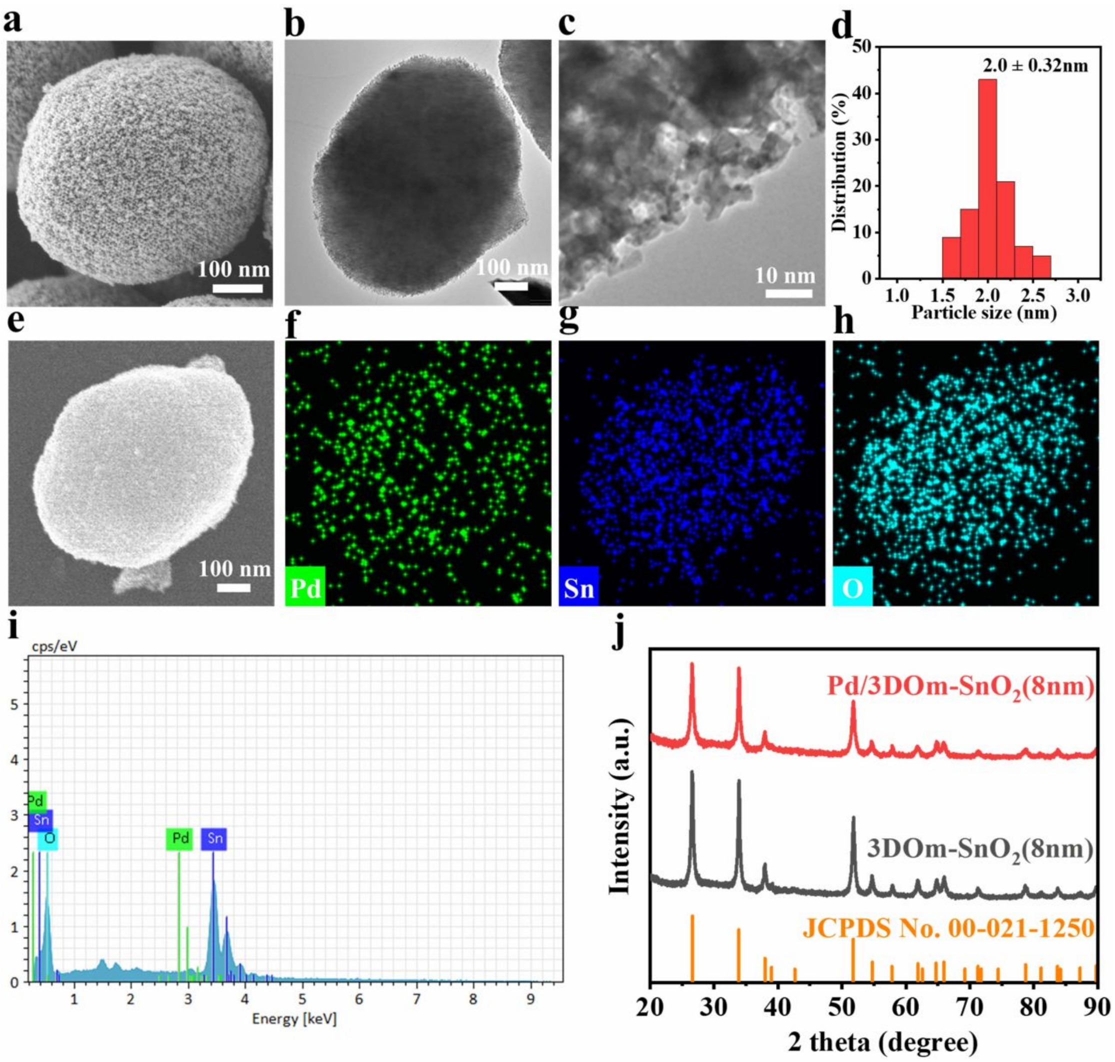

Figure S59. Characterization of Pd/3DOm-SnO$_2$(8). (a) SEM and (b, c) TEM images of Pd/3DOm-SnO$_2$(8). (d) The corresponding size distribution of Pd NPs of Pd/3DOm-SnO$_2$(8). (e-h) SEM plus EDS mapping images and (i) the corresponding elemental spectrum of Pd/3DOm-SnO$_2$(8). (j) PXRD patterns of 3DOm-SnO$_2$(8) and Pd/3DOm-SnO$_2$(8).

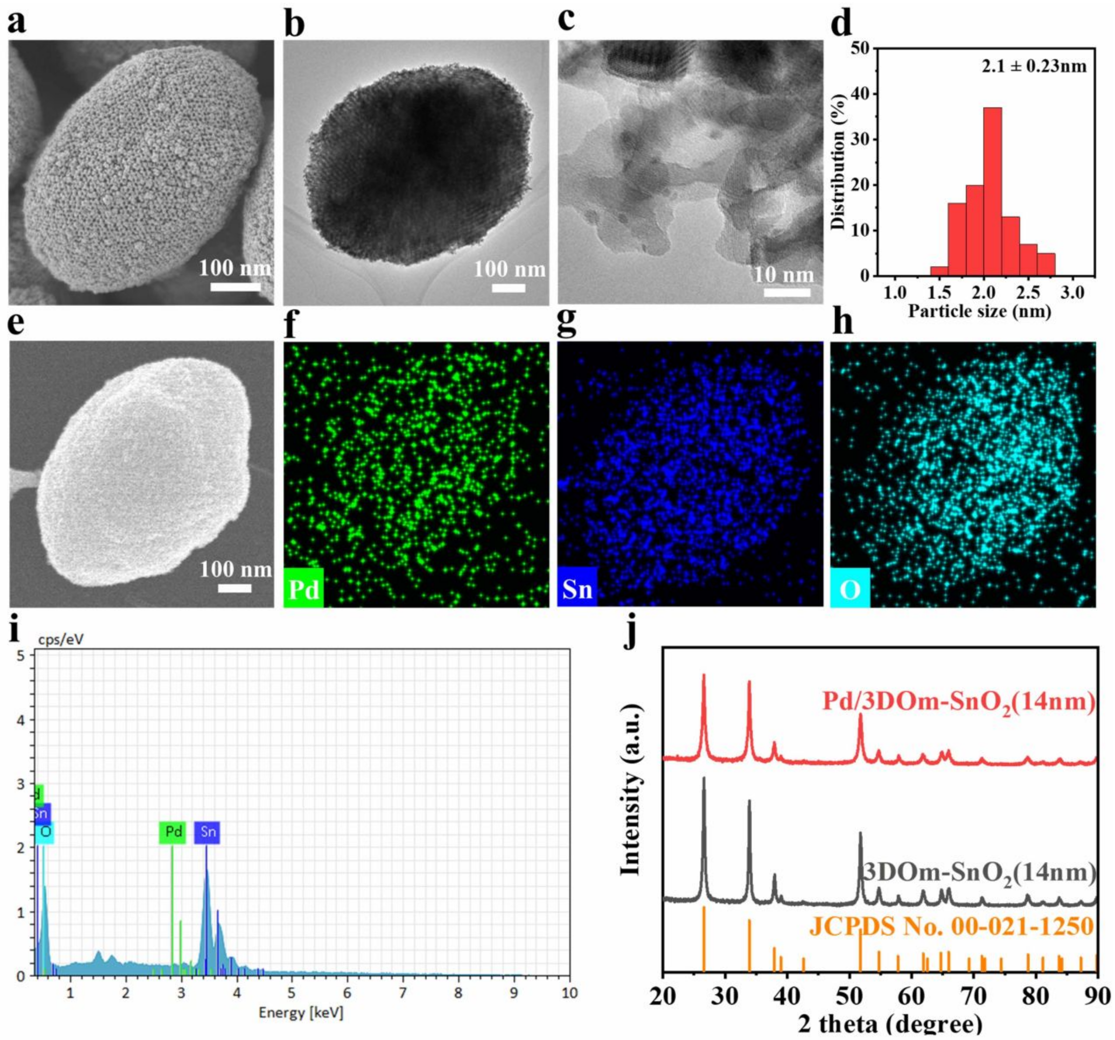

Figure S60. Characterization of Pd/3DOm-SnO_2(14). (a) SEM and (b, c) TEM images of Pd/3DOm-SnO_2(14). (d) The corresponding size distribution of Pd NPs of Pd/3DOm-SnO_2(14). (e-h) SEM plus EDS mapping images and (i) the corresponding elemental spectrum of Pd/3DOm-SnO_2(14). (j) PXRD patterns of 3DOm-SnO_2(14) and Pd/3DOm-SnO_2(14).

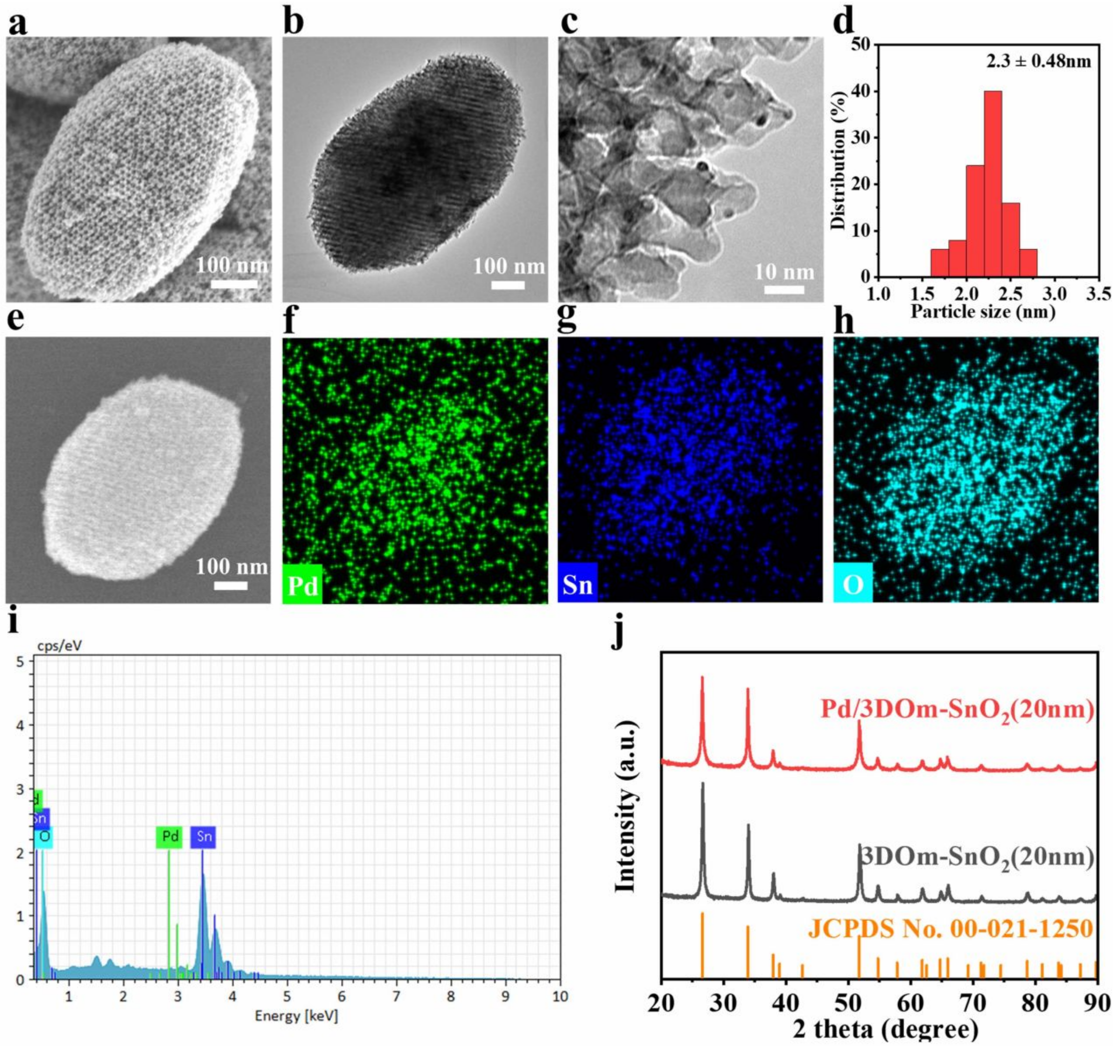

Figure S61. Characterization of Pd/3DOm-SnO_2(20). (a) SEM and (b, c) TEM images of Pd/3DOm-SnO_2(20). (d) The corresponding size distribution of Pd NPs in Pd/3DOm-SnO_2(20). (e-h) SEM plus EDS mapping images and (i) the corresponding elemental spectrum of Pd/3DOm-SnO_2(20). (j) PXRD patterns of 3DOm-SnO_2(20) and Pd/3DOm-SnO_2(20).

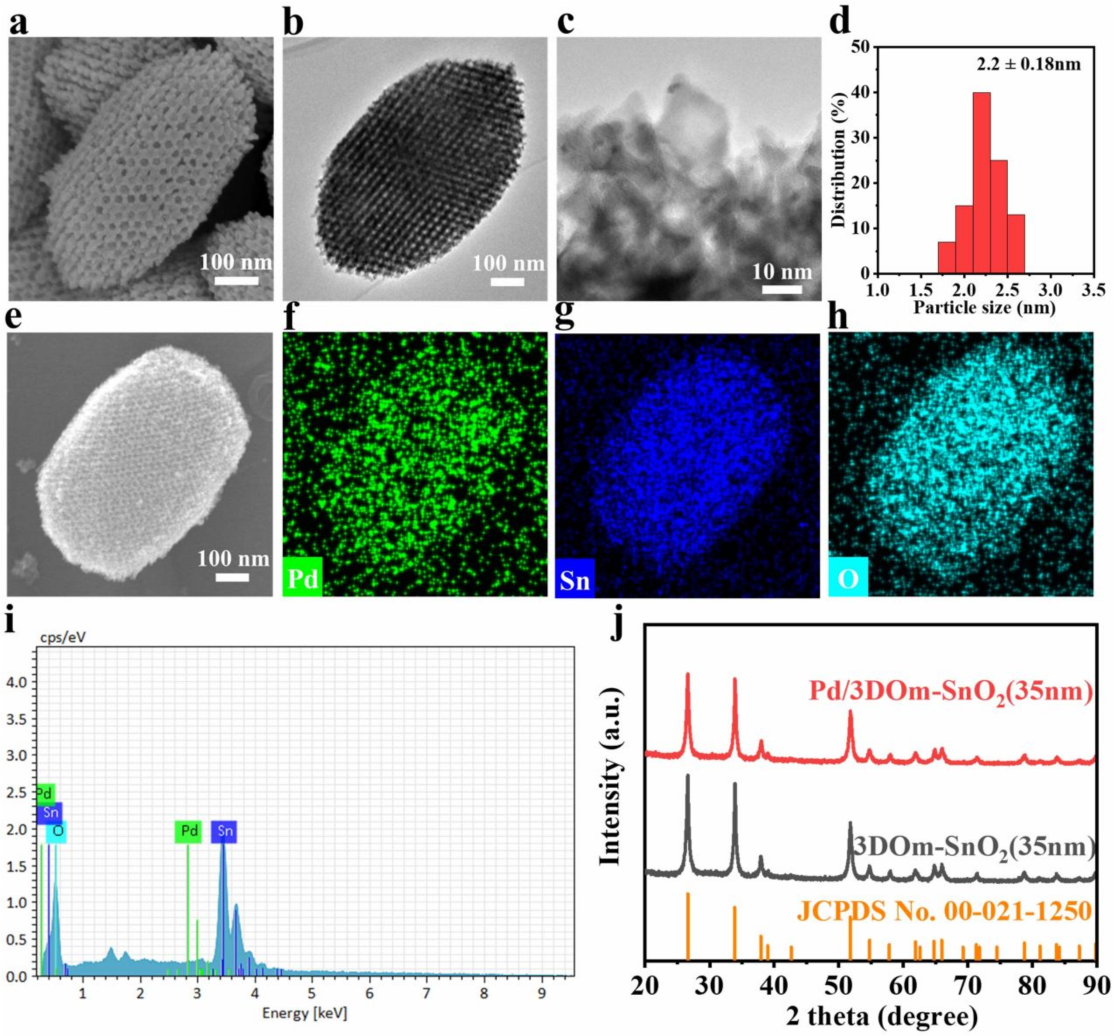

Figure S62. Characterization of Pd/3DOm-SnO$_2$(35). (a) SEM and (b, c) TEM images of Pd/3DOm-SnO$_2$(35). (d) The corresponding size distribution of Pd NPs of Pd/3DOm-SnO$_2$(35). (e-h) SEM plus EDS mapping images and (i) the corresponding elemental spectrum of Pd/3DOm-SnO$_2$(35). (j) PXRD patterns of 3DOm-SnO$_2$(35) and Pd/3DOm-SnO$_2$(35).

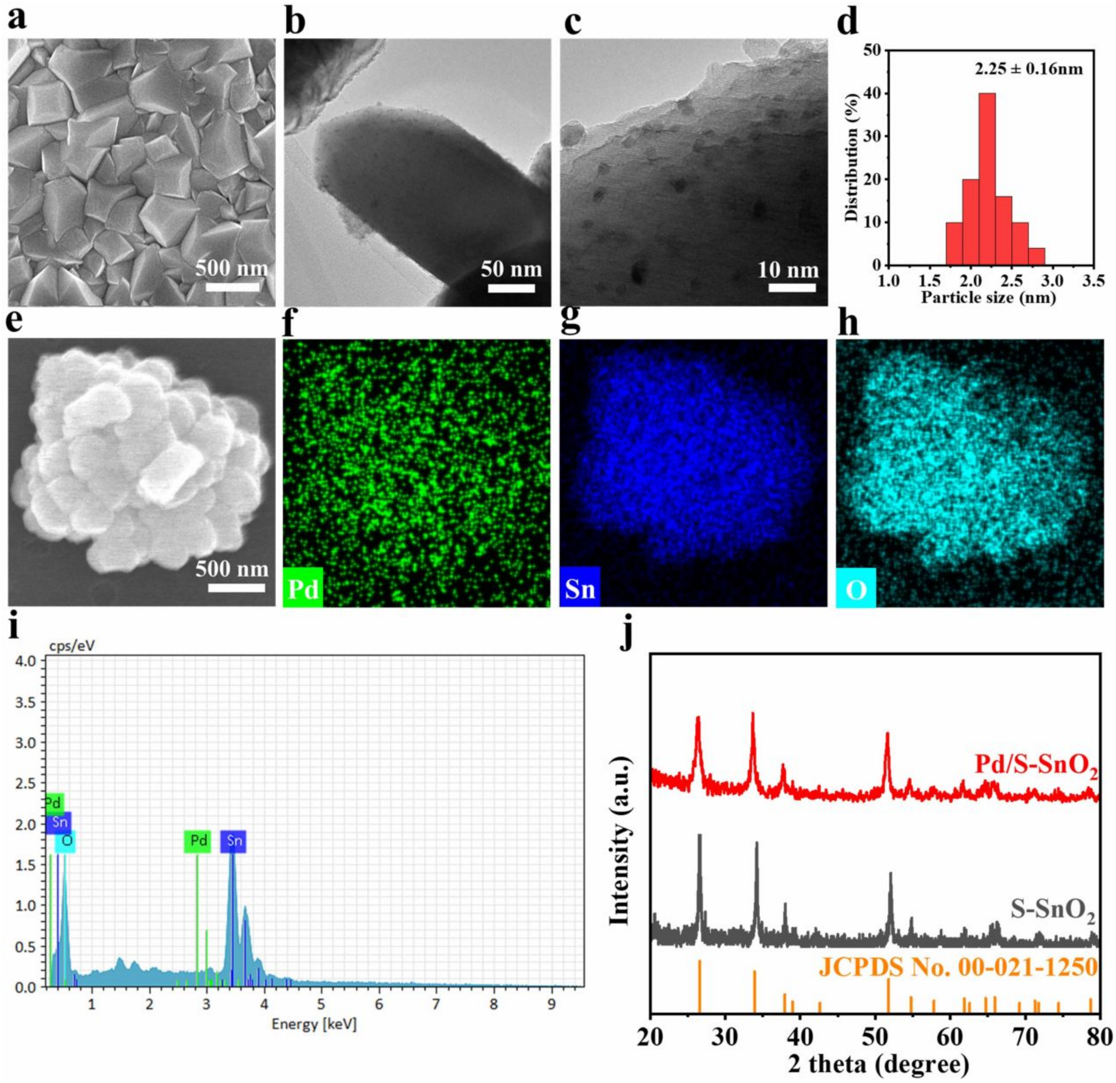

Figure S63. Characterization of Pd/S-SnO$_2$. (a) SEM and (b, c) TEM images of Pd/S-SnO$_2$. (d) The corresponding size distribution of Pd NPs of Pd/S-SnO$_2$. (e-h) SEM plus EDS mapping images and (i) the corresponding elemental spectrum of Pd/S-SnO$_2$. (j) PXRD patterns of S-SnO$_2$ and Pd/S-SnO$_2$.

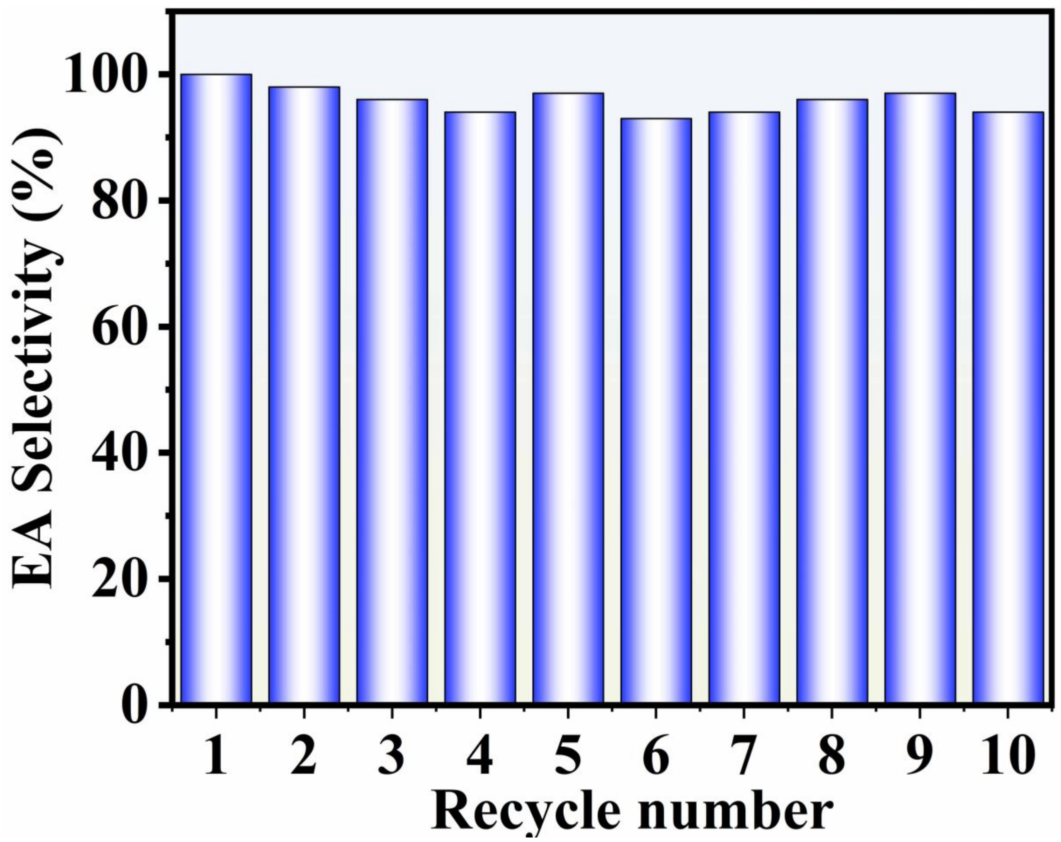

Figure S64. Recyclability experiments of Pd/3DOm-SnO_2 for the hydrogenation of NPA to EA. Reaction conditions: catalyst (0.25 mol% of Pd), 1,4-dioxane (8 mL), hydrogen (1 bar), room temperature, 170 min.

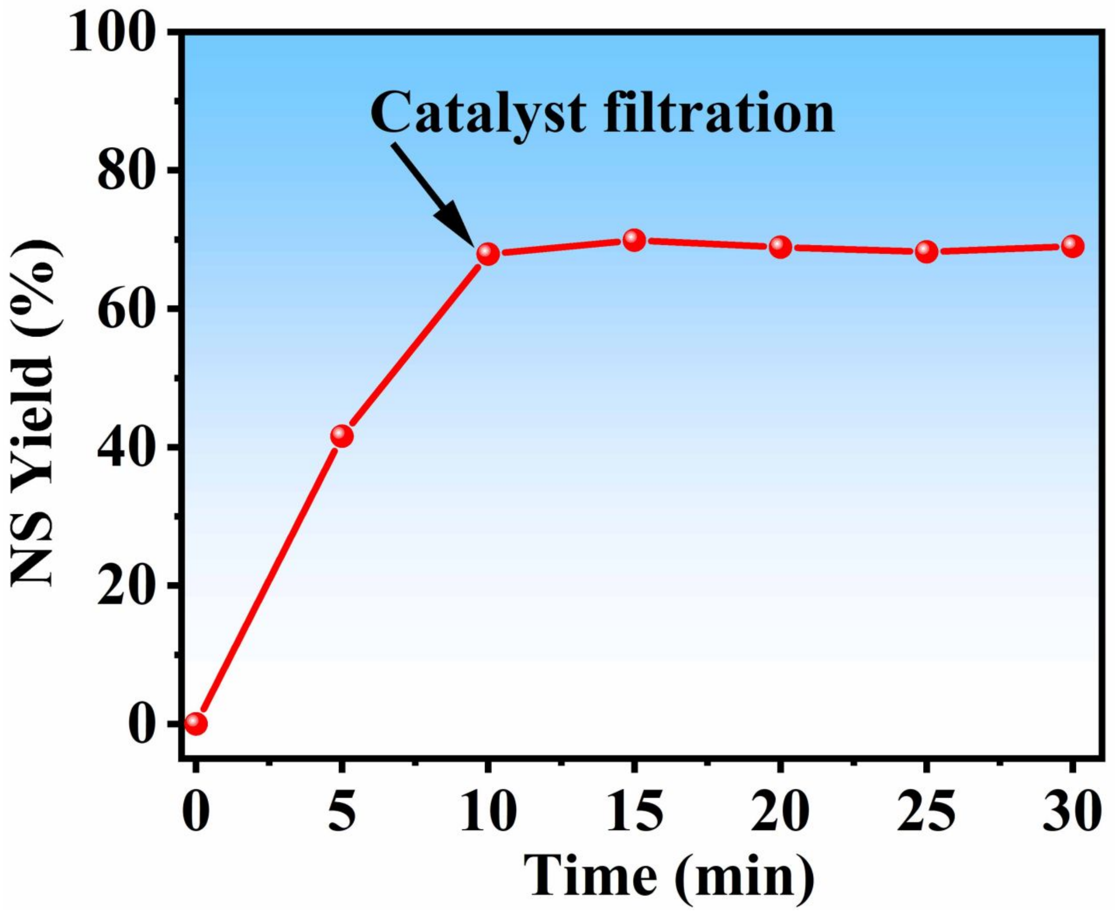

Figure S65. Hot filtration test of Pd/3DOm-SnO_2(27). Reaction conditions: NPA (1.0 mmol), catalyst (0.25 mol% of Pd), 1,4-dioxane (4 mL), hydrogen (1 bar), room temperature, 30 min. Conversion and yield were measured by GC-MS. Clearly, no more increment in the conversion of NPA is observed after a filtration process, suggesting the heterogeneous nature of the catalytic system.

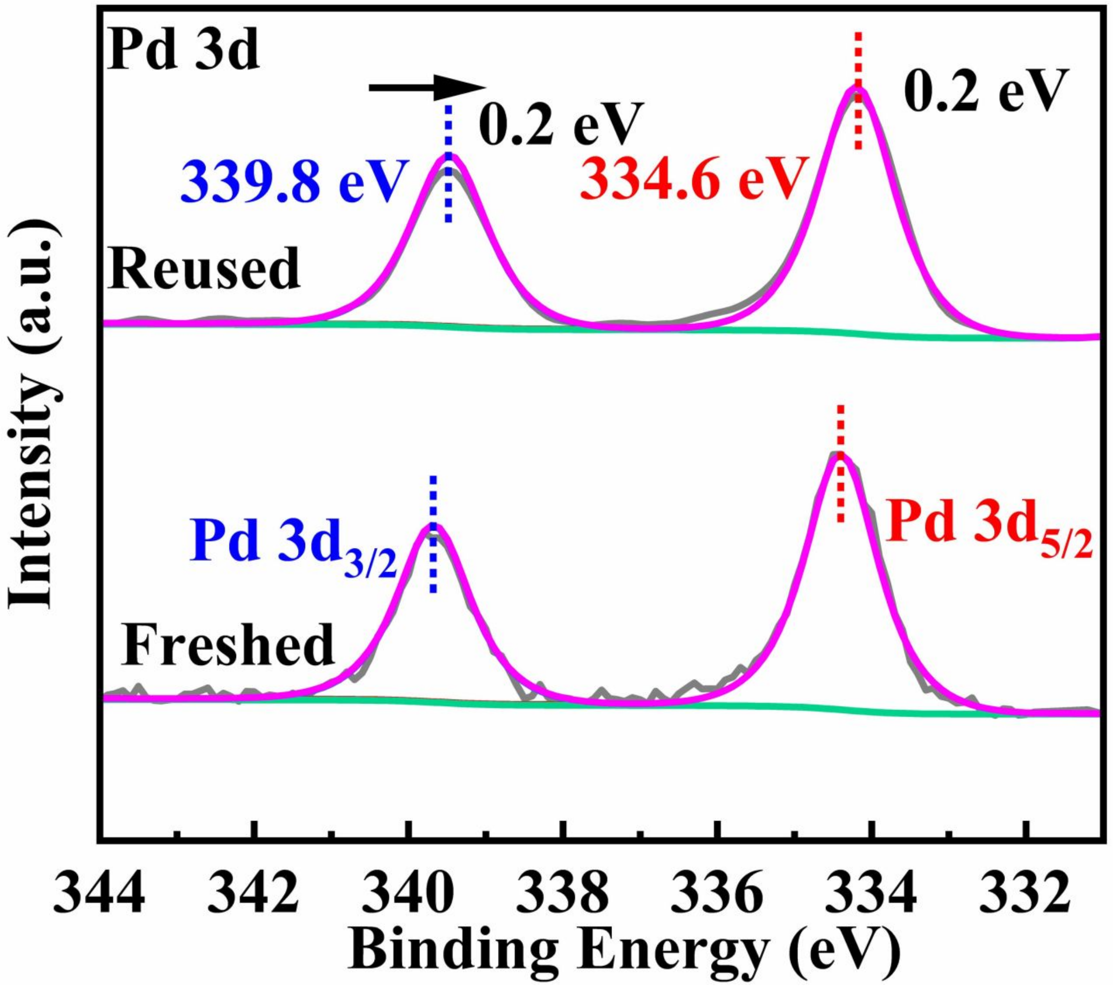

Figure S66. Pd 3d XPS spectra of the fresh and used Pd/3DOm-SnO_2(27). The used Pd/3DOm-SnO_2 exhibits two prominent bands at 339.8 eV for Pd $3d_{3/2}$ and 334.6 eV for Pd $3d_{5/2}$ in its Pd 3d spectrum. Both the Pd 3d bonding energies are lower by ~0.2 eV than those of the fresh one, which may be caused by the slight aggregation of Pd NPs after recyclings as revealed by its TEM images (Figure S67).

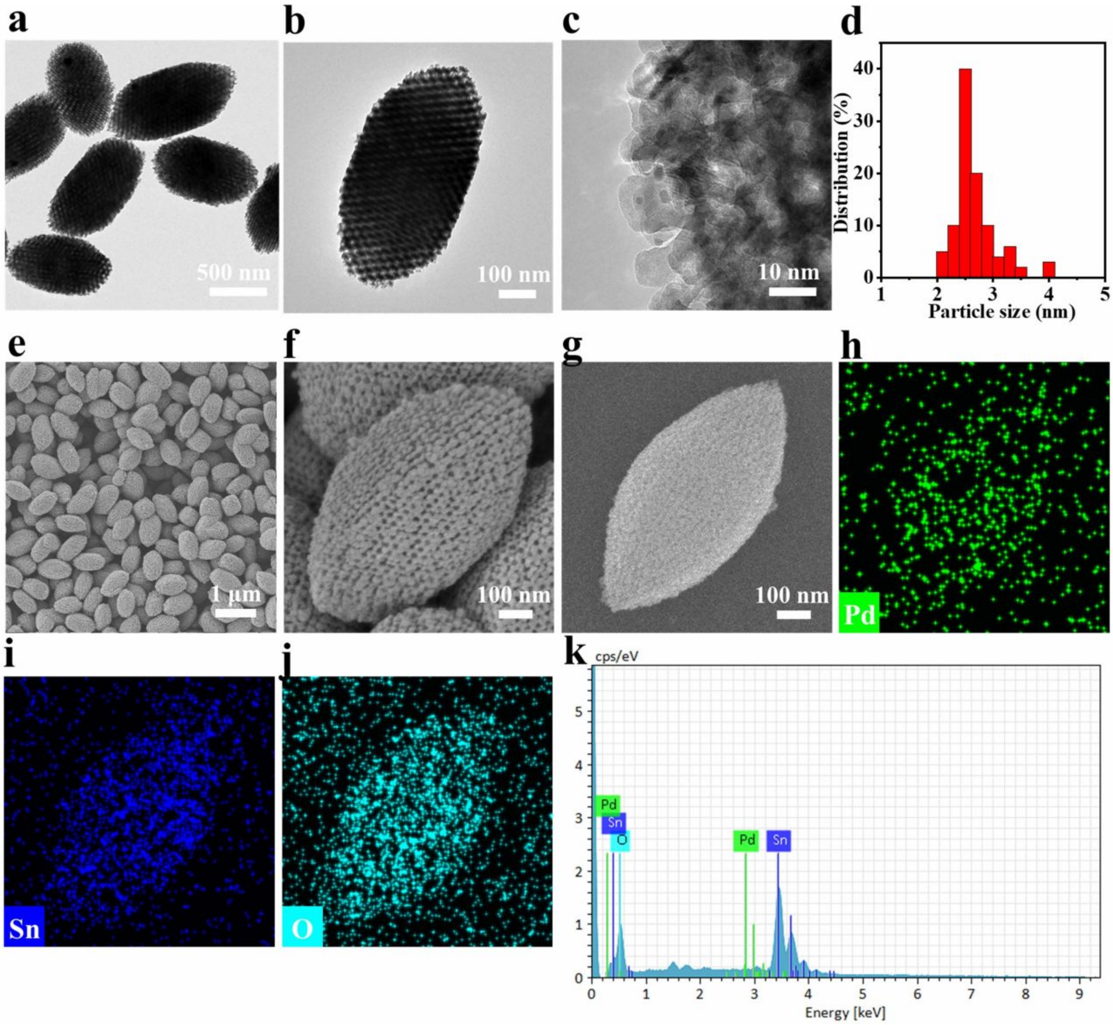

Figure S67. Characterization of the used Pd/3DOm-SnO_2(27) after the hydrogenation reaction. (a-c) TEM images of Pd/3DOm-SnO_2(35). (d) The corresponding size distribution of Pd NPs of Pd/3DOm-SnO_2(35). (e-j) SEM plus EDS mapping images and (k) the corresponding elemental spectrum of Pd/3DOm-SnO_2(35).

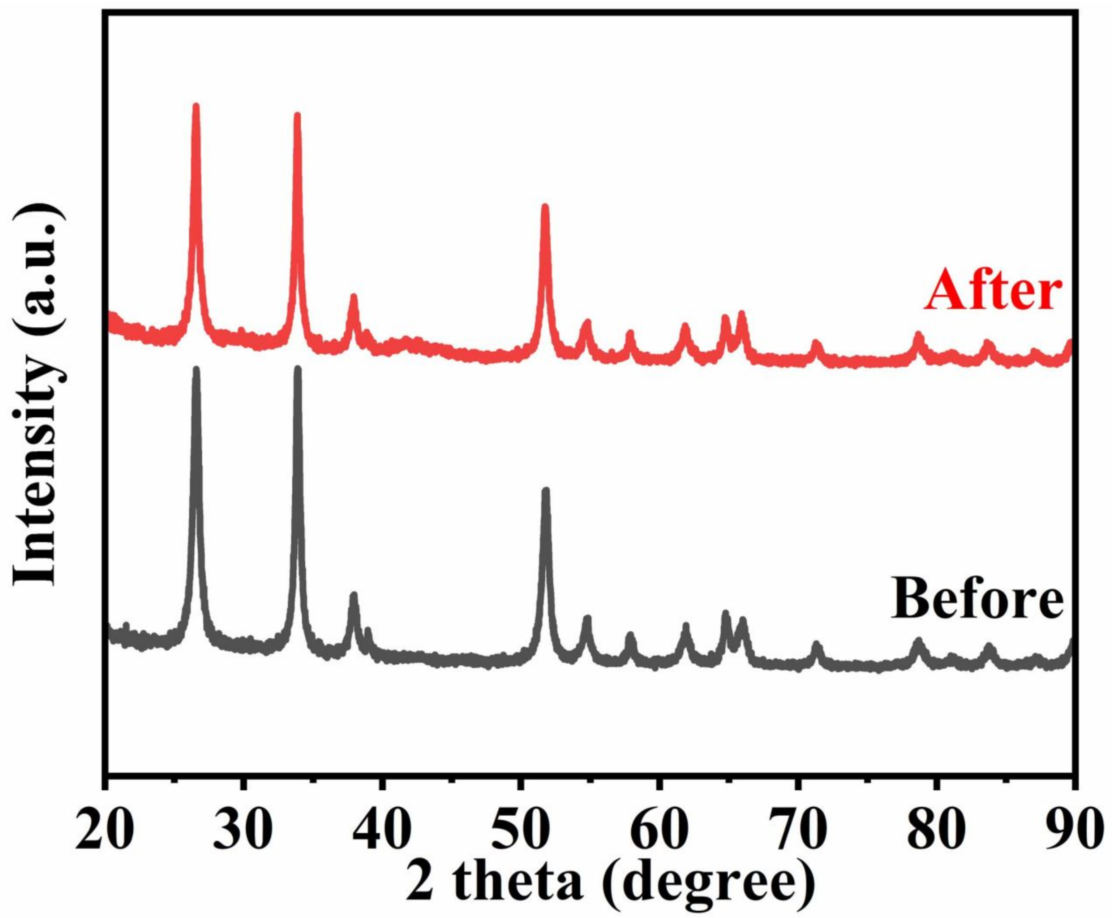

Figure S68. XRD patterns of the Pd/3DOm-SnO_2(27) before and after the hydrogenation reaction.

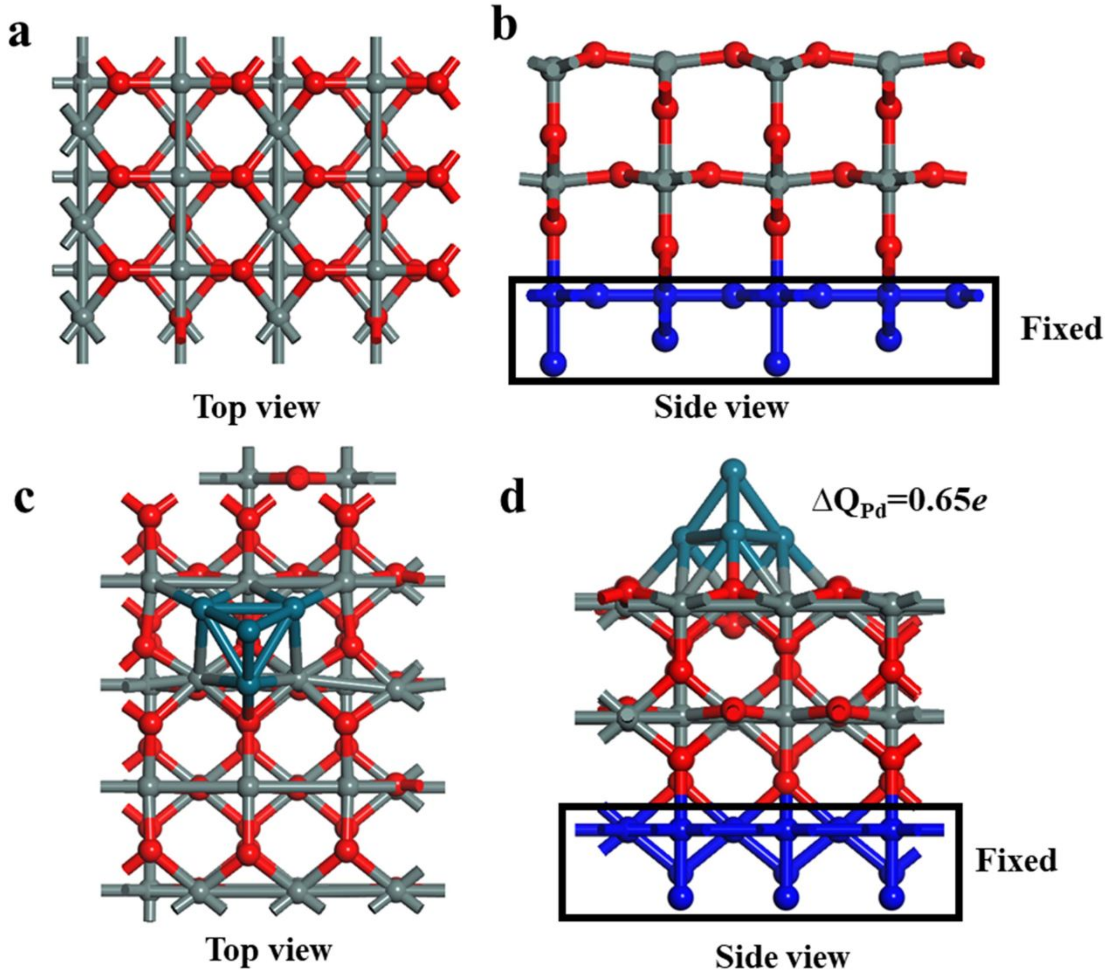

Figure S69. (a, b) Optimized structures of SnO_2(110) surface. (c, d) Optimized structures of Pd_4/SnO_2 surface and Bader charge. Cyan, gray and red balls represent Pd, Sn and O atoms, respectively.

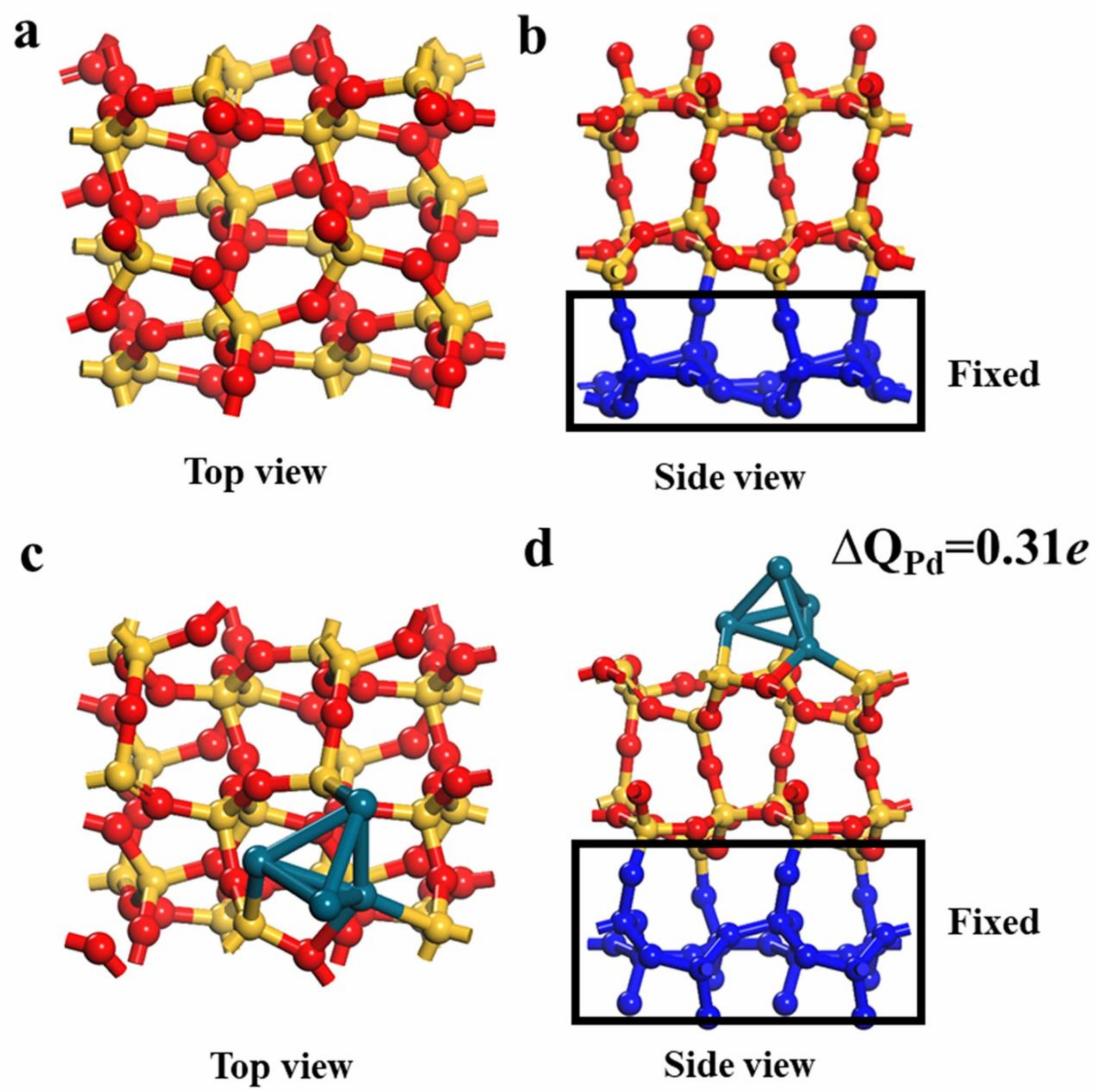

Figure S70. (a, b) Optimized structures of SiO_2(101) surface. (c, d) Optimized structures of Pd_4/SiO_2 surface and Bader charge. Cyan, yellow and red balls represent Pd, Si and O atoms, respectively.

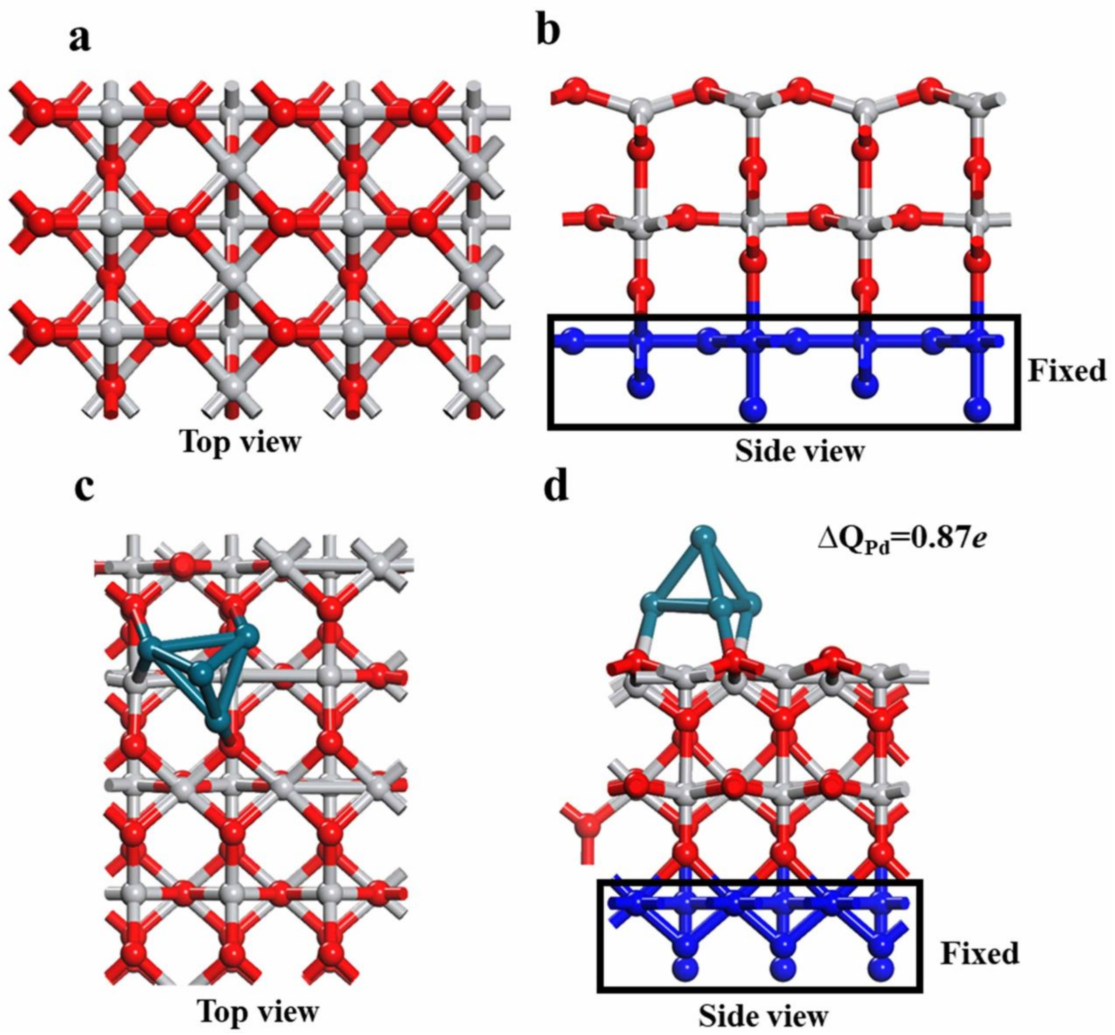

Figure S71. (a, b) Optimized structures of TiO_2(101) surface. (c, d) Optimized structures of Pd_4/TiO_2 surface and Bader charge. Cyan, light gray and red balls represent Pd, Ti and O atoms, respectively.

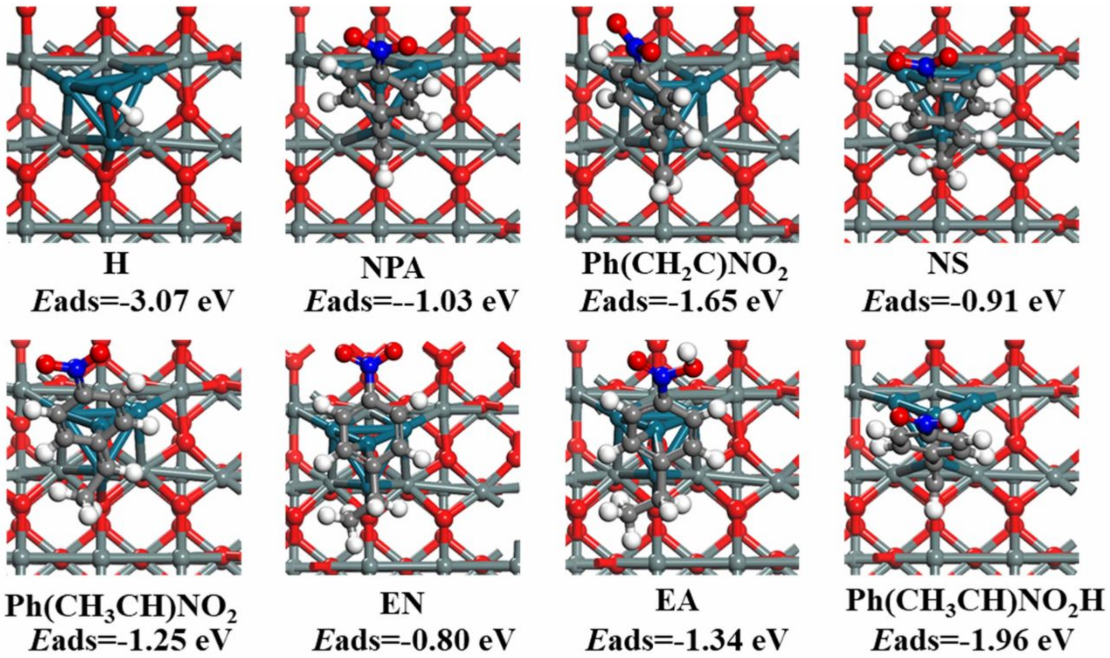

Figure S72. Stable adsorption configurations and adsorption free energies (eV) for various reaction species adsorbed on Pd_4/SnO_2.

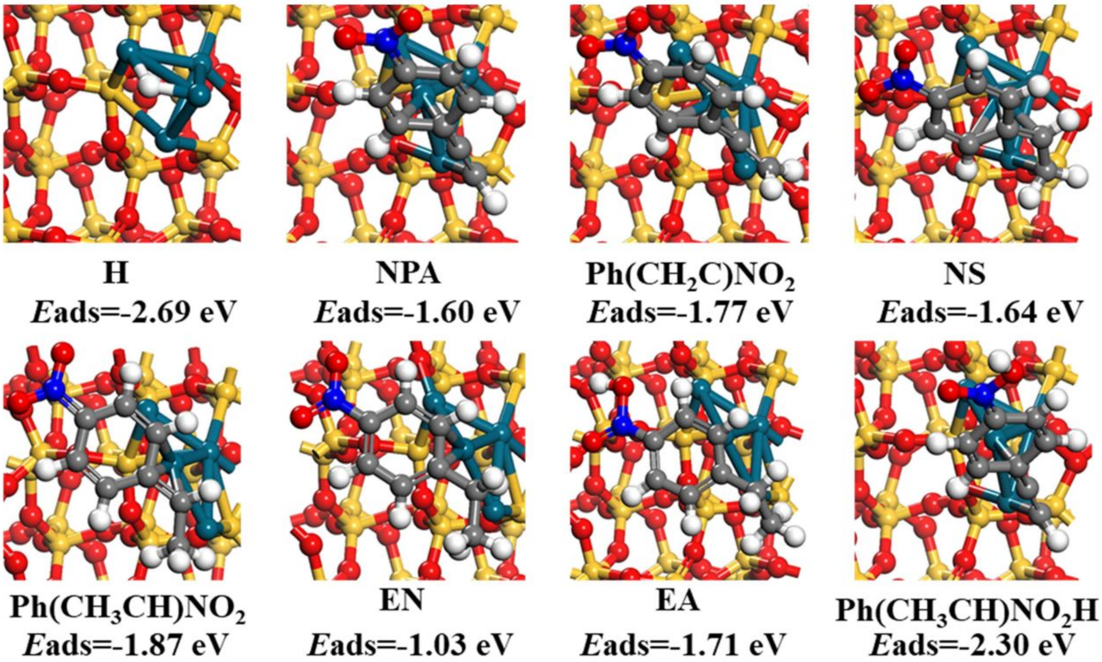

Figure S73. Stable adsorption configurations and adsorption free energies (eV) for various reaction species adsorbed on Pd_4/SiO_2.

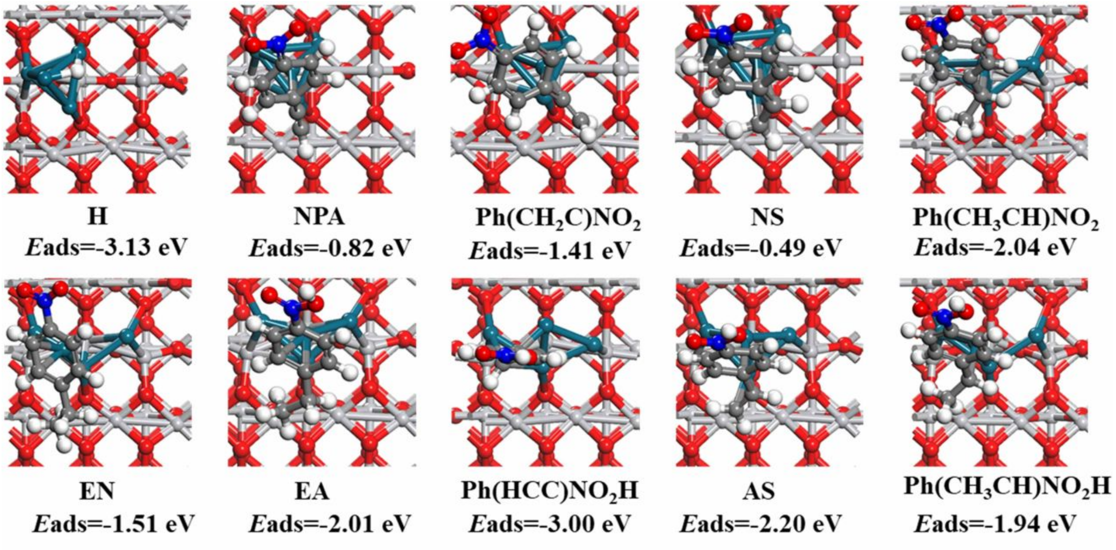

Figure S74. Stable adsorption configurations and adsorption free energies (eV) for various reaction species adsorbed on Pd_4/TiO_2.

Supporting Tables

Table S1. Yields of 3DOm-SnO_2 and S-SnO_2 in three repeated experiments.

Number	Theoretical quantities[a] (mg)	Actual quantities of 3DOm-SnO_2 (mg)	Actual quantities of S-SnO_2 (mg)	Yields of 3DOm-SnO_2[b] (%)	Yields of S-SnO_2[b] (%)
1	286.1	275.4	3.2	96.3	1.1
2	286.1	278.6	2.6	97.4	0.9
3	286.1	269.1	6.1	94.1	2.1

[a] The theoretical quantities of various products are calculated by considering the purity of tin chloride pentahydrate to be 99%

[b] Yields of 3DOm-SnO_2 and S-SnO_2 are calculated by the actual quantities divided by the theoretical quantities.

The experimental conditions; 0.673 g of $SnCl_4·5H_2O$, 10 ml of H_2O + 10 mL of C_2H_5OH, with or without 2 g of 3DO-SiO_2, 200 ℃, 12 h.

Table S2. BET surface areas, pore sizes and total pore volumes of various samples.

**Catalysts**	S_{BET} (m^2/g)	Pore size[a] (nm)	Total pore volume (cm^3/g)
S-SnO_2	2.5	-	0.0074
3DOm-SnO_2(8)	101	8.0	0.74
3DOm-SnO_2(14)	87	14.0	0.61
3DOm-SnO_2(20)	78	20.0	0.56
3DOm-SnO_2(27)	64	27.0	0.42
3DOm-SnO_2(35)	57	35.0	0.34
Pd/3DOm-SnO_2(27)	62	26.0	0.40

[a] Based on DFT model by using the desorption branches of the isotherms of various samples.

Table S3. Assignment of the O_{ads} and O_{latt} components calculated from the XPS results.

**Catalysts**	O_{ads} ratio[a](%)	O_{latt} ratio[a](%)
S-SnO_2	47.51	52.49
3DOm-SnO_2	64.57	35.43
Pd/3DOm-SnO_2	60.32	39.68

[a] The amounts of O_{ads} and O_{latt} were calculated from the peak areas of the O 1s components using the following equations:

O_{ads} ratio (%) = $(O_{ads} / (O_{ads} + O_{latt}))\times 100\%$

O_{latt} ratio (%) = $(O_{latt} / (O_{ads} + O_{latt}))\times 100\%$

Table S4. Crystallinity of various 3DOm-SnO_2-T samples by assuming that the crystallinity of 3DOm-SnO_2-450 is 100%.

**Catalysts**	Integrated areas of the XRD peaks	Crystallinity[a] (%)
3DOm-SnO_2-450	1135.3	100
3DOm-SnO_2-550	958.3	84.4
3DOm-SnO_2-700	914.2	80.5
3DOm-SnO_2-800	894.5	78.8
3DOm-SnO_2-900	725.4	63.9
3DOm-SnO_2-1000	451.0	39.8

Table S5. The loading amounts of Pd in various catalysts.

**Catalysts**	Theoretical loading (wt%)	[a]Actual loading (wt%)
Pd/3DOm-SnO_2(35)	1	0.98
Pd/3DOm-SnO_2(27)	1	0.96
Pd/3DOm-SnO_2(20)	1	0.94
Pd/3DOm-SnO_2(14)	1	0.93
Pd/3DOm-SnO_2(8)	1	0.97
Pd/S-SnO_2	1	0.93
Pd/SBA-15	1	0.94
Pd/TiO_2	1	0.95

[a] Measured by atomic absorption spectroscopy (AAS).

Table S6. Sequential hydrogenation of NPA over various catalysts.

**Catalysts**	Time (min)	Conversion (%)	Selectivity (%)				
			NS	EN	APA	AS	EA
Pd/3DOm-SnO_2(27)	33	99.2	98.7	0.5	0	0	0
	85	100	0	98.1	0	0	1.9
	170	100	0	0	0	0	100
Pd/SBA-15	35	97	90.6	6.4	0	0	0
	95	100	2	53.5	0	0	44.5
	190	100	0	0	0	0	100
Pd/TiO_2	35	99	91.1	7.9	0	0	0
	70	100	11	43.3	0	45.7	0
	90	100	0	34.2	0	53.1	12.7
	190	100	0	3.5	0	17.3	79.2

Reaction conditions: NPA (1.0 mmol), catalyst (0.25 mol% of Pd), 1,4-dioxane (4 mL), hydrogen (1 bar), 30 °C.

Table S7. Kinetic data for the hydrogenation of NPA to NS over various catalyst.

Time (min)	-ln(1-X)					
	Pd/S-SnO_2	Pd/3DOm-SnO_2(35)	Pd/3DOm-SnO_2(27)	Pd/3DOm-SnO_2(20)	Pd/3DOm-SnO_2(14)	Pd/3DOm-SnO_2(8)
5	0.098	0.386	0.521	0	0	0
10	0.203	0.783	1.182	0.654	0.781	0.853
15	0.326	1.294	1.85	1.55	1.88	2.1
20	0.442	1.86	2.66	2.5	2.74	3.18
25	0.6	2.41	3.45	-	-	-
30	0.719	2.96	-	-	-	-
40	1.01	-	-	-	-	-
50	1.252	-	-	-	-	-

Reaction conditions: NPA (1.0 mmol), catalyst (0.25 mol% of Pd), 1,4-dioxane (4 mL), hydrogen (1 bar), 30 °C.

-ln (1-X) = kt; X is the conversion of NPA, we plotted figures with -ln (1-X) as the y-axis and time as the x-axis, fitting the data linearly and evaluated reaction rate constants from the slopes, and the results are showed in Figure 5f.

Table S8. Hydrogenation performances of Pd/DOm-SnO_2(27) and benchmark catalysts reported in the literatures previously.

**Catalysts**	substrate	product	Con. (%)	Sel. (%)	Ref.
h-mesoPdS	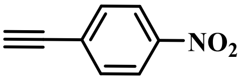	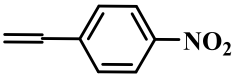	100	94	Ref.[10]
Meso-i-Pt_1Sn_1	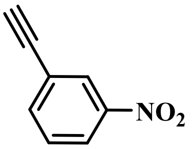	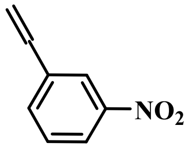	100	95.1	Ref.[11]
Pd@Ag@CeO_2	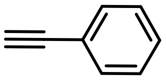	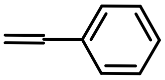	97	99	Ref.[12]
Au@L_a-ZIF-90	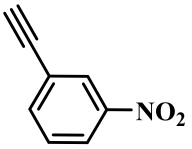	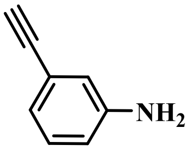	100	99	Ref.[13]
Hetero-phase Pd NSs	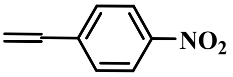	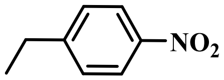	100	99	Ref.[14]
Pt-B@IL	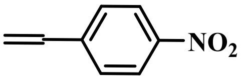	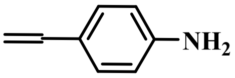	100	86	Ref.[15]
0.2%Pt/TiO_2	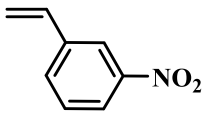	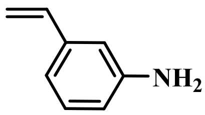	99	90	Ref.[16]
Pt/FeOx-R250	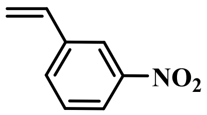	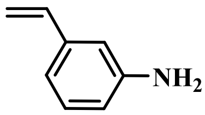	96.5	98.6	Ref.[17]
3D Au3Pd1 NDs	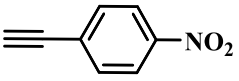	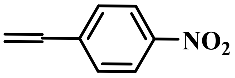	100	99.5	Ref.[18]
		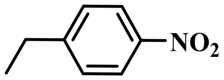	100	96.7	
Pd/3DOm-SnO_2(27)	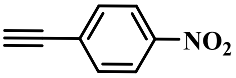	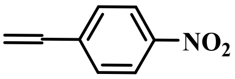	100	98.7	This work
		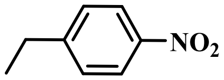	100	98.1	
		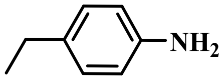	100	100	

Table S9. Required activation energy and reaction heat for each step of the sequential hydrogenation of NPA on Pd_4/SnO_2.

Reaction	E_a(eV)	ΔE(eV)
**NPA+H→Ph(CCH$_2$)NO$_2$**	1.24	-0.40
**Ph(CCH$_2$)NO$_2$+H→NS**	0.86	-0.75
**NS+H→Ph(CH$_3$CH)NO$_2$**	0.99	-0.16
**Ph(CH$_3$CH)NO$_2$+H→EN**	0.68	-0.47
**EN+H→Ph(CH$_3$CH$_2$)NO$_2$H**	1.90	0.35
**NPA+H→Ph(HCC)NO$_2$H**	2.46	-0.52

(a1) = Ph(HCC)NO$_2$H, (a2) = Ph(CCH$_2$)NO$_2$, (a3) = NS, (a4) = Ph(CH$_3$CH)NO$_2$, (a5) = EN, (a6) = Ph(CH$_3$CH$_2$)NO$_2$H, "TS" denotes a transition state, E_a denotes activation energy, ΔE denotes reaction heat.

Table S10. Required activation energy and reaction heat for each step of the sequential hydrogenation of NPA on Pd_4/SiO_2.

Reaction	E_a(eV)	ΔE(eV)
**NPA+H→Ph(CCH$_2$)NO$_2$**	1.19	0.20
**Ph(CCH$_2$)NO$_2$+H→NS**	0.49	-1.29
**NS+H→Ph(CH$_3$CH)NO$_2$**	0.61	0.024
**Ph(CH$_3$CH)NO$_2$+H→EN**	0.89	0.47
**EN+H→Ph(CH$_3$CH$_2$)NO$_2$H**	0.40	0.34
**NPA+H→Ph(HCC)NO$_2$H**	2.21	-0.14

(b1) = Ph(HCC)NO$_2$H, (b2) = Ph(CCH$_2$)NO$_2$, (b3) = NS, (b4) = Ph(CH$_3$CH)NO$_2$, (b5) = EN, (b6) = Ph(CH$_3$CH$_2$)NO$_2$H, "TS" denotes a transition state, E_a denotes activation energy, ΔE denotes reaction heat.

Table S11. Required activation energy and reaction heat for each step of the sequential hydrogenation of NPA on Pd_4/TiO_2.

**Reaction**	E_a**(eV)**	ΔE**(eV)**
**NPA+H→Ph(CCH$_2$)NO$_2$**	1.24	-0.074
**Ph(CCH$_2$)NO$_2$+H→NS**	0.77	0.27
**NS+H→Ph(CH$_3$CH)NO$_2$**	1.54	-0.213
**Ph(CH$_3$CH)NO$_2$+H→EN**	1.09	-0.14
**EN+H→Ph(CH$_3$CH$_2$)NO$_2$H**	1.60	0.345
**NS+H→Ph(CH$_2$CH)NO$_2$H**	1.78	-0.177
**Ph(CH$_2$CH)NO$_2$H+H→Ph(CH$_3$CH)NO$_2$H**	1.02	-0.13
**Ph(CH$_3$CH)NO$_2$H+H→Ph(CH$_3$CH$_2$)NO$_2$H**	1.08	0.135
**NPA+H→Ph(HCC)NO$_2$H**	2.18	-1.77

(c1) = Ph(HCC)NO$_2$H, (c2) = Ph(CCH$_2$)NO$_2$, (c3) = NS, (c4) = Ph(CH$_3$CH)NO$_2$, (c5) = Ph(CH$_2$CH)NO$_2$H, (c6) = EN, (c7) = Ph(CH$_3$CH)NO$_2$H, (c8) = Ph(CH$_3$CH$_2$)NO$_2$H, (c9) = Ph(CH$_3$CH$_2$)NO$_2$H, "TS" denotes a transition state, E_a denotes activation energy, ΔE denotes reaction heat.

Supporting References

(1) Fan, W.; Snyder, M. A.; Kumar, S.; Lee, P. S.; Yoo, W. C.; McCormick, A. V.; Lee Penn, R.; Stein, A.; Tsapatsis, M. Hierarchical nanofabrication of microporous crystals with ordered mesoporosity. *Nat. Mater.* **2008**, *7*, 984-991.

(2) Lan, K.; Wang, R.; Zhang, W.; Zhao, Z.; Elzatahry, A.; Zhang, X.; Liu, Y.; Al-Dhayan, D.; Xia, Y.; Zhao, D. Mesoporous TiO_2 Microspheres with Precisely Controlled Crystallites and Architectures. *Chem* **2018**, *4*, 2436-2450.

(3) Wang, H.; Jeong, H. Y.; Imura, M.; Wang, L.; Radhakrishnan, L.; Fujita, N.; Castle, T.; Terasaki, O.; Yamauchi, Y. Shape- and size-controlled synthesis in hard templates: sophisticated chemical reduction for mesoporous monocrystalline platinum nanoparticles. *J. Am. Chem. Soc.* **2011**, *133*, 14526-14529.

(4) Posthumus, W.; Magusin, P.; Brokken-Zijp, J. C. M.; Tinnemans, A. H. A.; van der Linde, R. Surface modification of oxidic nanoparticles using 3-methacryloxypropyltrimethoxysilane. *J. Colloid Interface Sci.* **2004**, *269*, 109-116.

(5) Stojanovic, D.; Orlovic, A.; Glisic, S. B.; Markovic, S.; Radmilovic, V.; Uskokovic, P. S.; Aleksic, R. Preparation of MEMO silane-coated SiO_2 nanoparticles under high pressure of carbon dioxide and ethanol. *J Supercrit Fluids* **2010**, *52*, 276-284.

(6) Kresse., G.; Furthmiiller., J. Efficiency of ab-initio total energy calculations for metals and semiconductors using a plane-wave basis set. *Comput. Mater. Sci.* **1996**, *6*, 15-50.

(7) Kresse., G.; Furthmiiller., J. Efficient iterative schemes for ab initio total-energy calculations using a plane-wave basis set. *Phys. Rev. B* **1996**, *54*, 11169-11186.

(8) Henkelman, G.; Uberuaga, B. P.; Jónsson, H. A climbing image nudged elastic band method for finding saddle points and minimum energy paths. *J. Chem. Phys.* **2000**, *113*, 9901-9904.

(9) Perdew., J. P.; K. Burke, M.; Ernzerhof. Generalized gradient approximation made simple. *Phys. Rev. Lett.* **1996**, *77*, 3865-3868.

(10) Lv, H.; Sun, L.; Xu, D.; Li, W.; Huang, B.; Liu, B. Precise Synthesis of Hollow Mesoporous Palladium–Sulfur Alloy Nanoparticles for Selective Catalytic Hydrogenation. *CCS Chem.* **2022**, *4*, 2854-2863.

(11) Lv, H.; Qin, H.; Ariga, K.; Yamauchi, Y.; Liu, B. A General Concurrent Template Strategy for Ordered Mesoporous Intermetallic Nanoparticles with Controllable Catalytic Performance. *Angew. Chem. Int. Ed.* **2022**, *61*, e202116179.

(12) Song, S.; Li, K.; Pan, J.; Wang, F.; Li, J.; Feng, J.; Yao, S.; Ge, X.; Wang, X.; Zhang, H. Achieving the Trade-Off between Selectivity and Activity in Semihydrogenation of Alkynes by Fabrication of (Asymmetrical Pd@Ag Core)@(CeO_2 Shell) Nanocatalysts via Autoredox Reaction. *Adv. Mater.* **2017**, *29*, 1605332-1605338.

(13) Zhong, Y.; Liao, P.; Kang, J.; Liu, Q.; Wang, S.; Li, S.; Liu, X.; Li, G. Locking Effect in Metal@MOF with Superior Stability for Highly Chemoselective Catalysis. *J. Am. Chem. Soc.* **2023**, *145*, 4659-4666.

(14) Yang, N.; Cheng, H.; Liu, X.; Yun, Q.; Chen, Y.; Li, B.; Chen, B.; Zhang, Z.; Chen, X.; Lu, Q.; et al. Amorphous/Crystalline Hetero-Phase Pd Nanosheets: One-Pot Synthesis and Highly Selective Hydrogenation Reaction. *Adv. Mater.* **2018**, *30*, e1803234.

(15) Beier, M. J.; Andanson, J.-M.; Baiker, A. Tuning the Chemoselective Hydrogenation of Nitrostyrenes Catalyzed by Ionic Liquid-Supported Platinum Nanoparticles. *ACS Catal.* **2012**, *2*, 2587-2595.

(16) Macino, M.; Barnes, A. J.; Althahban, S. M.; Qu, R.; Gibson, E. K.; Morgan, D. J.; Freakley, S. J.; Dimitratos, N.; Kiely, C. J.; Gao, X.; et al. Tuning of catalytic sites in Pt/TiO_2 catalysts for the chemoselective hydrogenation of 3-nitrostyrene. *Nat. Catal.* **2019**, *2*, 873-881.
(17) Wei, H.; Liu, X.; Wang, A.; Zhang, L.; Qiao, B.; Yang, X.; Huang, Y.; Miao, S.; Liu, J.; Zhang, T. FeO_x-supported platinum single-atom and pseudo-single-atom catalysts for chemoselective hydrogenation of functionalized nitroarenes. *Nat. Commun.* **2014**, *5*, 5634.
(18) Yao, K.; Li, T.; Zhao, C.; Lu, W.; Zhao, S.; Wang, J. Au_3Pd_1 Nanodendrites with Hyperbranched Architectures: Green Synthesis at Room Temperature and Highly Selective Hydrogenation for 4-Nitrophenylacetylene. *ACS Sustain. Chem. Eng.* **2020**, *8*, 14914-14926.
